# Long oligos: direct chemical synthesis of genes with up to 1728 nucleotides†

**DOI:** 10.1039/d4sc06958g

**Published:** 2024-12-18

**Authors:** Yipeng Yin, Reed Arneson, Yinan Yuan, Shiyue Fang

**Affiliations:** a Department of Chemistry,, and Health Research Institute, Michigan Technological University Houghton Michigan 49931 USA shifang@mtu.edu; b College of Forest Resources and Environmental Science, Michigan Technological University Houghton Michigan 49931 USA yinyuan@mtu.edu

## Abstract

The longest oligos that can be chemically synthesized are considered to be 200-mers. Here, we report direct synthesis of an 800-mer green fluorescent protein gene and a 1728-mer *Φ*29 DNA polymerase gene on an automated synthesizer. Key innovations that enabled this breakthrough include conducting the synthesis on a smooth surface rather than within the pores of traditional supports, and the use of the powerful catching-by-polymerization (CBP) method for isolating the full-length oligos from a complex mixture. Conducting synthesis on a smooth surface not only eliminated the steric hindrance that would otherwise prevent long oligo assembly, but also, surprisingly, drastically reduced synthesis errors. Compared with the benchmark PCR assembly gene synthesis method, the direct long oligo synthesis method has the advantages of higher probability to succeed, fewer sequence restrictions, and being able to synthesize long oligos containing difficult elements such as unusually stable higher-order structures, long repeats, and site-specific modifications. The method is expected to open doors for various projects in areas such as synthetic biology, gene editing, and protein engineering.

## Abstract 2

Low steric hindrance, low error rate: conducting synthesis on a smooth surface made direct synthesis of 1000-nucleotide oligos a reality.

## Graphical abstract

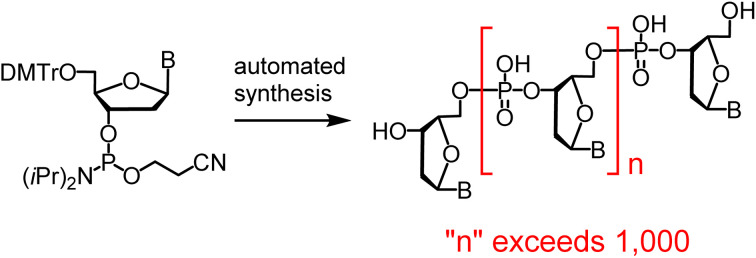

## Introduction

Many areas such as synthetic biology,^1^ nucleic acid vaccines,^2^ oligonucleotide therapeutics,^3^ CRISPR Cas9 gene editing,^4^ and protein engineering,^5^ require *de novo* synthesis of DNAs. In many of the cases, the DNAs need to be longer than 200 nucleotides (nt).^6^ Because the state of the art chemical synthesis methods cannot reliably produce oligos longer than 200 nt,^7^ the DNAs have to be produced *via* biological means such as PCR assembly or less commonly, ligation using synthetic oligos shorter than 200-mers.^8^ While biological methods have provided the required DNAs for the areas to emerge and advance, many sequences desperately needed by some projects are beyond the reach of existing methods.^9^ For example, if a sequence contains higher order structures with unusual stability, the PCR assembly method may not function effectively. If a sequence contains repeating segments that are longer than the short oligos available from chemical synthesis, biological means would not be able to accurately assemble the target DNA. If a sequence contains one or more site-specific modifications such as m^6^A, the PCR assembly method may not be able to produce the sequence. To overcome these and other challenges, which were discussed in a previous article,^10^ there is a need to develop new methods for direct *de novo* synthesis of oligos longer than 200-mers.

The most notable achievement in the area of *de novo* long oligo synthesis in recent years is the development of the template-independent enzymatic oligo synthesis (TiEOS) technologies, primarily utilizing engineered terminal deoxynucleotidyl transferases (TdT).^7,11–15^ While these technologies hold great promise, they are not without shortcomings. For example, the large enzyme-to-nucleotide mass ratio is not atomically economic, which may be one of the reasons for the high cost of the methods if the enzyme is not used in a catalytic quantity or recycled. The higher-order structures of long oligos may reduce synthesis efficiency.^12^ The coupling time may be lengthy, and the coupling yield may not meet the expectations for typical enzymatic reactions.^12^ In addition, enzymatic methods typically lack a capping step, increasing the likelihood of deletion errors.^13^ The TdT enzyme exhibits inherent nucleotide biases, leading to lower coupling efficiency for certain nucleotides, a problem that may be difficult to overcome through enzyme engineering.^12^ Finally, the method may be difficult to be adapted for synthesizing long oligos with site-specific modifications.

**Scheme 1:**
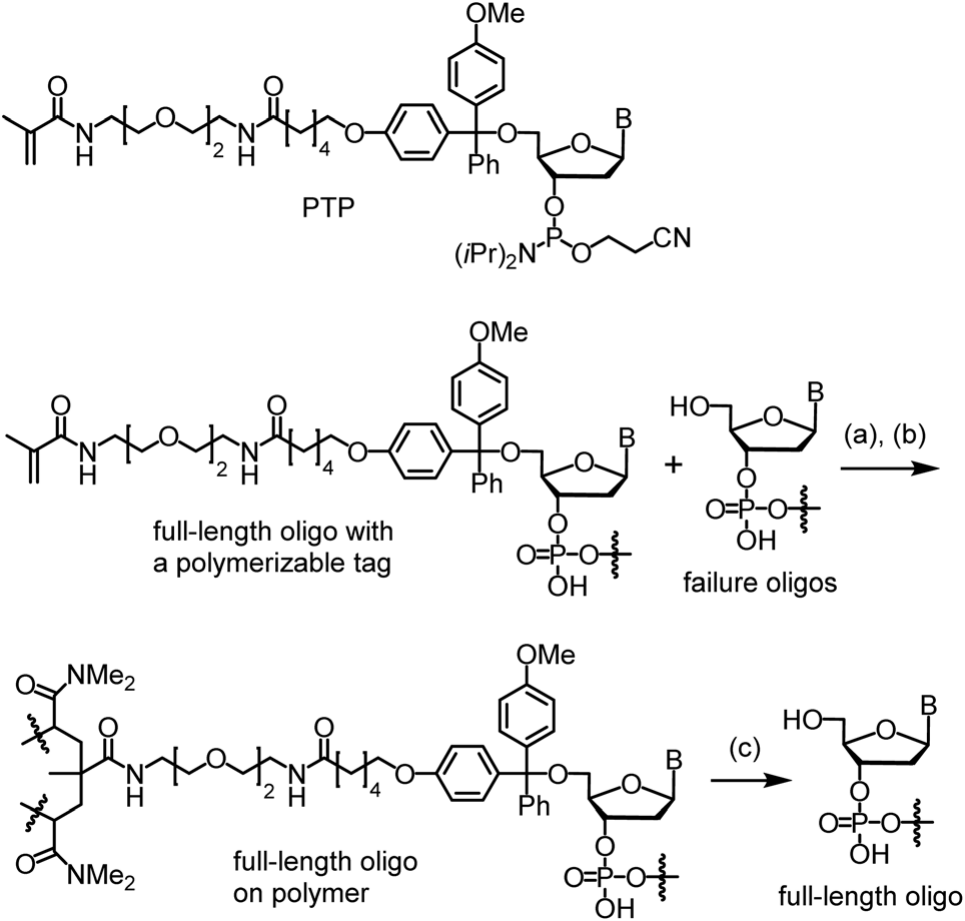
Catching-by-polymerization (CBP). Conditions: (a) *N*,*N*-dimethylacrylamide, *N*,*N*′-methylenebis(acrylamide), sodium acrylate, ammonium persulfate, *N*,*N*,*N*′,*N*′-tetramethylethylenediamine (TMEDA), rt, 1 h. (b) Wash. (c) AcOH (80%), rt, 5 min × 3. PTP = polymerizable tagging phosphoramidite. B = nucleobase.

In contrast to the resources invested in developing enzymatic methods for long oligo synthesis, little effort has been dedicated to advancing chemical methods over the past decade, even though many of the aforementioned shortcomings of enzymatic methods may be addressable using chemical approaches. Since 2010, our research team has been making efforts to develop a method called catching-by-polymerization (CBP) for synthetic oligo purification (Scheme 1).^16^ The method involves tagging the full-length oligo with a polymerizable tagging phosphoramidite (PTP) and incorporating the conjugate into a polyacrylamide gel. Because failure oligos are capped during automated synthesis, they are not tagged, and therefore are not incorporated into the gel. Oligo purification can thus be achieved by washing away the failure oligos, followed by cleaving the full-length oligo from the gel. Recognizing the power of the CBP method, we attempted to use it to isolate the extremely low percentage (but sufficient quantities) of full-length oligos from the complex mixture generated from the thousands of reactions required for long oligo synthesis.^17,18^ Most recently, using the CBP method, we succeeded in purification of 400-mers. Sanger sequencing confirmed the sequences.^10^ Here, with additional innovations involving the use of glass wool and glass beads as a solid support for long oligo synthesis, we report direct chemical synthesis of the 800-mer green fluorescent protein (GFP) gene and the 1728-mer *Φ*29 DNA polymerase gene, and their isolation with CBP and characterization with Sanger sequencing.

## Results

Previously, we successfully synthesized 400-mer oligos and employed the CBP method to isolate the full-length sequences from the complex mixture of crude products.^10,19^ The key elements that contributed to the success include using controlled pore glass (CPG) with pore sizes as large as 2000 Å as the solid support and reducing its loading by inactivating a portion of the reactive sites. These adjustments reduced the steric hindrance of the CPG, which we believe is crucial for the efficient synthesis of long oligos. Realizing the unlimited power of the CBP method for isolating low percentages of full-length oligos in the course of that work, we reasoned that if the steric hindrance can be further reduced, even longer oligos can be synthesized and isolated. Instead of reducing the loading of CPG further, we considered conducting the synthesis on a smooth surface as opposed to within pores of solid supports. A typical concern for this approach is insufficient loading, which is the reason for traditional oligo synthesis to be conducted in pores.

With the potential loading problem in mind, we thought that glass wool would partially solve the problem. Therefore, we calculated the loading of glass wool, and made a comparison with that of glass beads (Table 1).

**Table 1:** Loading of solid supports, and yields of long oligo synthesis

Entry	Items	Glass wool^a^	Glass beads	CPG 2K Å tested	CPG 2K Å	Wang resin
1	Density^b^	2.2 g ml^−1^	2.2 g ml^−1^			
2	Diameter^c^	8 μm	58 μm			
3	Loading formula^d^	10.6 ÷ (*d* × *r*) μmol g^−1^	15.9 ÷ (*d* × *r*) μmol g^−1^			
4	Loading	1.208 μmol g^−1^^e^	0.249 μmol g^−1^^e^	5.405 μmol g^−1^^f^	20–30 μmol g^−1^^g^	0.3–2.5 mmol g^−1^^g^
5	Relative loading	4.8	1	22	∼100^h^	∼10 000^i^
6	Measured loading^j^	0.981 μmol g^−1^	0.256 μmol g^−1^	5.359 μmol g^−1^		
7	800-mer synthesized^k^	3.7 nmol g^−1^	0.034 nmol g^−1^			
8	800-mer yield	3.7/981 = 0.38%	0.034/256 = 0.013%			
9	1728-mer synthesized^k^		0.041 nmol g^−1^			
10	1728-mer yield		0.041/256 = 0.016%			

a The length of glass wool can be 1 cm or longer.

b Density is that of solid glass.

c To achieve close to resistance-free flow of liquid, the diameter of glass beads needs to be ∼50 μm or larger.

d The effect of the length of glass wool on loading is minimal and is omitted in the formula. The units for *d* and *r* in the formulae are g ml^−1^ and μm, respectively. Details for deriving the formulae are in the ESI.

e Calculated value assuming 3.2 molecules per nm^2^.

f Given by the manufacturer of the CPG tested.

g Values from the literature.^20,21^

h 25 μmol g^−1^ is used for the calculation.

i 2.5 mmol g^−1^ is used for the calculation.

j Measure using trityl assay using the literature procedure.^20^

k Oligo obtained per gram of solid support after CBP purification as determined with a Qubit 4 Fluorometer.

Glass wool with a diameter of ∼8 μm is commercially available and inexpensive. We tested its resistance to liquid flow, and found that it is virtually resistance-free, which is required for solid phase synthesis. Assuming a length of 1 cm, density of 2.2 g ml^−1^, and 3.2 molecules per nm^2^ (Table 1),^22^ the loading is 1208 nmol g^−1^ (see the ESI† for calculations). For glass beads, to allow for close to resistance-free liquid flow, ideally their diameter is ∼50 μm or larger. Assuming a diameter of 58 μm and a density of 2.2 g ml^−1^, the loading is calculated to be 249 nmol g^−1^. Therefore, the loading of glass wool is ∼4.8 times that of glass beads (entry 5).

For comparison, the loading of commercial CPG with 2000 Å diameter is typically 20–30 μmol g^−1^,^20^ which is ∼100 times higher than that of glass beads (entry 5). The loading of the Wang resin (widely used for peptide synthesis) is 0.3–2.5 mmol g^−1^,^21^ which is close to 10 000 times higher. However, for long oligo synthesis, we reasoned that low loading is less of an issue. For most biological applications, as little as 1 pmol oligo is sufficient.^23,24^ Using glass wool, with a 100 mg support, which is the quantity that can be directly used under typical small scale oligo synthesis conditions, assuming an average stepwise yield of 99.7%, which corresponds to an overall yield of 0.25% for a 2000-mer synthesis, the quantity of full-length oligo is ∼296 pmol, which is much larger than 1 pmol. However, the low percentage yield is a serious problem because there is no method to purify or concentrate the full-length oligo. For example, HPLC would not be able to resolve the full-length oligo from failure ones. Gel electrophoresis would not be able to resolve this either and even if it could be engineered to resolve, the full-length oligo would be invisible on the gel due to its low percentage. Solid phase extraction methods^25–28^ may not be suitable for the task either because the high entropy barrier for reactions between large molecules and reactive sites on a solid surface would make the extraction inefficient, and it may be difficult for the large molecules to enter the pores of the solid support in the first place. However, using CBP, the low percentage problem can be overcome. With these considerations, we went ahead and synthesized long oligos on glass wool using the 800-mer GFP gene as an example.

### Glass wool functionalization

**Scheme 2:**
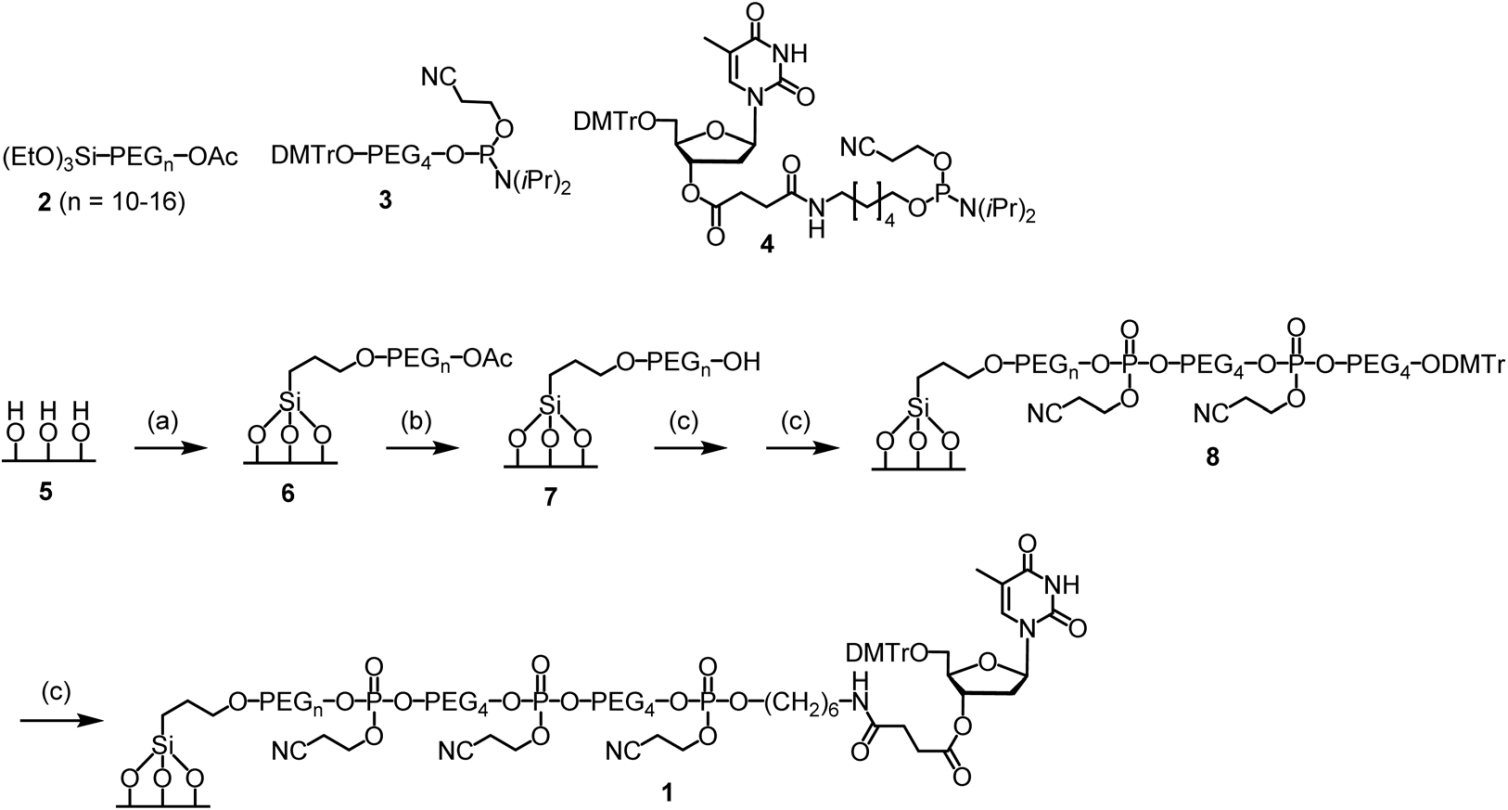
Functionalization of glass wool and glass beads. Conditions: (a) 2 (1% PhMe), rt, 20 min; then, supernatant removed, glass wool 100 °C, 4 h. (b) NH_4_OH (30%), 55 °C, 2 h. (c) On a DNA synthesizer, standard coupling, oxidation and deblocking conditions with modifications; see the ESI† for details.

To conduct the synthesis, the functionalized glass wool 1 was prepared (Scheme 2). The required compounds 2 and 3 were purchased from commercial sources. Compound 4 was synthesized on-site (ESI and Scheme S1†). Glass wool (5) was activated by treating with a piranha solution under previously used conditions.^29^ The surface was then PEGylated using 2 to give 6, the acetyl group of which was removed with ammonia to give 7.^30^ For solid phase synthesis, when the reaction site is close to the surface, the reaction is less efficient. Therefore, the linker in 7 was elongated using compound 3. The elongation reactions were carried out on a MerMade 6 synthesizer under typical DNA synthesis conditions but with a longer reaction time. After two cycles, 8 was obtained, which was converted to the target functionalized glass wool 1 under similar conditions on the synthesizer using compound 4 as the phosphoramidite monomer. More details regarding the synthesis are provided in the ESI.† The success of the surface functionalization reactions could be estimated timely by observing the color of trityl cations during the detritylation steps (Fig. S1†). The loading of the glass wool thus functionalized was determined to be 981 nmol g^−1^ (entry 6, Table 1) using a reported method,^20^ which is not far away from the calculated value of 1208 nmol g^−1^ (entry 4).

### Synthesis of an 800-mer oligo on glass wool

The synthesis of the 800-mer GFP gene on glass wool was carried out under typical conditions of phosphoramidite chemistry with little modification. The functionalized glass wool (30 mg, 29.4 nmol) was packed into an empty 0.2 μmol synthesis column. Even though the scale of the synthesis was only 29.4 nmol, to ensure complete coverage of the glass wool in every reaction in the entire synthesis, the synthesizer manufacturer recommended 1 μmol synthetic cycle was used. While the conditions may be engineered to minimize reagent use considering that less reagent is needed to fill the synthesis column for the case of glass wool, which does not need a reagent to fill pores, than that of traditional porous supports, that engineering studies was not pursued in the present study. The synthesis was tested on both ABI-394 and MerMade 6 synthesizers. Even though the former consumed more solvents, it was preferred due to its shorter synthesis time. In principle, the synthesis can be carried out consecutively, which would only need a little more than three days, we had to pause the synthesis two or more times to refill reagents. With the pauses, the synthesis was completed within four days. Trityl assay consistently gave 99.6% to 99.8% average stepwise yields once the synthetic cycle ran over 100 times consecutively. To tag the full-length oligo for CBP purification, the last nucleotide at the 5′-end of the 800-mer was not included in the above synthesis procedure. Instead, the 799-mer with a trityl group at its 5′-end on glass wool was transferred to a column suitable for a MerMade 6 synthesizer. Upon delivery of the deblocking agent, the orange color characteristic of dilute trityl cations could still be observed indicating the existence of full-length sequences even after 799 synthetic cycles (Fig. S2†).

The last nucleotide was introduced with PTP on the MerMade 6 synthesizer, which also tagged the full-length sequences with a methacrylamide group. Details are given in the ESI.† For deprotection and cleavage, the glass wool was first treated with 10% DBU in ACN, which removed the 2-cyanoethyl groups. Treating with concentrated NH_4_OH under typical oligo deprotection conditions gave a mixture of 5′-tagged full-length oligo and un-tagged failure sequences as well as other impurities (Scheme 1). CBP purification was then carried out by co-polymerizing the tagged full-length oligo into a polyacrylamide gel. The failure oligos and many other types of impurities were removed by washing. This gave only the full-length oligo on the polymer. The full-length oligo was then cleaved from the gel using 80% AcOH. After removing the acid, the oligo may be precipitated with *n*BuOH from an NH_4_OH solution. This is important for avoiding oligo damage by residue acid if the oligo needs to be stored before use. Otherwise, precipitation may be omitted. The quantity of the oligo was determined to be 27.4 μg (111 pmol) for the synthesis involving 30 mg glass wool. The overall yield for the entire 800-mer synthesis and purification was 0.38% (entry 8, Table 1).

### Characterization of the 800-mer oligo synthesized on glass wool

**Fig. 1:**
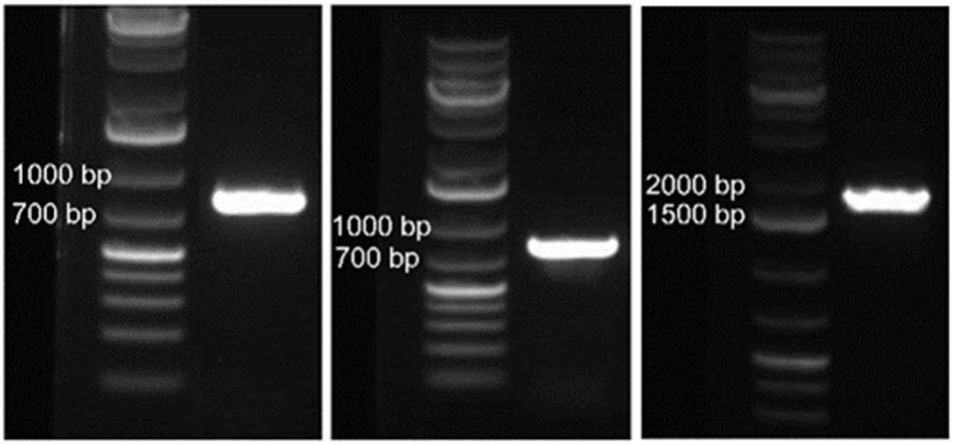
Gel electrophoresis images of PCR products using CBP-purified oligos, 800-mer synthesized on glass wool (left), 800-mer synthesized on glass beads (middle), and 1728-mer synthesized on glass beads (right), as the template.

**Fig. 2:**
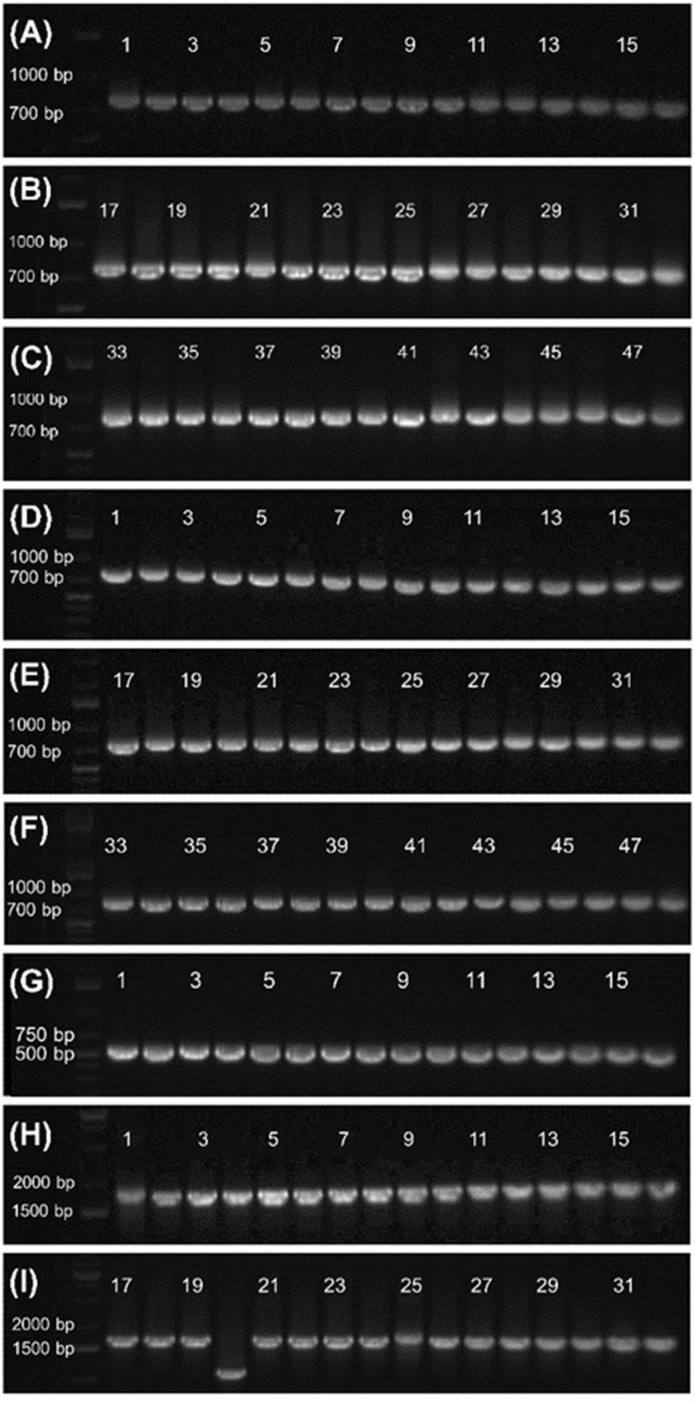
Gel electrophoresis images of colony PCR products. (A–C) Originated from 800-mer synthesized on glass wool. (D–F) Originated from 800-mer synthesized on glass beads. (G) Originated from 1728-mer synthesized on glass beads. The primers for colony PCR only covered 600 nt of the oligo. (H and I) Originated from 1728-mer synthesized on glass beads. The primers for colony PCR covered the entire oligo. For all the colonies analyzed, all except for lane 20 of (I) contained the anticipated band, evidence of the high reliability of the long oligo synthesis method.

The 800-mer oligo was characterized with Sanger sequencing. For this purpose, a portion (30 ng) of the CBP-purified 800-mer gene was PCR amplified using high fidelity DNA polymerase. The product was analyzed with agarose gel electrophoresis. As shown in Fig. 1, a band corresponding to 800-mer can be clearly observed. A portion of the PCR product was ligated into the pCR™4Blunt-TOPO™ vector and transformed into chemically competent *E. coli* cells. The transformed cells were grown on agar plates. Colony PCR was performed on selected cell colonies. The PCR products were analyzed with agarose gel electrophoresis. As shown in Fig. 2A–C, all the 48 colonies contained an expected band. Plasmids of the colonies were subjected to Sanger sequencing. The alignment of the sequencing data with the reference sequence is provided in the ESI.† The results are summarized in Table 2. Among the 48 colonies sequenced, 41 contained the correct sequence, corresponding to a success rate of 85% (entries 1–3). The errors in the incorrect sequences include three substitutions, four single nucleotide deletions and one 10 nt deletion (entries 6, 7 and 9–11). The rates for the different errors were all lower than 0.003% except for single nucleotide deletion, which had a rate of 0.0104%. The sum of the error rates was 0.0208% (entry 13).

**Table 2:** Summary of sequencing results

Entry	Oligo sample	800-mer from glass wool	800-mer from glass beads	1728-mer from glass beads	1^st^ 1000 nt of the 1728-mer	Literature error rates^a^
1	Total colonies sequenced	48	47	16	16	
2	Colonies with the correct sequence	41	45	7	14	
3	Rate of the correct sequence	85%	96%	44%	88%	
4	G-to-A substitution/error rate^b^	0	0	0	0	0.11%^c^
5	G-to-T substitution/error rate	0	0	3/0.0109%	1/0.0063%	0.03%
6	C-to-T substitution/error rate	1/0.0026%	0	0	0	0.02%
7	T-to-C substitution/error rate	1/0.0026%	0	1/0.0036%	0	0.01%
8	A-to-G substitution/error rate	0	0	1/0.0036%	0	0.01%
9	A-to-T substitution/error rate	1/0.0026%	0	0	0	<0.01%
10	Single nt deletion/error rate	4/0.0104%	1/0.0027%	3/0.0109%	1/0.0063%	0.4%^d^
11	Block deletion/error rate	One 10 nt deletion/0.0026%	One 2 nt deletion/0.0027%	Two 2 nt deletion/0.0072%	0	No data
12	Single nt insertion/error rate	0	0	2/0.0072%	0	0.00–0.01%^e^
13	Total error rate^f^	0.0208%	0.0054%	0.0434%	0.0126%	0.58%

a Data were from sequencing the 20^th^ to 48^th^ nucleotide region of chemically synthesized 85-mers. Oligo synthesis conditions: activation, 1*H*-tetrazole in ACN; capping, Ac_2_O in THF, 10% 1-methylimidazole in 10% pyridine/THF; oxidation, 0.02 M I_2_ in THF/pyridine/H_2_O; deblocking, 3% TCA in DCM. For more details, see ref. 31.

b The error rates were calculated by dividing the number of errors by the total number of nucleotides subjected to sequencing. For example, for the 1728-mer synthesized on glass beads, a total of three G-to-A substitutions were found in the data of sequencing 16 colonies; the error rate is 3 ÷ (1728 × 16) = 0.0109%.

c When DCI was used as the activator, the error rate was lower.^31^

d 0.1% for each nucleotide.

e dA 0.005%, dC 0.003%, dG 0.008%, T 0.002%.

f The total error rate is the sum of individual error rates. It does not represent the probability for a specific nucleotide position in a sequence to have substitution, deletion, addition and other errors.

### Synthesis of an 800-mer oligo on glass beads and oligo characterization

Although the loading of glass beads is predicted to be ∼4.8 times lower than that of glass wool, the quantity of oligos produced on them under typical small scale synthesis conditions is still predicted to be higher than 1 pmol, a quantity sufficient for most biological applications.^23,24^ For example, with 100 mg glass beads with a loading of 256 nmol g^−1^, assuming an average stepwise yield of 99.7%, which corresponds to an overall yield of 0.25% for a 2000-mer synthesis, the quantity of full-length oligo is ∼64 pmol. Considering that glass beads are easier to handle and less likely to generate fine particles that may block the synthesizer, we decided to test long oligo synthesis on glass beads for the purpose of comparison with glass wool. The glass beads were functionalized using the same procedure for functionalizing glass wool (Scheme 2). The loading was determined to be 256 nmol g^−1^ (entry 6, Table 1), which is close to the calculated value of 249 nmol g^−1^ (entry 4).

Oligo synthesis was conducted under the same conditions using glass wool as the support. The scale was 12.8 nmol, for which 50 mg glass beads were used. Deprotection and cleavage as well as CBP purification were also the same except that only 20 mg (theoretically 5.12 nmol oligo) glass beads were used. The quantity of the oligo obtained was determined to be 168 ng (0.68 pmol) for the synthesis involving 20 mg glass beads. The overall yield for the entire 800-mer synthesis and purification was 0.013% (entry 8, Table 1), which is lower than 0.38% for glass wool. The reason is unclear but may be attributable to the loss of materials in the deprotection and purification process probably due to the increased difficulty to handle smaller quantities of oligos.

The CBP purified 800-mer was also subjected to PCR, cloning and Sanger sequencing. The image of the gel for electrophoresis analysis of the PCR product is shown in Fig. 1. Even though the quantity of oligos was much lower, the band corresponding to the 800-mer is clear. The image of the gel for analysis of colony PCR products is shown in Fig. 2D–F. As can be seen, all colonies selected for the analysis had the 800-mer sequence. Plasmids of 47 colonies were subjected to Sanger sequencing. The data are provided in the ESI.† The results are summarized in Table 2. Among the 47 colonies sequenced, 45 contained the correct sequence, which was 96% (entries 1–3). The errors in the incorrect sequences only include one deletion and one 2 nt deletion (entries 10 and 11). The rates for both errors were 0.0027%. The sum of the error rates was 0.0054% (entry 13).

### Synthesis of a 1728-mer oligo on glass beads and oligo characterization

Encouraged by the success of the 800-mer syntheses, considering that the CBP method can potentially pick up oligos with unlimited length from a crude mixture with unlimited complexity, we decided to synthesize the 1728-mer *Φ*29 DNA polymerase gene. Because glass beads had close to zero errors, and had a higher percentage of correct sequences than glass wool (96% *vs.* 85%) for the 800-mer syntheses, glass beads were chosen for the synthesis. The synthesis, deprotection, and CBP purification procedures as well as PCR, cloning, and sequencing were the same as described for glass wool. The scale of the synthesis was 33.28 nmol, which corresponds to 130 mg glass beads. The quantity of oligo obtained was 2.83 μg (5.28 pmol) for the synthesis using 130 mg glass beads (41 pmol g^−1^, entry 9, Table 1). The overall yield for the entire 1728-mer synthesis and purification was 0.016% (entry 10), which is similar to that of 800-mer synthesis using glass beads as the support.

The image of the gel for electrophoresis analysis of the PCR product of the CBP purified 1728-mer is shown in Fig. 1. As can be seen, the expected band can be clearly observed. Colony PCR was first conducted on 16 colonies using primers targeting only a portion of the 1728-mer (see the ESI†). All colonies were found to have the gene (Fig. 2G). Plasmids of the 16 colonies were subjected to Sanger sequencing. Sequencing data are provided in the ESI,† and the results are summarized in Table 2. Among the 16 colonies sequenced, 7 contained the correct sequence, which corresponds to a success rate of 44% (entries 1–3). The errors in the incorrect sequences include five substitutions, three single nucleotide deletion, two 2 nt deletion and 2 single nucleotide insertion (entries 5, 7, 8, 10 and 11). The sum of the error rates was 0.0434% (entry 13). We also performed gel electrophoresis on colony PCR products of additional colonies using primers covering the entire 1728-mer. Among 32 colonies, only one did not show the expected band (Fig. 2H and I).

## Discussion

For long oligo synthesis, besides the challenge of isolating full-length sequences from a complex mixture, another hurdle is synthesis errors, which include deletions, insertions and substitutions.^31^ For the two 800-mer syntheses, the one on glass beads is significantly better (Table 2). For the 47 colonies sequenced, only two errors were found. The total error rate was 0.0054%. The synthesis on glass wool had more errors. The total error rate was 0.0208%, which was about four times higher. The reason may be attributable to the less homogeneous reaction environment during synthesis for glass wool. We did not cut glass wool into short segments because of the concern of generating debris that may block the synthesizer. As a result, the glass wool was not freely movable during reagent delivery. In contrast, the reaction environment for glass beads is more homogeneous. For the synthesis results, it is remarkable that for the first 1000 nt assembly for the 1728-mer synthesis, only two errors were found for the 16 sequences that were sequenced (Table 2). For nucleotides beyond 1000 nt, the error rate was slightly higher, but it was still surprising low.

Compared with results in the literature using CPG as the support,^31^ error rates in the present work were drastically lower (Table 2). For example, among the most frequent substitution errors, which include G-to-A, G-to-T, C-to-T, T-to-C, and A-to-G (entries 4–8),^31^ the highest G-to-A substitution was completely eliminated in the present work (entry 4). For all other errors, the rates were also lowered. The sum of the rates of substantial errors for literature syntheses is 0.58%, while that for the present syntheses is less than 0.0434%, which is more than 10 times lower (entry 13). It is noted that the error rates for the present work were from 800-mer and 1728-mer synthesis, while the numbers from the literature was from syntheses of oligos shorter than 100-mer. It is known that error rates increase as oligos grow longer. The increased accuracy of the syntheses on a smooth surface compared with within the pores of CPG may be attributed to higher reaction kinetics in the case of the former. The assumption that reactions on a smooth surface have better kinetics is consistent with discussions in a 1987 patent by Benner.^32^

As mentioned earlier, we successfully synthesized 401-mer and 399-mer oligos on CPG.^10^ Compared with that work, the present results are also much better. For the prior study, the bands corresponding to the full-length oligos after PCR amplification of CBP purified oligos were weak (see Fig. 2 in ref. 10) while the bands for the present work are strong (Fig. 1). The gel images of colony PCR results also provided evidence of superiority of the present work. For the prior study, according to gel images, plasmids from 26 out of 64 colonies could be readily estimated not to contain the full-length sequence of the target oligo (see Fig. 3 in ref. 10). For the present work, plasmids from 143 out of 144 colonies that were subjected to the analysis could be estimated to contain the expected sequence. For the prior study, six plasmids that were estimated to contain the full-length sequence based on gel analysis were subjected to Sanger sequencing. Two sequences were correct. Later, we intentionally sequenced 14 additional plasmids that were estimated to contain only a portion of the desired sequence.^33^ These sequences were found to contain one or more blocks of deleted nucleotides. The deleted blocks ranged from 8 to over 100 nucleotides. For the 20 sequenced sequences, besides the block deletions, other errors include 11 single nucleotide deletions, and three G-to-A and one T-to-C substitutions. The sequencing data are in the ESI.† Comparing these data with the present ones, it is evident that the major problem for synthesizing long oligos on porous supports is block and single nucleotide deletions. In addition, G-to-A substitution is much more likely to occur with porous supports. The comparison indicates that for long oligo synthesis, supports with a smooth surface should be used.

With the long oligo synthesis results that far exceed the expectations of many researchers including us, one may wonder how this is possible considering the many widely recognized side reactions of oligo synthesis. For example, the acetic anhydride capping efficiency is estimated to be ∼90%.^31^ Assuming a coupling efficiency of 99%, the deletion sequence in our products would be ∼0.1%, far higher than the 0.002–0.01% range we observed (entry 10, Table 2). The total detritylation time under acidic conditions for the 1728-mer synthesis is ∼47 hours. Common intuition would suggest high levels of depurination in the products. While answers to these questions are hard to obtain, for the former, it is possible that conducting the synthesis on a smooth surface not only improved the yield of coupling, but also drastically improved the yield of capping. For the latter, occasional exposure of oligos to acid might have less of an effect on depurination than constant acid exposure. It is also possible that depurination may be more likely in the pores than on a smooth surface under the same acidic conditions. Furthermore, it is also possible that depurination might have occurred, but it was not detected by our analysis method. The depurinated oligos were broken under the basic conditions of oligo deprotection and cleavage. The 3′-fragments were washed away during CBP, and the 5′-fragment had to compete with the excess primer for PCR amplification.

It is noted that although the current paper is presented in the context of synthesis of genes, which are double-stranded (ds) DNAs, the long oligo synthesis method can also be used to obtain single-stranded (ss) long oligos or to obtain oligos with site-specific modifications. In these contexts, PCR, cloning and Sanger sequencing should only be carried out using a small portion (*e.g.* about 10 pmol) of the synthesized and CBP purified oligos for the purpose of characterization because PCR and cloning would convert ss oligos to ds oligos, and eliminate the site-specific modifications. The remaining portion (more than 50 pmol) can then be used for the intended applications assuming that the sequence error rates are low, and the errors can be tolerated by the applications. If error-free ss oligos with or without site-specific modifications are required, sequences containing errors could be removed by patching error sites with short oligos and then using immobilized MutS to remove the sequences with errors.^34,35^ The short patching oligos in the remaining error-free ss oligos can then be easily removed using size-exclusion filtration, solid phase reversible immobilization (SPRI) bead extraction,^36^ or other methods.

## Conclusions

In summary, we demonstrated that direct chemical synthesis of oligos with over 1000 nucleotides can be reliably achieved. The innovations that made this possible include conducting synthesis on a smooth surface, and the use of CBP to isolate full-length sequences from a complex mixture. Conducting synthesis on a surface not only reduced steric hindrance, but also, surprisingly, drastically reduced errors. The longest oligo synthesized is the 1728-mer *Φ*29 polymerase gene. Given the high quality of data, we believe that the method can be used to synthesize even longer oligos. Synthesizing 1000-mer oligos has long been a major goal in nucleic acid chemistry. Now that this goal has been achieved, we anticipate that many projects in areas such as synthetic biology, protein engineering, and CRISPR Cas9 gene editing will become easier.

## Data availability

All data are available in the main text or the ESI.†

## Author contributions

Y. Yin (investigation, writing), RA (formal analysis, investigation, writing), Y. Yuan (conceptualization, funding acquisition, formal analysis, project administration, supervision, writing), and SF (conceptualization, funding acquisition, project administration, supervision, writing).

## Conflicts of interest

Michigan Technological University owns the IP associated with the work.

## Supplementary Material

SC-OLF-D4SC06958G-s001
