# Supplementary material for: Long oligos: direct chemical synthesis of genes with up to 1728 nucleotides
Source: Chem Sci. 2024 Dec 18;16(4):1966–73. doi: 10.1039/d4sc06958g (PMC11694485; doi:10.1039/d4sc06958g)
Supplement: SC-016-D4SC06958G-s001 [file SC-016-D4SC06958G-s001.pdf]

Supplementary Information (SI) for Chemical Science.
This journal is © The Royal Society of Chemistry 2024

Supplementary Information

Long oligos: direct chemical synthesis of genes with up to 1,728 nucleotides

Yipeng Yin,# Reed Arneson,# Yinan Yuan,* Shiyue Fang*

Department of Chemistry, and Health Research Institute, Michigan Technological University, 1400 Townsend Drive, Houghton, MI 49931, USA

College of Forest Resources and Environmental Science, Michigan Technological University, 1400 Townsend Drive, Houghton, MI 49931, USA

* Emails: yinyuan@mtu.edu and shifang@mtu.edu

# Equal contributors

Experimental Procedures

General information:

Glass wool (cat# G0034) was purchased from Flinn Scientific, Inc. Glass beads (Borosilicate Solid Glass Microspheres, 2.2 g/cc, 53-63 µm, cat#BSGMS-2.2) were purchased from Cospheric LLC. 2-[(Acetoxy(polyethyleneoxy)propyl]triethoxysilane (cat# SIA0078.0) was purchased from Gelest. Other small molecule chemicals for organic synthesis, oligo deprotection and cleavage, and catching-by-polymerization such as DBU, saturated NH_4OH, *N,N*-dimethylacrylamide, *N,N′*-methylenebis(acrylamide, ammonium persulfate, and *N,N,N′,N′*-tetramethylethylenediamine (TMEDA) were purchased from Aldrich or other companies. Reagents for oligo synthesis were purchased from Glen Research and Hongene Biotech. Oligos were synthesized on an ABI 394 DNA/RNA synthesizer. Some steps for the functionalization of glass wool and glass beads were performed on a MerMade 6 synthesizer.

Polymerase chain reactions (PCR) were performed using an Applied Biosystems 2720 Thermal Cycler. Gel images were obtained on a UVP GelDoc-IT Imaging System 2UV Transilluminator at 302 nm. Oligo primers were purchased from IDT. GelRed® (cat# 41003) was purchased from Biotium. Solid phase reversible immobilization (SPRI) bead (cat# CNGS005) was purchased from BullDog Bio. QIAprep® Spin Miniprep Kit (cat# 27106) and QIAquick® Gel Extraction Kit (cat# 28704) were purchased from Qiagen. dNTPs, Phusion Hot Start II High Fidelity DNA polymerase (cat# F549S), DreamTaq™ Hot Start Green PCR Master Mix (cat# K9021), TopVision agarose (cat# R0491), GeneRuler 1 KB+ ladder (cat# SM1334) Luria Broth powder (cat# 12795-027) and Zero Blunt™ TOPO™ PCR Cloning Kit (cat# 450031) were purchased from ThermoFisher. SOC medium (cat# B8020S), and NEB® 5-alpha cells (cat# C2987H) were purchased from New England Biolab. Sanger sequencing was carried out at MCLAB (https://mclab.com/).

No unexpected or unusually high safety hazards were encountered in the following experiments.

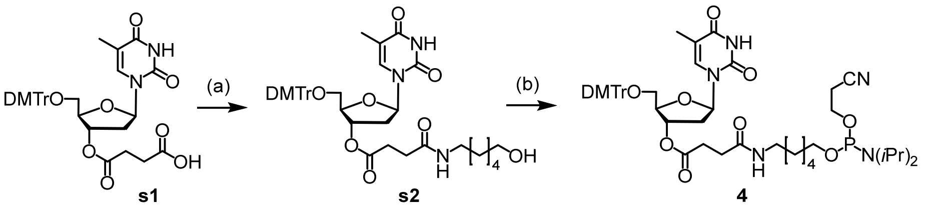

Scheme S1. Synthesis of linker phosphoramidite **4**. Conditions: (a) $H_2N(CH_2)_6OH$ (1.1 eq), HBTU (1 eq), DIEA (3 eq), DMF, rt, 8 h, 91%. (b) Diisopropylammonium tetrazolide (1.5 eq), *N,N,N',N'*-tetraisopropylphosphorodiamidite (1.5 eq), rt, 12 h, 87%.

To a solution of compound **s1** (2 g, 3.1 mmol, 1 eq) in dry DMF (20 mL) was added DIEA (1.2 g, 9.3 mmol, 3 eq) and HBTU (1.17 g, 3.1 mmol, 1 eq). The mixture was stirred under nitrogen at rt for 15 min. The content was transferred dropwise to flask containing the solution of 6-amino-1-hexanol (0.25 g, 3.41 mmol, 1.1 eq) in dry DMF (5 mL) via a cannula with its inlet terminus wrapped with cotton to prevent transfer of insoluble materials formed. After stirring at rt for 8 h, the mixture was concentrated to ~30 mL under vacuum from an oil pump. The residue was purified by dissolving the sample in the solvent mixture of acetone/hexane 3:1 with 5% Et_3N, loading onto a column (SiO_2), and eluting with the same solvent mixture. Compound **s2** was give as a pale yellow foam upon drying under high vacuum: 2.1 g, 91%; ^1H NMR (500 MHz, $CDCl_3$) δ 1.27-1.50 (m, 8H), 2.40-2.62 (m, 9H), 2.82 (s, 2H), 2.94 (s, 2H), 3.14-3.18 (m, 2H), 3.41 (s, 3H), 3.53-3.55 (t, J = 5.0 Hz, 1H), 4.11 (s, 1H), 5.43 (s, 1H), 6.33-6.37 (m, 1H), 6.78-6.80 (d, J = 10.0 Hz, 4H), 7.18-7.35 (m, 9H), 7.58 (s, 1H), 7.95 (s, 1H), 9.82 (s, 1H); ^{13}C NMR (126 MHz, $CDCl_3$) δ 8.7, 25.2, 26.2, 29.1, 30.5, 31.3, 32.2, 36.7, 47.2, 55.2, 62.4, 63.6, 75.4, 83.9, 84.3, 87.0, 111.4, 113.3, 127.1, 128.0, 130.0, 135.1, 135.7, 144.1, 150.6, 158.7, 163.1, 164.1, 171.4, 172.3. The compound is known.[1]

To a solution of compound **s2** (2 g, 2.6 mmol, 1 eq) in dry ACN was added diisopropylammonium tetrazolide (0.69 g, 4.0 mmol, 1.5 eq) and 2-cyanoethyl *N,N,N',N'*-tetraisopropylphosphorodiamidite (1.2 g, 4.0 mmol, 1.5 eq) at rt under nitrogen. After stirring overnight, the mixture was concentrated to dryness. The product was purified by dissolving in the solvent mixture of acetone/hexane 3:1 with 5% Et_3N, loading onto a column (SiO_2), and eluting with the same solvent mixture. The product was given as a white foam upon drying under high vacuum: 2.2 g, 87%; ^1H NMR (500 MHz, $CDCl_3$) δ 1.10-1.12 (t, J = 5.0 Hz, 12H), 1.18-1.54 (m, 8H), 2.40-2.68 (m, 8H), 3.12-3.16 (m, 1H), 3.40-4.08, (m, 14H), 5.43 (s, 1H), 6.25 (s, 1H), 6.32-6.39 (m, 1H), 6.76-6.78 (d, J = 10.0 Hz, 4H), 7.14-7.34 (m, 9H), 7.55 (s, 1H); ^{13}C NMR (126 MHz, $CDCl_3$) δ 11.5, 20.3, 22.8, 24.5, 25.5, 26.4, 29.3, 30.4, 31.0, 37.8, 39.4, 42.8, 45.2, 55.1, 58.2, 63.5, 75.4, 83.8, 84.2, 86.9, 111.4, 113.2, 117.8, 127.0, 128.0, 129.9, 135.1, 135.4, 144.1150.7, 158.6, 164.0, 171.0, 172.3; ^{31}P NMR (202 MHz, $CDCl_3$) δ 146.97, 147.01. The compound is known.[2]

Glass surface functionalization

Glass wool (2 g) from commercial source was soaked in a piranha solution (H_2SO_4/H_2O_2 3:1, v/v, ~20 mL) with occasional shaking at rt. Caution, piranha solution is highly reactive and corrosive; appropriate safety procedure must be followed. After 1 h, the supernatant was removed. The glass wool was washed sequentially with water and ACN each for 5 times, and then allowed to dry in the air.

The activated glass wool (**5**, 2 g, see Scheme 2 for the surface functionalization procedure) in the solution of 1% 2-[(acetoxy(polyethyleneoxy)propyl]triethoxysilane (**2**) in freshly distilled toluene (20 mL) was incubated at rt for 20 min. The supernatant was removed, and the glass wool was washed with toluene

5 times and then incubated in an oven at 100 °C for 4 h. After cooling to rt, the glass wool was washed with chloroform to give the functionalized glass wool **6**.

To remove the acetyl group, the glass wool **6** (1 g) was incubated in NH_4OH (30%, 10 mL) at 55 °C for 2 h. The supernatant was removed, and the glass wool was washed sequentially with water and ACN each for 5 times, and then allowed to dry in the air. This converted **6** to **7**.

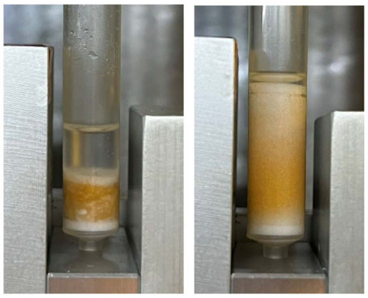

Figure S1. The trityl color on glass wool (left) and glass beads (right) during the detritylation steps for the conversion of **8** to **1**.

The glass wool (**7**, 1 g) was packed in a DNA synthesis column. On a MerMade 6 synthesizer, the following steps were carried out. Between the steps, the glass wool was washed with dry ACN. Coupling: **3** (0.5 mL, 0.1 M in ACN), DCI (0.5 mL, 0.25 M in ACN), 10 min waiting; repeat 4 times. Oxidation: I_2 (1 mL, 0.02 M in THF/pyridine/H_2O), 1 min waiting; repeat 2 times. Deblocking: DCA (1 mL, 2% in DCM), 90 sec waiting; repeat 3 times. The coupling and oxidation steps were repeated 1 time. This converted **7** to **8**. The deblocking (see Figure s1 for color), coupling (**4** instead of **3**), and oxidation steps were repeated. This converted **8** to **1**. The loading was determined to be 981 nmol/g using a method involving treating the glass wool with an acid and measuring the UV absorption of trityl cations.[3]

Glass beads (5 g) were functionalized using the same conditions as described above. The loading was determined to be 256 nmol/g. The functionalized glass beads are also represented by **1** in this paper.

Oligo synthesis: The synthesis of the 800-mer GFP gene on glass wool is used for the description (the sequences of the 800-mer GFP and 1,728-mer Φ29 DNA polymerase genes are given in the sequence alignment documents in the supporting information). The glass wool **1** (30 mg, 29.4 nmol) was packed in an empty 0.2 μmol synthesis column, and loaded onto an ABI 394 DNA/RNA synthesizer. The synthesizer manufacturer recommended 1 μmol synthetic cycle with slight modifications was used. The specific conditions were the following. ***Deblocking***: DCA (2% in DCM), 98 sec. ***Coupling***: Bz-dA, Ac-dC, *i*Bu-dG or dT phosphoramidite (0.1 M in ACN); DCI (0.25 M in ACN), 2.5 sec × 2 reagent delivery, 35 sec waiting. ***Capping***: Cap A, THF/pyridine/Ac_2O; cap B, 1-methylimidazole (16% in THF), 10 sec delivery, 10 sec waiting. ***Oxidation***: I_2 (0.02 M in THF/pyridine/H_2O), 8 sec delivery, 15 sec waiting. Washing conditions between the steps were the same as recommended by the synthesizer manufacturer except for an additional wash with ACN after the oxidation step to ensure complete washing. For purposes such as refilling reagents, the synthesis was set up as several shorter ones such as 200-mer. The average stepwise yields as indicated by the trityl assay of the synthesizer were consistently 99.6%, 99.7% or 99.8% after the syntheses reached over 100 synthetic cycles. The last nucleotide was incorporated with the polymerizable tagging phosphoramidite PTP, where B is thymine, on a MerMade 6 synthesizer (0.1 M in ACN, 0.25 M DCI in ACN, 5 min; repeat 3 times; followed by capping and oxidation as usual; no detritylation). The detritylation

of the 799th nucleotide before the coupling step involving PTP was also performed on the MerMade 6 synthesizer. The trityl color was visible (Figure S2).

Using glass beads as the solid support, the 800-mer GFP gene (50 mg glass beads, theoretically 12.8 nmol) and the 1,728-mer Φ29 DNA polymerase gene (130 mg glass beads, 33.28 nmol) were synthesized under the same conditions described above.

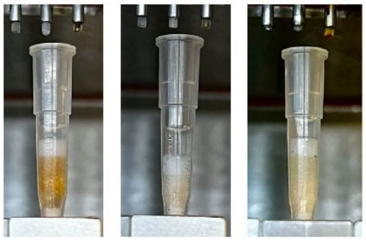

Figure S2. Images showing trityl color of last detritylation before coupling with PTP. The oligo synthesis was carried out on an ABI synthesizer, while the last detritylation and PTP tagging was carried out on a MerMade 6 synthesizer. Left: glass wool/800-mer synthesis. Middle: glass beads/800-mer synthesis. Right: glass beads/1,728-mer synthesis. The trityl color as well as the trityl assay data from the ABI synthesizer are useful for estimating the probability of success of the long oligo syntheses.

Oligo deprotection and cleavage: The deprotection and cleavage of the 800-mer GFP gene on glass wool is used for the description. To the glass wool (~30 mg, theoretically 29.4 nmol oligo) in a 1.5 mL centrifuge tube was added DBU (10% in ACN). The tube was gently shaken at rt for 10 min. The supernatant was removed, and the DBU treatment was repeated 1 time. After the supernatant was removed, the glass wool was washed with ACN for 5 times. Saturated NH_4OH (0.5 mL) was added. The tube was sealed and heated at 55 °C for 16 h. After cooling to rt, the supernatant was transferred to a clean centrifuge tube. The glass wool was washed with water (200 µL × 3). The supernatant and washes were combined, and the volume was adjusted to 50 µL by evaporation and dilution.

The 800-mer GFP (theoretically 5.12 nmol, corresponding to ~20 mg glass beads) and 1,728-mer Φ29 DNA polymerase (theoretically 33.28 nmol, corresponding to ~130 mg glass beads) genes synthesized on glass beads were cleaved and deprotected under the same conditions described above except that only a portion of glass beads was subjected to the procedure for the case of GFP gene.

Oligo purification using catching-by-polymerization (CBP): The purification of the 800-mer GFP gene synthesized on glass wool is used for the description. ***Polymerization***: To the 50 µL solution of crude oligo containing tagged full-length oligo and failure oligos (Scheme 1) as well as other materials (theoretically 29.4 nmol oligo) in a 1.5 mL centrifuge tube was added 12 µL polymerization solution (*N,N*-dimethylacrylamide, 340.5 µL, 3.32 mmol; *N,N'*-methylenebis(acrylamide), 17 mg, 0.11 mmol; sodium acrylate, 3 mg, 0.032 mmol; water, 170.5 µL). After mixing, ammonium persulfate (0.23 M, 5 µL) and *N,N,N',N'*-tetramethylethylenediamine (TMEDA, 0.69 M, 5 µL) were added. The tube was closed, and the content was quickly mixed with a short vortex and spin. The polymerization reaction was allowed to proceed at rt for 1 h. ***Washing***: The gel (~100 µL in size) was transferred into a 50 mL centrifuge tube. NaOAc solution (20%, pH unadjusted, 20 mL) was added. The mixture was gently shaken at rt overnight. The gel was taken to another 50 mL centrifuge tube, and Et_3N solution (5%, 20 mL) was added. The tube was shaken at rt overnight. The gel was taken out, placed into a 2 mL centrifugal filter unit over the filter, and cut into small pieces using a spatula. The gel was washed with water (0.5 mL × 5). ***Cleavage***: To half

volume of the gel, minimum AcOH (80%, ~200 µL) that could cover the gel was added. The mixture was incubated at rt for 5 min with occasional shaking. The liquid and gel were separated by centrifugation. The liquid was diluted with water (800 µL) to minimize the possibility of oligo damage by acid. The treatment of the gel with acid was repeated two times. The gel was washed with water (0.3 mL × 3). The bottom of the filtering unit holding the gel was stopped with parafilm, and to the gel was added water (500 µL). The mixture was shaken at rt overnight. The solution and gel was separated by centrifugation, and the gel was washed with water (250 µL × 2). These gave the oligo solutions in five tubes (~1 mL each). The solutions were concentrated, combined and evaporated to dryness. The residue acid is suggested to be removed by precipitation of the oligo from NH_4OH solution by *n*BuOH[4] if the oligo needs to be stored for more than two days. The oligo (theoretically 14.7 nmol) was dissolved in water (10 µL). Quantification using Qubit 4 Fluorometer indicated that 13.7 µg (55.56 pmol) oligo was obtained. The overall yield for the 800-mer synthesis and purification was 0.38% (Entry 8, Table 1).

The 800-mer GFP and 1,728-mer Φ29 DNA polymerase genes synthesized on glass beads were purified using the same CBP procedure described above. For the 800-mer, all the crude oligo (theoretically 5.12 nmol) was subjected to the polymerization for CBP. Half of the polyacrylamide gel was subjected for cleavage. Quantification of the purified oligo (theoretically 2.56 nmol) with Qubit 4 Fluorometer indicated that 84 ng (0.34 pmol) oligo was obtained. The overall yield for the 800-mer synthesis and purification was 0.013% (Entry 8, Table 1). For the 1,728-mer, all the crude oligo (theoretically 33.28 nmol) was subjected to the polymerization. Half of the gel was subjected to cleavage. Quantification of the purified oligo (theoretically 16.64 nmol) with Qubit 4 Fluorometer indicated that 1.416 µg (2.64 pmol) oligo was obtained. The overall yield for the 1,728-mer synthesis and purification was 0.016 % (Entry 10, Table 1).

PCR amplification: The 800-mer GFP gene synthesized on glass wool is used for the description. The mixture of CBP-purified oligo (~30 ng as quantified with Qubit 4 Fluorometer), Thermo Scientific™ Phusion™ High–Fidelity DNA Polymerase (0.4 U), forward and reverse primers (**p1a-b**, 0.5 µM each), HF buffer (1×), dNTPs (0.2 mM each), and nuclease-free water (20 µL total mixture volume, concentrations were final) was subjected to the following PCR cycles: 98 °C for 30 sec for initial denaturing; 98 °C for 7 sec, 58 °C for 15 sec, and 72 °C for 25 sec for 32 cycles; and 72 °C for 7 min. The PCR product was analyzed with agarose gel (1%) electrophoresis (GelRed® staining). Gel images are given in Figure 1.

The same PCR conditions were applied to the 800-mer GFP gene synthesized on glass beads. For the 1,728-mer Φ29 DNA polymerase gene, the PCR was conducted similarly except for the following modifications: primers were **p2a-b**; template quantity was ~7 ng; and the PCR cycles were modified so that the annealing step was 60.6 °C for 10 seconds and the following elongation step was 72 °C for 30 sec. Gel images of the PCR products are given in Figure 1.

Cloning and sequencing: The 800-mer GFP gene synthesized on glass wool is used for the description. The PCR product (3 µL out of 20 µL) was ligated into the pCR™4Blunt-TOPO™ vector following manufacturer's protocol. The resulting recombinant DNA was then transformed into One Shot Mach1 T1 Phage-Resistant Chemically Competent *E. coli* following manufacturer's protocol. The transformed cells (50 µL) were spread over kanamycin containing agar plates, which was incubated in a 37 °C incubator overnight. A portion of selected colonies was harvested using a sterile pipette tip and transferred into 50 µL of lysis buffer (1% triton X-100, 20 mM Tris, pH 8.0, 2 mM EDTA, pH 8.0). The lysis solutions were then heated at 95 °C for 10 min. Colony PCR reactions were set up using the recipe: DreamTaq PCR Mater Mix (1×), 2 µL colony lysis solution, primers **p1a-p1b** (0.5 µM each), and water to 20 µL (all concentrations are final). The samples were then subjected to the following PCR conditions: 95 °C for 2 min for initial denaturing; 95 °C for 30 sec for denaturing, 57 °C for 30 sec for annealing, and 72 °C for

extension for 1 min for 32 cycles; 72 °C for extension for 7 min. The PCR products were analyzed with electrophoresis on a 1% agarose gel (Figures 2A-C). Plasmid DNA from additional portions of the colonies that showed expected band in the colony PCR analysis were sent to MCLAB for Sanger sequencing. Both forward (**p4a**) and reverse (**p4b**) reads were obtained, which were assembled into a single contig using the Cap3 program (https://doua.prabi.fr/software/cap3). Several colonies that were unable to generate contigs due to poor sequencing quality were not included here. The contigs that were generated successfully were aligned to the reference sequence using the BLAST alignment software to trim off vector sequence and were then aligned with the reference sequence using CLUSTAL Omega. The data are included in the supporting information.

The cloning and sequencing procedures for the 800-mer GFP gene synthesized on glass beads were exactly the same. Gel images of colony PCR products are given in Figures 2D-F. For the 1,728-mer Φ29 DNA polymerase gene synthesized on glass beads, the following adjustments were applied. For colony PCR, the primers **p3a**-**b**, which covered a 600-mer region within the 1,728-mer gene, were used with an annealing temperature of 57.5 °C. Gel image of PCR products is given in Figure 2G. For Sanger sequencing and data analysis, four sequencing reads were generated for each plasmid, which was from the colonies giving bands in Figure 2G, using primers **p3a-b** and **p4a-b**. All four reads were used to generate the contig. Detailed sequence data are provided in the supporting information. The results are summarized in Table 2. Colony PCR was also carried out using primers **p2a-b**, which covered the entire 1,728-mer gene. The annealing temperature was 55.3 °C. The gel images of the PCR products are given in Figures 2H-I.

Table S1. Primers used in this study.

Primer	Sequence	Purpose
**p1a**	GCGAATTAATACGACTCACT	Primer for 800-mer GFP gene amplification
**p1b**	AAACCCCTCCGTTTTAGAGA	Primer for 800-mer GFP gene amplification
**p2a**	ATGAAGCATATGCCGAGAAA	Primer for 1,728-mer Φ29 DNA polymerase gene amplification
**p2b**	TTATTTGATTGTGAATGTGTCATCA	Primer for 1,728-mer Φ29 DNA polymerase gene amplification
**p3a**	ATGACAGCAGGCAGTGACA	Primer for 1,728-mer Φ29 DNA polymerase gene sequencing
**p3b**	GCTAGTTGCTTGATCGCTCC	Primer for 1,728-mer Φ29 DNA polymerase gene sequencing
**p4a**	GTAAAACGACGGCCAG	Primer for 800-mer GFP gene sequencing [M13 forward (-20)]
**p4b**	CAGGAAACAGCTATGAC	Primer for 800-mer GFP gene sequencing [M13 reverse]

Loading Calculation for Glass Wool and Glass Beads

Glass wool

Assumptions: mass, 1 g; density, d g/mL; diameter, 2r µm; radius, r µm; length, L µm

Volume of 1 g of solid glass: 1 g ÷ d g/cm^3 = $\frac{1}{d}$ cm^3 = $\frac{1}{d}\times10^{12}$ µm^3

Volume of 1 glass fiber: $r^2\pi L$ µm^3

Number of glass fiber: ($\frac{1}{d}\times10^{12}$ µm^3) ÷ ($r^2\pi L$ µm^3) = $\frac{10^{12}}{dr^2\pi L}$

Surface area of one glass fiber: $(2r^2\pi) + (2r\pi L) \approx 2r\pi L$ µm^2 (L is ~1,000 times of r)

Total surface area of 1 g glass wool: [2rπL μm^2] × $\frac{10^{12}}{dr^2\pi L}$ = $\frac{2}{dr}$ × 10^{12} μm^2 = $\frac{2}{dr}$ × 10^{18} nm^2

Area needed for one DNA molecule on surface: 3.2 molecules/nm^2 according to reference.[5]

Number of molecules: [$\frac{2}{dr}$ × 10^{18} nm^2] × [3.2 molecule/nm^2] = $\frac{6.4}{dr}$ × 10^{18} molecules

Mole of molecules: 10.403 × 10^{17} molecules = [$\frac{6.4}{dr}$ × 10^{18}] ÷ [6.02 × 10^{23}] mole = $\frac{1.063}{dr}$ × 10^{-5} mol = $\frac{10.63}{dr}$ μmol (μmol/g, where the units of d and r are g/mL and μm, respectively)

Example: when d = 2.2 g/mL and r = 4 μm, loading = 10.63 ÷ (2.2 × 4) = 1.208 (μmol/g)

Glass beads

Assumptions: mass, 1 g; density, d g/mL; diameter, 2r μm

Volume of 1 g of solid glass: 1 g ÷ d g/cm^3 = = $\frac{1}{d}$ cm^3 = $\frac{1}{d}$ ×10^{12} μm^3

Volume of one glass bead: $\frac{4}{3}$r^{3}π μm^3

Number of glass beads: ($\frac{1}{d}$ ×10^{12} μm^3) ÷ [$\frac{4}{3}$r^{3}π μm^3] = $\frac{3}{4dr^3\pi}$ × 10^{12}

Surface area of one glass bead: 4r^{2}π μm^2

Total surface area of 1 g glass bead: [4r^{2}π μm^2] × [$\frac{3}{4dr^3\pi}$ × 10^{12}] = $\frac{3}{dr}$ × 10^{12} μm^2 = $\frac{3}{dr}$ × 10^{18} nm^2

Area needed for one DNA molecule on surface: 3.2 molecules/nm^2 according to reference.[5]

Number of molecules: [$\frac{3}{dr}$ × 10^{18} nm^2] × [3.2 molecule/nm^2] = $\frac{9.6}{dr}$ × 10^{18} molecules

Mole of molecules: $\frac{9.6}{dr}$ × 10^{18} molecules = [$\frac{9.6}{dr}$ × 10^{18}] ÷ [6.02 × 10^{23}] mole = = $\frac{1.59}{dr}$ × 10^{-5} mol = $\frac{15.9}{dr}$ μmol (μmol/g, where the units of d and r are g/mL and μm, respectively).

Example: when d = 2.2 g/mL and r = 29 μm, loading = 15.9 ÷ (2.2 × 29) = 0.249 (μmol/g)

References

1. S. Q. Wang, D. D. Lu, L. Li and Y. Ding. (2021) reagent for direct condensation of oligonucleotides, its preparation and application. Patent number, cn101870717b.
2. Z. Kupihár, Z. Timár, Z. Darula, D. J. Dellinger and M. H. Caruthers. An electrospray mass spectrometric method for accurate mass determination of highly acid-sensitive phosphoramidites. *Rapid Commun. Mass Spec.*, 2008, **22**, 533-540. doi: 10.1002/rcm.3394
3. A. P. Guzaev and R. T. Pon. Attachment of nucleosides and other linkers to solid-phase supports for oligonucleotide synthesis. *Curr. Protoc. Nucleic Acid Chem.*, 2013, Chapter 3, 3 2 1-3 2 23. doi: 10.1002/0471142700.nc0302s52

4. S. Y. Fang, S. Fueangfung, X. Lin, X. A. Zhang, W. P. Mai, L. R. Bi and S. A. Green. Synthetic oligodeoxynucleotide purification by polymerization of failure sequences. *Chem. Commun.*, 2011, **47**, 1345-1347. doi: 10.1039/c0cc04374e
5. H. Sugimura, T. Moriguchi, M. Kanda, Y. Sonobayashi, H. M. Nishimura, T. Ichii, K. Murase and S. Kazama. Molecular packing density of a self-assembled monolayer formed from n-(2-aminoethyl)-3-aminopropyltriethoxysilane by a vapor phase process. *Chem. Commun.*, 2011, **47**, 8841-8843. doi: 10.1039/c1cc12541a

Sequencing results for the 800-mer GFP gene synthesized on glass wool (GW). "800mer_Ref" is the reference sequence. The numbers in the names of the sequences correspond to the lane numbers in Figures 3A-C.

```
800mer_Ref      AAACCCCTCCGTTTTAGAGAGGGGTTATGCTAGTTATTTGTAGAGCTCATCCATGCCATG    60
GW-1            AAACCCCTCCGTTTTAGAGAGGGGTTATGCTAGTTATTTGTAGAGCTCATCCATGCCATG    60
GW-2            AAACCCCTCCGTTTTAGAGAGGGGTTATGCTAGTTATTTGTAGAGCTCATCCATGCCATG    60
GW-3            AAACCCCTCCGTTTTAGAGAGGGGTTATGCTAGTTATTTGTAGAGCTCATCCATGCCATG    60
GW-4            AAACCCCTCCGTTTTAGAGAGGGGTTATGCTAGTTATTTGTAGAGCTCATCCATGCCATG    60
GW-5            AAACCCCTCCGTTTTAGAGAGGGGTTATGCTAGTTATTTGTAGAGCTCATCCATGCCATG    60
GW-6            AAACCCCTCCGTTTTAGAGAGGGGTTATGCTAGTTATTTGTAGAGCTCATCCATGCCATG    60
GW-8            AAACCCCTCCGTTTTAGAGAGGGGTTATGCTAGTTATTTGTAGAGCTCATCCATGCCATG    60
GW-9            AAACCCCTCCGTTTTAGAGAGGGGTTATGCTAGTTATTTGTAGAGCTCATCCATGCCATG    60
GW-11           AAACCCCTCCGTTTTAGAGAGGGGTTATGCTAGTTATTTGTAGAGCTCATCCATGCCATG    60
GW-12           AAACCCCTCCGTTTTAGAGAGGGGTTATGCTAGTTATTTGTAGAGCTCATCCATGCCATG    60
GW-13           AAACCCCTCCGTTTTAGAGAGGGGTTATGCTAGTTATTTGTAGAGCTCATCCATGCCATG    60
GW-14           AAACCCCTCCGTTTTAGAGAGGGGTTATGCTAGTTATTTGTAGAGCTCATCCATGCCATG    60
GW-15           AAACCCCTCCGTTTTAGAGAGGGGTTATGCTAGTCATTTGTAGAGCTCATCCATGCCATG    60
GW-16           AAACCCCTCCGTTTTAGAGAGGGGTTATGCTAGTTATTTGTAGAGCTCATCCATGCCATG    60
GW-17           AAACCCCTCCGTTTTAGAGAGGGGTTATGCTAGTTATTTGTAGAGCTCATCCATGCCATG    60
GW-18           AAACCCCTCCGTTTTAGAGAGGGGTTATGCTAGTTATTTGTAGAGCTCATCCATGCCATG    60
GW-20           AAACCCCTCCGTTTTAGAGAGGGGTTATGCTAGTTATTTGTAGAGCTCATCCATGCCATG    60
GW-21           AAACCCCTCCGTTTTAGAGAGGGGTTATGCTAGTTATTTGTAGAGCTCATCCATGCCATG    60
GW-23           AAACCCCTCCGTTTTAGAGAGGGGTTATGCTAGTTATTTGTAGAGCTCATCCATGCCATG    60
GW-24           AAACCCCTCCGTTTTAGAGAGGGGTTATGCTAGTTATTTGTAGAGCTCATCCATGCCATG    60
GW-25           AAACCCCTCCGTTTTAGAGAGGGGTTATGCTAGTTATTTGTAGAGCTCATCCATGCCATG    60
GW-26           AAACCCCTCCGTTTTAGAGAGGGGTTATGCTAGTTATTTGTAGAGCTCATCCATGCCATG    60
GW-27           AAACCCCTCCGTTTTAGAGAGGGGTTATGCTAGTTATTTGTAGAGCTCATCCATGCCATG    60
GW-28           AAACCCCTCCGTTTTAGAGAGGGGTTATGCTAGTTATTTGTAGAGCTCATCCATGCCATG    60
GW-29           AAACCCCTCCGTTTTAGAGAGGGGTTATGCTAGTTATTTGTAGAGCTCATCCATGCCATG    60
GW-30           AAACCCCTCCGTTTTAGAGAGGGGTTATGCTAGTTATTTGTAGAGCTCATCCATGCCATG    60
GW-32           AAACCCCTCCGTTTTAGAGAGGGGTTATGCTAGTTATTTGTAGAGCTCATCCATGCCATG    60
GW-33           AAACCCCTCCGTTTTAGAGAGGGGTTATGCTAGTTATTTGTAGAGCTCATCCATGCCATG    60
GW-34           AAACCCCTCCGTTTTAGAGAGGGGTTATGCTAGTTATTTGTAGAGCTCATCCATGCCATG    60
GW-35           AAACCCCTCCGTTTTAGAGAGGGGTTATGCTAGTTATTTGTAGAGCTCATCCATGCCATG    60
GW-36           AAACCCCTCCGTTTTAGAGAGGGGTTATGCTAGTTATTTGTAGAGCTCATCCATGCCATG    60
GW-38           AAACCCCTCCGTTTTAGAGAGGGGTTATGCTAGTTATTTGTAGAGCTCATCCATGCCATG    60
GW-39           AAACCCCTCCGTTTTAGAGAGGGGTTATGCTAGTTATTTGTAGAGCTCATCCATGCCATG    60
GW-40           AAACCCCTCCGTTTTAGAGAGGGGTTATGCTAGTTATTTGTAGAGCTCATCCATGCCATG    60
GW-42           AAACCCCTCCGTTTTAGAGAGGGGTTATGCTAGTTATTTGTAGAGCTCATCCATGCCATG    60
GW-45           AAACCCCTCCGTTTTAGAGAGGGGTTATGCTAGTTATTTGTAGAGCTCATCCATGCCATG    60
GW-48           AAACCCCTCCGTTTTAGAGAGGGGTTATGCTAGTTATTTGTAGAGCTCATCCATGCCATG    60
                ********************************** *************************

800mer_Ref      TGTAATCCCAGCAGCAGTTACAAACTCAAGAAGGACCATGTGGTCACGCTTTTCGTTGGG    120
GW-1            TGTAATCCCAGCAGCAGTTACAAACTCAAGAAGGACCATGTGGTCACGCTTTTCGTTGGG    120
GW-2            TGTAATCCCAGCAGCAGTTACAAACTCAAGAAGGACCATGTGGTCACGCTTTTCGTTGGG    120
GW-3            TGTAATCCCAGCAGCAGTTACAAACTCAAGAAGGACCATGTGGTCACGCTTTTCGTTGGG    120
GW-4            TGTAATCCCAGCAGCAGTTACAAACTCAAGAAGGACCATGTGGTCACGCTTTTCGTTGGG    120
```

```
GW-5          TGTAATCCCAGCAGCAGTTACAAACTCAAGAAGGACCATGTGGTCACGCTTTTCGTTGGG   120
GW-6          TGTAATCCCAGCAGCAGTTACAAACTCAAGAAGGACCATGTGGTCACGCTTTTCGTTGGG   120
GW-8          TGTAATCCCAGCAGCAGTTACAAACTCAAGAAGGACCATGTGGTCACGCTTTTCGTTGGG   120
GW-9          TGTAATCCCAGCAGCAGTTACAAACTCAAGAAGGACCATGTGGTCACGCTTTTCGTTGGG   120
GW-11         TGTAATCCCAGCAGCAGTTACAAACTCAAGAAGGACCATGTGGTCACGCTTTTCGTTGGG   120
GW-12         TGTAATCCCAGCAGCAGTTACAAACTCAAGAAGGACCATGTGGTCACGCTTTTCGTTGGG   120
GW-13         TGTAATCCCAGCAGCAGTTACAAACTCAAGAAGGACCATGTGGTCACGCTTTTCGTTGGG   120
GW-14         TGTAATCCCAGCAGCAGTTACAAACTCAAGAAGGACCATGTGGTCACGCTTTTCGTTGGG   120
GW-15         TGTAATCCCAGCAGCAGTTACAAACTCAAGAAGGACCATGTGGTCACGCTTTTCGTTGGG   120
GW-16         TGTAATCCCAGCAGCAGTTACAAACTCAAGAAGGACCATGTGGTCACGCTTTTCGTTGGG   120
GW-17         TGTAATCCCAGCAGCAGTTACAAACTCAAGAAGGACCATGTGGTCACGCTTTTCGTTGGG   120
GW-18         TGTAATCCCAGCAGCAGTTACAAACTCAAGAAGGACCATGTGGTCACGCTTTTCGTTGGG   120
GW-20         TGTAATCCCAGCAGCAGTTACAAACTCAAGAAGGACCATGTGGTCACGCTTTTCGTTGGG   120
GW-21         TGTAATCCCAGCAGCAGTTACAAACTCAAGAAGGACCATGTGGTCACGCTTTTCGTTGGG   120
GW-23         TGTAATCCCAGCAGCAGTTACAAACTCAAGAAGGTCCATGTGGTCACGCTTTTCGTTGGG   120
GW-24         TGTAATCCCAGCAGCAGTTACAAACTCAAGAAGGACCATGTGGTCACGCTTTTCGTTGGG   120
GW-25         TGTAATCCCAGCAGCAGTTACAAACTCAAGAAGGACCATGTGGTCACGCTTTTCGTTGGG   120
GW-26         TGTAATCCCAGCAGCAGTTACAAACTCAAGAAGGACCATGTGGTCACGCTTTTCGTTGGG   120
GW-27         TGTAATCCCAGCAGCAGTTACAAACTCAAGAAGGACCATGTGGTCACGCTTTTCGTTGGG   120
GW-28         TGTAATCCCAGCAGCAGTTACAAACTCAAGAAGGACCATGTGGTCACGCTTTTCGTTGGG   120
GW-29         TGTAATCCCAGCAGCAGTTACAAACTCAAGAAGGACCATGTGGTCACGCTTTTCGTTGGG   120
GW-30         TGTAATCCCAGCAGCAGTTACAAACTCAAGAAGGACCATGTGGTCACGCTTTTCGTTGGG   120
GW-32         TGTAATCCCAGCAGCAGTTACAAACTCAAGAAGGACCATGTGGTCACGCTTTTCGTTGGG   120
GW-33         TGTAATCCCAGCAGCAGTTACAAACTCAAGAAGGACCATGTGGTCACGCTTTTCGTTGGG   120
GW-34         TGTAATCCCAGCAGCAGTTACAAACTCAAGAAGGACCATGTGGTCACGCTTTTCGTTGGG   120
GW-35         TGTAATCCCAGCAGCAGTTACAAACTCAAGAAGGACCATGTGGTCACGCTTTTCGTTGGG   120
GW-36         TGTAATCCCAGCAGCAGTTACAAACTCAAGAAGGACCATGTGGTCACGCTTTTCGTTGGG   120
GW-38         TGTAATCCCAGCAGCAGTTACAAACTCAAGAAGGACCATGTGGTCACGCTTTTCGTTGGG   120
GW-39         TGTAATCCCAGCAGCAGTTACAAACTCAAGAAGGACCATGTGGTCACGCTTTTCGTTGGG   120
GW-40         TGTAATCCCAGCAGCAGTTACAAACTCAAGAAGGACCATGTGGTCACGCTTTTCGTTGGG   120
GW-42         TGTAATCCCAGCAGCAGTTACAAACTCAAGAAGGACCATGTGGTCACGCTTTTCGTTGGG   120
GW-45         TGTAATCCCAGCAGCAGTTACAAACTCAAGAAGGACCATGTGGTCACGCTTTTCGTTGGG   120
GW-48         TGTAATCCCAGCAGCAGTTACAAACTCAAGAAGGACCATGTGGTCACGCTTTTCGTTGGG   120
              ********************************** *************************

800mer_Ref    ATCTTTCGAAAGGGCAGATTGTGTCGACAGGTAATGGTTGTCTGGTAAAAGGACAGGGCC   180
GW-1          ATCTTTCGAAAGGGCAGATTGTGTCGACAGGTAATGGTTGTCTGGTAAAAGGACAGGGCC   180
GW-2          ATCTTTCGAAAGGGCAGATTGTGTCGACAGGTAATGGTTGTCTGGTAAAAGGACAGGGCC   180
GW-3          ATCTTTCGAAAGGGCAGATTGTGTCGACAGGTAATGGTTGTCTGGTAAAAGGACAGGGCC   180
GW-4          ATCTTTCGAAAGGGCAGATTGTGTCGACAGGTAATGGTTGTCTGGTAAAAGGACAGGGCC   180
GW-5          ATCTTTCGAAAGGGCAGATTGTGTCGACAGGTAATGGTTGTCTGGTAAAAGGACAGGGCC   180
GW-6          ATCTTTCGAAAGGGCAGATTGTGTCGACAGGTAATGGTTGTCTGGTAAAAGGACAGGGCC   180
GW-8          ATCTTTCGAAAGGGCAGATTGTGTCGACAGGTAATGGTTGTCTGGTAAAAGGACAGGGCC   180
GW-9          ATCTTTCGAAAGGGCAGATTGTGTCGACAGGTAATGGTTGTCTGGTAAAAGGACAGGGCC   180
GW-11         ATCTTTCGAAAGGGCAGATTGTGTCGACAGGTAATGGTTGTCTGGTAAAAGGACAGGGCC   180
GW-12         ATCTTTCGAAAGGGCAGATTGTGTCGACAGGTAATGGTTGTCTGGTAAAAGGACAGGGCC   180
GW-13         ATCTTTCGAAAGGGCAGATTGTGTCGACAGGTAATGGTTGTCTGGTAAAAGGACAGGGCC   180
GW-14         ATCTTTCGAAAGGGCAGATTGTGTCGACAGGTAATGGTTGTCTGGTAAAAGGACAGGGCC   180
GW-15         ATCTTTCGAAAGGGCAGATTGTGTCGACAGGTAATGGTTGTCTGGTAAAAGGACAGGGCC   180
GW-16         ATCTTTCGAAAGGGCAGATTGTGTCGACAGGTAATGGTTGTCTGGTAAAAGGACAGGGCC   180
```

```
GW-17          ATCTTTCGAAAGGGCAGATTGTGTCGACAGGTAATGGTTGTCTGGTAAAAGGACAGGGCC    180
GW-18          ATCTTTCGAAAGGGCAGATTGTGTCGACAGGTAATGGTTGTCTGGTAAAAGGACAGGGCC    180
GW-20          ATCTTTCGAAAGGGCAGATTGTGTCGACAGGTAATGGTTGTCTGGTAAAAGGACAGGGCC    180
GW-21          ATCTTTCGAAAGGGCAGATTGTGTCGACAGGTAATGGTTGTCTGGTAAAAGGACAGGGCC    180
GW-23          ATCTTTCGAAAGGGCAGATTGTGTCGACAGGTAATGGTTGTCTGGTAAAAGGACAGGGCC    180
GW-24          ATCTTTCGAAAGGGCAGATTGTGTCGACAGGTAATGGTTGTCTGGTAAAAGGACAGGGCC    180
GW-25          ATCTTTCGAAAGGGCAGATTGTGTCGACAGGTAATGGTTGTCTGGTAAAAGGACAGGGCC    180
GW-26          ATCTTTCGAAAGGGCAGATTGTGTCGACAGGTAATGGTTGTCTGGTAAAAGGACAGGGCC    180
GW-27          ATCTTTCGAAAGGGCAGATTGTGTCGACAGGTAATGGTTGTCTGGTAAAAGGACAGGGCC    180
GW-28          ATCTTTCGAAAGGGCAGATTGTGTCGACAGGTAATGGTTGTCTGGTAAAAGGACAGGGCC    180
GW-29          ATCTTTCGAAAGGGCAGATTGTGTCGACAGGTAATGGTTGTCTGGTAAAAGGACAGGGCC    180
GW-30          ATCTTTCGAAAGGGCAGATTGTGTCGACAGGTAATGGTTGTCTGGTAAAAGGACAGGGCC    180
GW-32          ATCTTTCGAAAGGGCAGATTGTGTCGACAGGTAATGGTTGTCTGGTAAAAGGACAGGGCC    180
GW-33          ATCTTTCGAAAGGGCAGATTGTGTCGACAGGTAATGGTTGTCTGGTAAAAGGACAGGGCC    180
GW-34          ATCTTTCGAAAGGGCAGATTGTGTCGACAGGTAATGGTTGTCTGGTAAAAGGACAGGGCC    180
GW-35          ATCTTTCGAAAGGGCAGATTGTGTCGACAGGTAATGGTTGTCTGGTAAAAGGACAGGGCC    180
GW-36          ATCTTTCGAAAGGGCAGATTGTGTCGACAGGTAATGGTTGTCTGGTAAAAGGACAGGGCC    180
GW-38          ATCTTTCGAAAGGGCAGATTGTGTCGACAGGTAATGGTTGTCTGGTAAAAGGACAGGGCC    180
GW-39          ATCTTTCGAAAGGGCAGATTGTGTCGACAGGTAATGGTTGTCTGGTAAAAGGACAGGGCC    180
GW-40          ATCTTTCGAAAGGGCAGATTGTGTCGACAGGTAATGGTTGTCTGGTAAAAGGACAGGGCC    180
GW-42          ATCTTTCGAAAGGGCAGATTGTGTCGACAGGTAATGGTTGTCTGGTAAAAGGACAGGGCC    180
GW-45          ATCTTTCGAAAGGGCAGATTGTGTCGACAGGTAATGGTTGTCTGGTAAAAGGACAGGGCC    180
GW-48          ATCTTTCGAAAGGGCAGATTGTGTCGACAGGTAATGGTTGTCTGGTAAAAGGACAGGGCC    180
               ************************************************************

800mer_Ref     ATCGCCAATTGGAGTATTTTGTTGATAATGGTCTGCTAGTTGAACGGATCCATCTTCAAT    240
GW-1           ATCGCCAATTGGAGTATTTTGTTGATAATGGTCTGCTAGTTGAACGGATCCATCTTCAAT    240
GW-2           ATCGCCAATTGGAGTATTTTGTTGATAATGGTCTGCTAGTTGAACGGATCCATCTTCAAT    240
GW-3           ATCGCCAATTGGAGTATTTTGTTGATAATGGTCTGCTAGTTGAACGGATCCATCTTCAAT    240
GW-4           ATCGCCAATTGGAGTATTTTGTTGATAATGGTCTGCTAGTTGAACGGATCCATCTTCAAT    240
GW-5           ATCGCCAATTGGAGTATTTTGTTGATAATGGTCTGCTAGTTGAACGGATCCATCTTCAAT    240
GW-6           ATCGCCAATTGGAGTATTTTGTTGATAATGGTCTGCTAGTTGAACGGATCCATCTTCAAT    240
GW-8           ATCGCCAATTGGAGTATTTTGTTGATAATGGTCTGCTAGTTGAACGGATCCATCTTCAAT    240
GW-9           ATCGCCAATTGGAGTATTTTGTTGATAATGGTCTGCTAGTTGAACGGATCCATCTTCAAT    240
GW-11          ATCGCCAATTGGAGTATTTTGTTGATAATGGTCTGCTAGTTGAACGGATCCATCTTCAAT    240
GW-12          ATCGCCAATTGGAGTATTTTGTTGATAATGGTCTGCTAGTTGAACGGATCCATCTTCAAT    240
GW-13          ATCGCCAATTGGAGTATTTTGTTGATAATGGTCTGCTAGTTGAACGGATCCATCTTCAAT    240
GW-14          ATCGCCAATTGGAGTATTTTGTTGATAATGGTCTGCTAGTTGAACGGATCCATCTTCAAT    240
GW-15          ATCGCCAATTGGAGTATTTTGTTGATAATGGTCTGCTAGTTGAACGGATCCATCTTCAAT    240
GW-16          ATCGCCAATTGGAGTATTTTGTTGATAATGGTCTGCTAGTTGAACGGATCCATCTTCAAT    240
GW-17          ATCGCCAATTGGAGTATTTTGTTGATAATGGTCTGCTAGTTGAACGGATCCATCTTCAAT    240
GW-18          ATCGCCAATTGGAGTATTTTGTTGATAATGGTCTGCTAGTTGAACGGATCCATCTTCAAT    240
GW-20          ATCGCCAATTGGAGTATTTTGTTGATAATGGTCTGCTAGTTGAACGGATCCATCTTCAAT    240
GW-21          ATCGCCAATTGGAGTATTTTGTTGATAATGGTCTGCTAGTTGAACGGATCCATCTTCAAT    240
GW-23          ATCGCCAATTGGAGTATTTTGTTGATAATGGTCTGCTAGTTGAACGGATCCATCTTCAAT    240
GW-24          ATCGCCAATTGGAGTATTTTGTTGATAATGGTCTGCTAGTTGAACGGATCCATCTTCAAT    240
GW-25          ATCGCCAATTGGAGTATTTTGTTGATAATGGTCTGCTAGTTGAACGGATCCATCTTCAAT    240
GW-26          ATCGCCAATTGGAGTATTTTGTTGATAATGGTCTGCTAGTTGAACGGATCCATCTTCAAT    240
GW-27          ATCGCCAATTGGAGTATTTTGTTGATAATGGTCTGCTAGTTGAACGGATCCATCTTCAAT    240
GW-28          ATCGCCAATTGGAGTATTTTGTTGATAATGGTCTGCTAGTTGAACGGATCCATCTTCAAT    240
```

```
GW-29           ATCGCCAATTGGAGTATTTTGTTGATAATGGTCTGCTAGTTGAACGGATCCATCTTCAAT    240
GW-30           ATCGCCAATTGGAGTATTTTGTTGATAATGGTCTGCTAGTTGAACGGATCCATCTTCAAT    240
GW-32           ATCGCCAATTGGAGTATTTTGTTGATAATGGTCTGCTAGTTGAACGGATCCATCTTCAAT    240
GW-33           ATCGCCAATTGGAGTATTTTGTTGATAATGGTCTGCTAGTTGAACGGATCCATCTTCAAT    240
GW-34           ATCGCCAATTGGAGTATTTTGTTGATAATGGTCTGCTAGTTGAACGGATCCATCTTCAAT    240
GW-35           ATCGCCAATTGGAGTATTTTGTTGATAATGGTCTGCTAGTTGAACGGATCCATCTTCAAT    240
GW-36           ATCGCCAATTGGAGTATTTTGTTGATAATGGTCTGCTAGTTGAACGGATCCATCTTCAAT    240
GW-38           ATCGCCAATTGGAGTATTTTGTTGATAATGGTCTGCTAGTTGAACGGATCCATCTTCAAT    240
GW-39           ATCGCCAATTGGAGTATTTTGTTGATAATGGTCTGCTAGTTGAACGGATCCATCTTCAAT    240
GW-40           ATCGCCAATTGGAGTATTTTGTTGATAATGGTCTGCTAGTTGAACGGATCCATCTTCAAT    240
GW-42           ATCGCCAATTGGAGTATTTTGTTGATAATGGTCTGCTAGTTGAACGGATCCATCTTCAAT    240
GW-45           ATCGCCAATTGGAGTATTTTGTTGATAATGGTCTGCTAGTTGAACGGATCCATCTTCAAT    240
GW-48           ATCGCCAATTGGAGTATTTTGTTGATAATGGTCTGCTAGTTGAACGGATCCATCTTCAAT    240
                ************************************************************

800mer_Ref      GTTGTGGCGAATTTTGAAGTTAGCTTTGATTCCATTCTTTTGTTTGTCTGCCGTGATGTA    300
GW-1            GTTGTGGCGAATTTTGAAGTTAGCTTTGATTCCATTCTTTTGTTTGTCTGCCGTGATGTA    300
GW-2            GTTGTGGCGAATTTTGAAGTTAGCTTTGATTCCATTCTTTTGTTTGTCTGCCGTGATGTA    300
GW-3            GTTGTGGCGAATTTTGAAGTTAGCTTTGATTCCATTCTTTTGTTTGTCTGCCGTGATGTA    300
GW-4            GTTGTGGCGAATTTTGAAGTTAGCTTTGATTCCATTCTTTTGTTTGTCTGCCGTGATGTA    300
GW-5            GTTGTGGCGAATTTTGAAGTTAGCTTTGATTCCATTCTTTTGTTTGTCTGCCGTGATGTA    300
GW-6            GTTGTGGCGAATTTTGAAGTTAGCTTTGATTCCATTCTTTTGTTTGTCTGCCGTGATGTA    300
GW-8            GTTGTGGCGAATTTTGAAGTTAGCTTTGATTCCATTCTTTTGTTTGTCTGCCGTGATGTA    300
GW-9            GTTGTGGCGAATTTTGAAGTTAGCTTTGATTCCATTCTTTTGTTTGTCTGCCGTGATGTA    300
GW-11           GTTGTGGCGAATTTTGAAGTTAGCTTTGATTCCATTCTTTTGTTTGTCTGCCGTGATGTA    300
GW-12           GTTGTGGCGAATTTTGAAGTTAGCTTTGATTCCATTCTTTTGTTTGTCTGCCGTGATGTA    300
GW-13           GTTGTGGCGAATTTTGAAGTTAGCTTTGATTCCATTCTTTTGTTTGTCTGCCGTGATGTA    300
GW-14           GTTGTGGCGAATTTTGAAGTTAGCTTTGATTCCATTCTTTTGTTTGTCTGCCGTGATGTA    300
GW-15           GTTGTGGCGAATTTTGAAGTTAGCTTTGATTCCATTCTTTTGTTTGTCTGCCGTGATGTA    300
GW-16           GTTGTGGCGAATTTTGAAGTTAGCTTTGATTCCATTCTTTTGTTTGTCTGCCGTGATGTA    300
GW-17           GTTGTGGCGAATTTTGAAGTTAGCTTTGATTCCATTCTTTTGTTTGTCTGCCGTGATGTA    300
GW-18           GTTGTGGCGAATTTTGAAGTTAGCTTTGATTCCATTCTTTTGTTTGTCTGCCGTGATGTA    300
GW-20           GTTGTGGCGAATTTTGAAGTTAGCTTTGATTCCATTCTTTTGTTTGTCTGCCGTGATGTA    300
GW-21           GTTGTGGCGAATTTTGAAGTTAGCTTTGATTCCATTCTTTTGTTTGTCTGCCGTGATGTA    300
GW-23           GTTGTGGCGAATTTTGAAGTTAGCTTTGATTCCATTCTTTTGTTTGTCTGCCGTGATGTA    300
GW-24           GTTGTGGCGAATTTTGAAGTTAGCTTTGATTCCATTCTTTTGTTTGTCTGCCGTGATGTA    300
GW-25           GTTGTGGCGAATTTTGAAGTTAGCTTTGATTCCATTCTTTTGTTTGTCTGCCGTGATGTA    300
GW-26           GTTGTGGCGAATTTTGAAGTTAGCTTTGATTCCATTCTTTTGTTTGTCTGCCGTGATGTA    300
GW-27           GTTGTGGCGAATTTTGAAGTTAGCTTTGATTCCATTCTTTTGTTTGTCTGCCGTGATGTA    300
GW-28           GTTGTGGCGAATTTTGAAGTTAGCTTTGATTCCATTCTTTTGTTTGTCTGCCGTGATGTA    300
GW-29           GTTGTGGCGAATTTTGAAGTTAGCTTTGATTCCATTCTTTTGTTTGTCTGCCGTGATGTA    300
GW-30           GTTGTGGCGAATTTTGAAGTTAGCTTTGATTCCATTCTTTTGTTTGTCTGCCGTGATGTA    300
GW-32           GTTGTGGCGAATTTTGAAGTTAGCTTTGATTCCATTCTTTTGTTTGTCTGCCGTGATGTA    300
GW-33           GTTGTGGCGAATTTTGAAGTTAGCTTTGATTCCATTCTTTTGTTTGTCTGCCGTGATGTA    300
GW-34           GTTGTGGCGAATTTTGAAGTTAGCTTTGATTCCATTCTTTTGTTTGTCTGCCGTGATGTA    300
GW-35           GTTGTGGCGAATTTTGAAGTTAGCTTTGATTCCATTCTTTTGTTTGTCTGCCGTGATGTA    300
GW-36           GTTGTGGCGAATTTTGAAGTTAGCTTTGATTCCATTCTTTTGTTTGTCTGCCGTGATGTA    300
GW-38           GTTGTGGCGAATTTTGAAGTTAGCTTTGATTCCATTCTTTTGTTTGTCTGCCGTGATGTA    300
GW-39           GTTGTGGCGAATTTTGAAGTTAGCTTTGATTCCATTCTTTTGTTTGTCTGCCGTGATGTA    300
GW-40           GTTGTGGCGAATTTTGAAGTTAGCTTTGATTCCATTCTTTTGTTTGTCTGCCGTGATGTA    300
```

```
GW-42           GTTGTGGCGAATTTTGAAGTTAGCTTTGATTCCATTCTTTTGTTTGTCTGCCGTGATGTA    300
GW-45           GTTGTGGCGAATTTTGAAGTTAGCTTTGATTCCATTCTTTTGTTTGTCTGCCGTGATGTA    300
GW-48           GTTGTGGCGAATTTTGAAGTTAGCTTTGATTCCATTCTTTTGTTTGTCTGCCGTGATGTA    300
                ************************************************************

800mer_Ref      TACATTGTGTGAGTTATAGTTGTACTCGAGTTTGTGTCCGAGAATGTTTCCATCTTCTTT    360
GW-1            TACATTGTGTGAGTTATAGTTGTACTCGAGTTTGTGTCCGAGAATGTTTCCATCTTCTTT    360
GW-2            TACATTGTGTGAGTTATAGTTGTACTCGAGTTTGTGTCCGAGAATGTTTCCATCTTCTTT    360
GW-3            TACATTGTGTGAGTTATAGTTGTACTCGAGTTTGTGTCCGAGAATGTTTCCATCTTCTTT    360
GW-4            TA-ATTGTGTGAGTTATAGTTGTACTCGAGTTTGTGTCCGAGAATGTTTCCATCTTCTTT    359
GW-5            TACATTGTGTGAGTTATAGTTGTACTCGAGTTTGTGTCCGAGAATGTTTCCATCTTCTTT    360
GW-6            TACATTGTGTGAGTTATAGTTGTACTCGAGTTTGTGTCCGAGAATGTTTCCATCTTCTTT    360
GW-8            TACATTGTGTGAGTTATAGTTGTACTCGAGTTTGTGTCCGAGAATGTTTCCATCTTCTTT    360
GW-9            TACATTGTGTGAGTTATAGTTGTACTCGAGTTTGTGTCCGAGAATGTTTCCATCTTCTTT    360
GW-11           TACATTGTGTGAGTTATAGTTGTACTCGAGTTTGTGTCCGAGAATGTTTCCATCTTCTTT    360
GW-12           TACATTGTGTGAGTTATAGTTGTACTCGAGTTTGTGTCCGAGAATGTTTCCATCTTCTTT    360
GW-13           TACATTGTGTGAGTTATAGTTGTACTCGAGTTTGTGTCCGAGAATGTTTCCATCTTCTTT    360
GW-14           TACATTGTGTGAGTTATAGTTGTACTCGAGTTTGTGTCCGAGAATGTTTCCATCTTCTTT    360
GW-15           TACATTGTGTGAGTTATAGTTGTACTCGAGTTTGTGTCCGAGAATGTTTCCATCTTCTTT    360
GW-16           TACATTGTGTGAGTTATAGTTGTACTCGAGTTTGTGTCCGAGAATGTTTCCATCTTCTTT    360
GW-17           TACATTGTGTGAGTTATAGTTGTACTCGAGTTTGTGTCCGAGAATGTTTCCATCTTCTTT    360
GW-18           TACATTGTGTGAGTTATAGTTGTACTCGAGTTTGTGTCCGAGAATGTTTCCATCTTCTTT    360
GW-20           TACATTGTGTGAGTTATAGTTGTACTCGAGTTTGTGTCCGAGAATGTTTCCATCTTCTTT    360
GW-21           TACATTGTGTGAGTTATAGTTGTACTCGAGTTTGTGTCCGAGAATGTTTCCATCTTCTTT    360
GW-23           TACATTGTGTGAGTTATAGTTGTACTCGAGTTTGTGTCCGAGAATGTTTCCATCTTCTTT    360
GW-24           TACATTGTGTGAGTTATAGTTGTACTCGAGTTTGTGTCCGAGAATGTTTCCATCTTCTTT    360
GW-25           TACATTGTGTGAGTTATAGTTGTACTCGAGTTTGTGTCCGAGAATGTTTCCATCTTCTTT    360
GW-26           TACATTGTGTGAGTTATAGTTGTACTCGAGTTTGTGTCCGAGAATGTTTCCATCTTCTTT    360
GW-27           TACATTGTGTGAGTTATAGTTGTACTCGAGTTTGTGTCCGAGAATGTTTCCATCTTCTTT    360
GW-28           TACATTGTGTGAGTTATAGTTGTACTCGAGTTTGTGTCCGAGAATGTTTCCATCTTCTTT    360
GW-29           TACATTGTGTGAGTTATAGTTGTACTCGAGTTTGTGTCCGAGAATGTTTCCATCTTCTTT    360
GW-30           TACATTGTGTGAGTTATAGTTGTACTCGAGTTTGTGTCCGAGAATGTTTCCATCTTCTTT    360
GW-32           TACATTGTGTGAGTTATAGTTGTACTCGAGTTTGTGTCCGAGAATGTTTCCATCTTCTTT    360
GW-33           TACATTGTGTGAGTTATAGTTGTACTCGAGTTTGTGTCCGAGAATGTTTCCATCTTCTTT    360
GW-34           TACATTGTGTGAGTTATAGTTGTACTCGAGTTTGTGTCCGAGAATGTTTCCATCTTCTTT    360
GW-35           TACATTGTGTGAGTTATAGTTGTACTCGAGTTTGTGTCCGAGAATGTTTCCATCTTCTTT    360
GW-36           TACATTGTGTGAGTTATAGTTGTACTTGAGTTTGTGTCCGAGAATGTTTCCAT-------    353
GW-38           TACATTGTGTGAGTTATAGTTGTACTCGAGTTTGTGTCCGAGAATGTTTCCATCTTCTTT    360
GW-39           TACATTGTGTGAGTTATAGTTGTACTCGAGTTTGTGTCCGAGAATGTTTCCATCTTCTTT    360
GW-40           TACATTGTGTGAGTTATAGTTGTACTCGAGTTTGTGTCCGAGAATGTTTCCATCTTCTTT    360
GW-42           TACATTGTGTGAGTTATAGTTGTACTCGAGTTTGTGTCCGAGAATGTTTCCATCTTCTTT    360
GW-45           TACATTGTGTGAGTTATAGTTGTACTCGAGTTTGTGTCCGAGAATGTTTCCATCTTCTTT    360
GW-48           TACATTGTGTGAGTTATAGTTGTACTCGAGTTTGTGTCCGAGAATGTTTCCATCTTCTTT    360
                ** *********************** **************************

800mer_Ref      AAAATCAATACCTTTTAACTCGATACGATTAACAAGGGTATCACCTTCAAACTTGACTTC    420
GW-1            AAAATCAATACCTTTTAACTCGATACGATTAACAAGGGTATCACCTTCAAACTTGACTTC    420
GW-2            AAAATCAATACCTTTTAACTCGATACGATTAACAAGGGTATCACCTTCAAACTTGACTTC    420
GW-3            AAAATCAATACCTTTTAACTCGATACGATTAACAAGGGTATCACCTTCAAACTTGACTTC    420
GW-4            AAAATCAATACCTTTTAACTCGATACGATTAACAAGGGTATCACCTTCAAACTTGACTTC    419
```

```
GW-5            AAAATCAATACCTTTTAACTCGATACGATTAACAAGGGTATCACCTTCAAACTTGACTTC    420
GW-6            AAAATCAATACCTTTTAACTCGATACGATTAACAAGGGTATCACCTTCAAACTTGACTTC    420
GW-8            AAAATCAATACCTTTTAACTCGATACGATTAACAAGGGTATCACCTTCAAACTTGACTTC    420
GW-9            AAAATCAATACCTTTTAACTCGATACGATTAACAAGGGTATCACCTTCAAACTTGACTTC    420
GW-11           AAAATCAATACCTTTTAACTCGATACGATTAACAAGGGTATCACCTTCAAACTTGACTTC    420
GW-12           AAAATCAATACCTTTTAACTCGATACGATTAACAAGGGTATCACCTTCAAACTTGACTTC    420
GW-13           AAAATCAATACCTTTTAACTCGATACGATTAACAAGGGTATCACCTTCAAACTTGACTTC    420
GW-14           AAAATCAATACCTTTTAACTCGATACGATTAACAAGGGTATCACCTTCAAACTTGACTTC    420
GW-15           AAAATCAATACCTTTTAACTCGATACGATTAACAAGGGTATCACCTTCAAACTTGACTTC    420
GW-16           AAAATCAATACCTTTTAACTCGATACGATTAACAAGGGTATCACCTTCAAACTTGACTTC    420
GW-17           AAAATCAATACCTTTTAACTCGATACGATTAACAAGGGTATCACCTTCAAACTTGACTTC    420
GW-18           AAAATCAATACCTTTTAACTCGATACGATTAACAAGGGTATCACCTTCAAACTTGACTTC    420
GW-20           AAAATCAATACCTTTTAACTCGATACGATTAACAAGGGTATCACCTTCAAACTTGACTTC    420
GW-21           AAAATCAATACCTTTTAACTCGATACGATTAACAAGGGTATCACCTTCAAACTTGACTTC    420
GW-23           AAAATCAATACCTTTTAACTCGATACGATTAACAAGGGTATCACCTTCAAACTTGACTTC    420
GW-24           AAAATCAATACCTTTTAACTCGATACGATTAACAAGGGTATCACCTTCAAACTTGACTTC    420
GW-25           AAAATCAATACCTTTTAACTCGATACGATTAACAAGGGTATCACCTTCAAACTTGACTTC    420
GW-26           AAAATCAATACCTTTTAACTCGATACGATTAACAAGGGTATCACCTTCAAACTTGACTTC    420
GW-27           AAAATCAATACCTTTTAACTCGATACGATTAACAAGGGTATCACCTTCAAACTTGACTTC    420
GW-28           AAAATCAATACCTTTTAACTCGATACGATTAACAAGGGTATCACCTTCAAACTTGACTTC    420
GW-29           AAAATCAATACCTTTTAACTCGATACGATTAACAAGGGTATCACCTTCAAACTTGACTTC    420
GW-30           AAAATCAATACCTTTTAACTCGATACGATTAACAAGGGTATCACCTTCAAACTTGACTTC    420
GW-32           AAAATCAATACCTTTTAACTCGATACGATTAACAAGGGTATCACCTTCAAACTTGACTTC    420
GW-33           AAAATCAATACCTTTTAACTCGATACGATTAACAAGGGTATCACCTTCAAACTTGACTTC    420
GW-34           AAAATCAATACCTTTTAACTCGATACGATTAACAAGGGTATCACCTTCAAACTTGACTTC    420
GW-35           AAAATCAATACCTTTTAACTCGATACGATTAACAAGGGTATCACCTTCAAACTTGACTTC    420
GW-36           ---ATCAATACCTTTTAACTCGATACGATTAACAAGGGTATCACCTTCAAACTTGACTTC    410
GW-38           AAAATCAATACCTTTTAACTCGATACGATTAACAAGGGTATCACCTTCAAACTTGACTTC    420
GW-39           AAAATCAATACCTTTTAACTCGATACGATTAACAAGGGTATCACCTTCAAACTTGACTTC    420
GW-40           AAAATCAATACCTTTTAACTCGATACGATTAACAAGGGTATCACCTTCAAACTTGACTTC    420
GW-42           AAAATCAATACCTTTTAACTCGATACGATTAACAAGGGTATCACCTTCAAACTTGACTTC    420
GW-45           AAAATCAATACCTTTTAACTCGATACGATTAACAAGGGTATCACCTTCAAACTTGACTTC    420
GW-48           AAAATCAATACCTTTTAACTCGATACGATTAACAAGGGTATCACCTTCAAACTTGACTTC    420
                   *********************************************************

800mer_Ref      AGCACGCGTCTTGTAGTTCCCGTCATCTTTGAAAGATATAGTGCGTTCCTGTACATAACC    480
GW-1            AGCACGCGTCTTGTAGTTCCCGTCATCTTTGAAAGATATAGTGCGTTCCTGTACATAACC    480
GW-2            AGCACGCGTCTTGTAGTTCCCGTCATCTTTGAAAGATATAGTGCGTTCCTGTACATAACC    480
GW-3            AGCACGCGTCTTGTAGTTCCCGTCATCTTTGAAAGATATAGTGCGTTCCTGTACATAACC    480
GW-4            AGCACGCGTCTTGTAGTTCCCGTCATCTTTGAAAGATATAGTGCGTTCCTGTACATAACC    479
GW-5            AGCACGCGTCTTGTAGTTCCCGTCATCTTTGAAAGATATAGTGCGTTCCTGTACATAACC    480
GW-6            AGCACGCGTCTTGTAGTTCCCGTCATCTTTGAAAGATATAGTGCGTTCCTGTACATAACC    480
GW-8            AGCACGCGTCTTGTAGTTCCCGTCATCTTTGAAAGATATAGTGCGTTCCTGTACATAACC    480
GW-9            AGCACGCGTCTTGTAGTTCCCGTCATCTTTGAAAGATATAGTGCGTTCCTGTACATAACC    480
GW-11           AGCACGCGTCTTGTAGTTCCCGTCATCTTTGAAAGATATAGTGCGTTCCTGTACATAACC    480
GW-12           AGCACGCGTCTTGTAGTTCCCGTCATCTTTGAAAGATATAGTGCGTTCCTGTACATAACC    480
GW-13           AGCACGCGTCTTGTAGTTCCCGTCATCTTTGAAAGATATAGTGCGTTCCTGTACATAACC    480
GW-14           AGCACGCGTCTTGTAGTTCCCGTCATCTTTGAAAGATATAGTGCGTTCCTGTACATAACC    480
GW-15           AGCACGCGTCTTGTAGTTCCCGTCATCTTTGAAAGATATAGTGCGTTCCTGTACATAACC    480
GW-16           AGCACGCGTCTTGTAGTTCCCGTCATCTTTGAAAGATATAGTGCGTTCCTGTACATAACC    480
```

```
GW-17         AGCACGCGTCTTGTAGTTCCCGTCATCTTTGAAAGATATAGTGCGTTCCTGTACATAACC    480
GW-18         AGCACGCGTCTTGTAGTTCCCGTCATCTTTGAAAGATATAGTGCGTTCCTGTACATAACC    480
GW-20         AGCACGCGTCTTGTAGTTCCCGTCATCTTTGAAAGATATAGTGCGTTCCTGTACATAACC    480
GW-21         AGCACGCGTCTTGTAGTTCCCGTCATCTTTGAAAGATATAGTGCGTTCCTGTACATAACC    480
GW-23         AGCACGCGTCTTGTAGTTCCCGTCATCTTTGAAAGATATAGTGCGTTCCTGTACATAACC    480
GW-24         AGCACGCGTCTTGTAGTTCCCGTCATCTTTGAAAGATATAGTGCGTTCCTGTACATAACC    480
GW-25         AGCACGCGTCTTGTAGTTCCCGTCATCTTTGAAAGATATAGTGCGTTCCTGTACATAACC    480
GW-26         AGCACGCGTCTTGTAGTTCCCGTCATCTTTGAAAGATATAGTGCGTTCCTGTACATAACC    480
GW-27         AGCACGCGTCTTGTAGTTCCCGTCATCTTTGAAAGATATAGTGCGTTCCTGTACATAACC    480
GW-28         AGCACGCGTCTTGTAGTTCCCGTCATCTTTGAAAGATATAGTGCGTTCCTGTACATAACC    480
GW-29         AGCACGCGTCTTGTAGTTCCCGTCATCTTTGAAAGATATAGTGCGTTCCTGTACATAACC    480
GW-30         AGCACGCGTCTTGTAGTTCCCGTCATCTTTGAAAGATATAGTGCGTTCCTGTACATAACC    480
GW-32         AGCACGCGTCTTGTAGTTCCCGTCATCTTTGAAAGATATAGTGCGTTCCTGTACATAACC    480
GW-33         AGCACGCGTCTTGTAGTTCCCGTCATCTTTGAAAGATATAGTGCGTTCCTGTACATAACC    480
GW-34         AGCACGCGTCTTGTAGTTCCCGTCATCTTTGAAAGATATAGTGCGTTCCTGTACATAACC    480
GW-35         AGCACGCGTCTTGTAGTTCCCGTCATCTTTGAAAGATATAGTGCGTTCCTGTACATAACC    480
GW-36         AGCACGCGTCTTGTAGTTCCCGTCATCTTTGAAAGATATAGTGCGTTCCTGTACATAACC    470
GW-38         AGCACGCGTCTTGTAGTTCCCGTCATCTTTGAAAGATATAGTGCGTTCCTGTACATAACC    480
GW-39         AGCACGCGTCTTGTAGTTCCCGTCATCTTTGAAAGATATAGTGCGTTCCTGTACATAACC    480
GW-40         AGCACGCGTCTTGTAGTTCCCGTCATCTTTGAAAGATATAGTGCGTTCCTGTACATAACC    480
GW-42         AGCACGCGTCTTGTAGTTCCCGTCATCTTTGAAAGATATAGTGCGTTCCTGTACATAACC    480
GW-45         AGCACGCGTCTTGTAGTTCCCGTCATCTTTGAAAGATATAGTGCGTTCCTGTACATAACC    480
GW-48         AGCACGCGTCTTGTAGTTCCCGTCATCTTTGAAAGATATAGTGCGTTCCTGTACATAACC    480
              ************************************************************

800mer_Ref    TTCGGGCATGGCACTCTTGAAAAAGTCATGCCGTTTCATATGATCCGGATAACGGGAAAA    540
GW-1          TTCGGGCATGGCACTCTTGAAAAAGTCATGCCGTTTCATATGATCCGGATAACGGGAAAA    540
GW-2          TTCGGGCATGGCACTCTTGAAAAAGTCATGCCGTTTCATATGATCCGGATAACGGGAAAA    540
GW-3          TTCGGGCATGGCACTCTTGAAAAAGTCATGCCGTTTCATATGATCCGGATAACGGGAAAA    540
GW-4          TTCGGGCATGGCACTCTTGAAAAAGTCATGCCGTTTCATATGATCCGGATAACGGGAAAA    539
GW-5          TTCGGGCATGGCACTCTTGAAAAAGTCATGCCGTTTCATATGATCCGGATAACGGGAAAA    540
GW-6          TTCGGGCATGGCACTCTTGAAAAAGTCATGCCGTTTCATATGATCCGGATAACGGGAAAA    540
GW-8          TTCGGGCATGGCACTCTTGAAAAAGTCATGCCGTTTCATATGATCCGGATAACGGGAAAA    540
GW-9          TTCGGGCATGGCACTCTTGAAAAAGTCATGCCGTTTCATATGATCCGGATAACGGGAAAA    540
GW-11         TTCGGGCATGGCACTCTTGAA-AAGTCATGCCGTTTCATATGATCCGGATAACGGGAAAA    539
GW-12         TTCGGGCATGGCACTCTTGAAAAAGTCATGCCGTTTCATATGATCCGGATAACGGGAAAA    540
GW-13         TTCGGGCATGGCACTCTTGAAAAAGTCATGCCGTTTCATATGATCCGGATAACGGGAAAA    540
GW-14         TTCGGGCATGGCACTCTTGAAAAAGTCATGCCGTTTCATATGATCCGGATAACGGGAAAA    540
GW-15         TTCGGGCATGGCACTCTTGAAAAAGTCATGCCGTTTCATATGATCCGGATAACGGGAAAA    540
GW-16         TTCGGGCATGGCACTCTTGAAAAAGTCATGCCGTTTCATATGATCCGGATAACGGGAAAA    540
GW-17         TTCGGGCATGGCACTCTTGAAAAAGTCATGCCGTTTCATATGATCCGGATAACGGGAAAA    540
GW-18         TTCGGGCATGGCACTCTTGAAAAAGTCATGCCGTTTCATATGATCCGGATAACGGGAAAA    540
GW-20         TTCGGGCATGGCACTCTTGAAAAAGTCATGCCGTTTCATATGATCCGGATAACGGGAAAA    540
GW-21         TTCGGGCATGGCACTCTTGAAAAAGTCATGCCGTTTCATATGATCCGGATAACGGGAAAA    540
GW-23         TTCGGGCATGGCACTCTTGAAAAAGTCATGCCGTTTCATATGATCCGGATAACGGGAAAA    540
GW-24         TTCGGGCATGGCACTCTTGAAAAAGTCATGCCGTTTCATATGATCCGGATAACGGGAAAA    540
GW-25         TTCGGGCATGGCACTCTTGAAAAAGTCATGCCGTTTCATATGATCCGGATAACGGGAAAA    540
GW-26         TTCGGGCATGGCACTCTTGAAAAAGTCATGCCGTTTCATATGATCCGGATAACGGGAAAA    540
GW-27         TTCGGGCATGGCACTCTTGAAAAAGTCATGCCGTTTCATATGATCCGGATAACGGGAAAA    540
GW-28         TTCGGGCATGGCACTCTTGAAAAAGTCATGCCGTTTCATATGATCCGGATAACGGGAAAA    540
```

```
GW-29           TTCGGGCATGGCACTCTTGAAAAAGTCATGCCGTTTCATATGATCCGGATAACGGGAAAA    540
GW-30           TTCGGGCATGGCACTCTTGAAAAAGTCATGCCGTTTCATATGATCCGGATAACGGGAAAA    540
GW-32           TTCGGGCATGGCACTCTTGAAAAAGTCATGCCGTTTCATATGATCCGGATAACGGGAAAA    540
GW-33           TTCGGGCATGGCACTCTTGAAAAAGTCATGCCGTTTCATATGATCCGGATAACGGGAAAA    540
GW-34           TTCGGGCATGGCACTCTTGAAAAAGTCATGCCGTTTCATATGATCCGGATAACGGGAAAA    540
GW-35           TTCGGGCATGGCACTCTTGAAAAAGTCATGCCGTTTCATATGATCCGGATAACGGGAAAA    540
GW-36           TTCGGGCATGGCACTCTTGAAAAAGTCATGCCGTTTCATATGATCCGGATAACGGGAAAA    530
GW-38           TTCGGGCATGGCACTCTTGAAAAAGTCATGCCGTTTCATATGATCCGGATAACGGGAAAA    540
GW-39           TTCGGGCATGGCACTCTTGAAAAAGTCATGCCGTTTCATATGATCCGGATAACGGGAAAA    540
GW-40           TTCGGGCATGGCACTCTTGAAAAAGTCATGCCGTTTCATATGATCCGGATAACGGGAAAA    540
GW-42           TTCGGGCATGGCACTCTTGAAAAAGTCATGCCGTTTCATATGATCCGGATAACGGGAAAA    540
GW-45           TTCGGGCATGGCACTCTTGAAAAAGTCATGCCGTTTCATATGATCCGGATAACGGGAAAA    540
GW-48           TTCGGGCATGGCACTCTTGAAAAAGTCATGCCGTTTCATATGATCCGGATAACGGGAAAA    540
                ******************** ***************************************

800mer_Ref      GCATTGAACACCATAAGAGAAAGTAGTGACAAGTGTTGGCCATGGAACAGGTAGTTTTCC    600
GW-1            GCATTGAACACCATAAGAGAAAGTAGTGACAAGTGTTGGCCATGGAACAGGTAGTTTTCC    600
GW-2            GCATTGAACACCATAAGAGAAAGTAGTGACAAGTGTTGGCCATGGAACAGGTAGTTTTCC    600
GW-3            GCATTGAACACCATAAGAGAAAGTAGTGACAAGTGTTGGCCATGGAACAGGTAGTTTTCC    600
GW-4            GCATTGAACACCATAAGAGAAAGTAGTGACAAGTGTTGGCCATGGAACAGGTAGTTTTCC    599
GW-5            GCATTGAACACCATAAGAGAAAGTAGTGACAAGTGTTGGCCATGGAACAGGTAGTTTTCC    600
GW-6            GCATTGAACACCATAAGAGAAAGTAGTGACAAGTGTTGGCCATGGAACAGGTAGTTTTCC    600
GW-8            GCATTGAACACCATAAGAGAAAGTAGTGACAAGTGTTGGCCATGGAACAGGTAGTTTTCC    600
GW-9            GCATTGAACACCATAAGAGAAAGTAGTGACAAGTGTTGGCCATGGAACAGGTAGTTTTCC    600
GW-11           GCATTGAACACCATAAGAGAAAGTAGTGACAAGTGTTGGCCATGGAACAGGTAGTTTTCC    599
GW-12           GCATTGAACACCATAAGAGAAAGTAGTGACAAGTGTTGGCCATGGAACAGGTAGTTTTCC    600
GW-13           GCATTGAACACCATAAGAGAAAGTAGTGACAAGTGTTGGCCATGGAACAGGTAGTTTTCC    600
GW-14           GCATTGAACACCATAAGAGAAAGTAGTGACAAGTGTTGGCCATGGAACAGGTAGTTTTCC    600
GW-15           GCATTGAACACCATAAGAGAAAGTAGTGACAAGTGTTGGCCATGGAACAGGTAGTTTTCC    600
GW-16           GCATTGAACACCATAAGAGAAAGTAGTGACAAGTGTTGGCCATGGAACAGGTAGTTTTCC    600
GW-17           GCATTGAACACCATAAGAGAAAGTAGTGACAAGTGTTGGCCATGGAACAGGTAGTTTTCC    600
GW-18           GCATTGAACACCATAAGAGAAAGTAGTGACAAGTGTTGGCCATGGAACAGGTAGTTTTCC    600
GW-20           GCATTGAACACCATAAGAGAAAGTAGTGACAAGTGTTGGCCATGGAACAGGTAGTTTTCC    600
GW-21           GCATTGAACACCATAAGAGAAAGTAGTGACAAGTGTTGGCCATGGAACAGGTAGTTTTCC    600
GW-23           GCATTGAACACCATAAGAGAAAGTAGTGACAAGTGTTGGCCATGGAACAGGTAGTTTTCC    600
GW-24           GCATTGAACACCATAAGAGAAAGTAGTGACAAGTGTTGGCCATGGAACAGGTAGTTTTCC    600
GW-25           GCATTGAACACCATAAGAGAAAGTAGTGACAAGTGTTGGCCATGGAACAGGTAGTTTTCC    600
GW-26           GCATTGAACACCATAAGAGAAAGTAGTGACAAGTGTTGGCCATGGAACAGGTAGTTTTCC    600
GW-27           GCATTGAACACCATAAGAGAAAGTAGTGACAAGTGTTGGCCATGGAACAGGTAGTTTTCC    600
GW-28           GCATTGAACACCATAAGAGAAAGTAGTGACAAGTGTTGGCCATGGAACAGGTAGTTTTCC    600
GW-29           GCATTGAACACCATAAGAGAAAGTAGTGACAAGTGTTGGCCATGGAACAGGTAGTTTTCC    600
GW-30           GCATTGAACACCATAAGAGAAAGTAGTGACAAGTGTTGGCCATGGAACAGGTAGTTTTCC    600
GW-32           GCATTGAACACCATAAGAGAAAGTAGTGACAAGTGTTGGCCATGGAACAGGTAGTTTTCC    600
GW-33           GCATTGAACACCATAAGAGAAAGTAGTGACAAGTGTTGGCCATGGAACAGGTAGTTTTCC    600
GW-34           GCATTGAACACCATAAGAGAAAGTAGTGACAAGTGTTGGCCATGGAACAGGTAGTTTTCC    600
GW-35           GCATTGAACACCATAAGAGAAAGTAGTGACAAGTGTTGGCCATGGAACAGGTAGTTTTCC    600
GW-36           GCATTGAACACCATAAGAGAAAGTAGTGACAAGTGTTGGCCATGGAACAGGTAGTTTTCC    590
GW-38           GCATTGAACACCATAAGAGAAAGTAGTGACAAGTGTTGGCCATGGAACAGGTAGTTTTCC    600
GW-39           GCATTGAACACCATAAGAGAAAGTAGTGACAAGTGTTGGCCATGGAACAGGTAGTTTTCC    600
GW-40           GCATTGAACACCATAAGAGAAAGTAGTGACAAGTGTTGGCCATGGAACAGGTAGTTTTCC    600
```

```
GW-42           GCATTGAACACCATAAGAGAAAGTAGTGACAAGTGTTGGCCATGGAACAGGTAGTTTTCC    600
GW-45           GCATTGAACACCATAAGAGAAAGTAGTGACAAGTGTTGGCCATGGAACAGGTAGTTTTCC    600
GW-48           GCATTGAACACCATAAGAGAAAGTAGTGACAAGTGTTGGCCATGGAACAGGTAGTTTTCC    600
                ************************************************************

800mer_Ref      AGTAGTGCAAATAAATTTAAGGGTAAGTTTTCCGTATGTTGCATCACCTTCACCCTCTCC    660
GW-1            AGTAGTGCAAATAAATTTAAGGGTAAGTTTTCCGTATGTTGCATCACCTTCACCCTCTCC    660
GW-2            AGTAGTGCAAATAAATTTAAGGGTAAGTTTTCCGTATGTTGCATCACCTTCACCCTCTCC    660
GW-3            AGTAGTGCAAATAAATTTAAGGGTAAGTTTTCCGTATGTTGCATCACCTTCACCCTCTCC    660
GW-4            AGTAGTGCAAATAAATTTAAGGGTAAGTTTTCCGTATGTTGCATCACCTTCACCCTCTCC    659
GW-5            AGTAGTGCAAATAAATTTAAGGGTAAGTTTTCCGTATGTTGCATCACCTTCACCCTCTCC    660
GW-6            AGTAGTGCAAATAAATTTAAGGGTAAGTTTTCCGTATGTTGCATCACCTTCACCCTCTCC    660
GW-8            AGTAGTGCAAATAAATTTAAGGGTAAGTTTTCCGTATGTTGCATCACCTTCACCCTCTCC    660
GW-9            AGTAGTGCAAATAAATTTAAGGGTAAGTTTTCCGTATGTTGCATCACCTTCACCCTCTCC    660
GW-11           AGTAGTGCAAATAAATTTAAGGGTAAGTTTTCCGTATGTTGCATCACCTTCACCCTCTCC    659
GW-12           AGTAGTGCAAATAAATTTAAGGGTAAGTTTTCCGTATGTTGCATCACCTTCACCCTCTCC    660
GW-13           AGTAGTGCAAATAAATTTAAGGGTAAGTTTTCCGTATGTTGCATCACCTTCACCCTCTCC    660
GW-14           AGTAGTGCAAATAAATTTAAGGGTAAGTTTTCCGTATGTTGCATCACCTTCACCCTCTCC    660
GW-15           AGTAGTGCAAATAAATTTAAGGGTAAGTTTTCCGTATGTTGCATCACCTTCACCCTCTCC    660
GW-16           AGTAGTGCAAATAAATTTAAGGGTAAGTTTTCCGTATGTTGCATCACCTTCACCCTCTCC    660
GW-17           AGTAGTGCAAATAAATTTAAGGGTAAGTTTTCCGTATGTTGCATCACCTTCACCCTCTCC    660
GW-18           AGTAGTGCAAATAAATTTAAGGGTAAGTTTTCCGTATGTTGCATCACCTTCACCCTCTCC    660
GW-20           AGTAGTGCAAATAAATTTAAGGGTAAGTTTTCCGTATGTTGCATCACCTTCACCCTCTCC    660
GW-21           AGTAGTGCAAATAAATTTAAGGGTAAGTTTTCCGTATGTTGCATCACCTTCACCCTCTCC    660
GW-23           AGTAGTGCAAATAAATTTAAGGGTAAGTTTTCCGTATGTTGCATCACCTTCACCCTCTCC    660
GW-24           AGTAGTGCAAATAAATTTAAGGGTAAGTTTTCCGTATGTTGCATCACCTTCACCCTCTCC    660
GW-25           AGTAGTGCAAATAAATTTAAGGGTAAGTTTTCCGTATGTTGCATCACCTTCACCCTCTCC    660
GW-26           AGTAGTGCAAATAAATTTAAGGGTAAGTTTTCCGTATGTTGCATCACCTTCACCCTCTCC    660
GW-27           AGTAGTGCAAATAAATTTAAGGGTAAGTTTTCCGTATGTTGCATCACCTTCACCCTCTCC    660
GW-28           AGTAGTGCAAATAAATTTAAGGGTAAGTTTTCCGTATGTTGCATCACCTTCACCCTCTCC    660
GW-29           AGTAGTGCAAATAAATTTAAGGGTAAGTTTTCCGTATGTTGCATCACCTTCACCCTCTCC    660
GW-30           AGTAGTGCAAATAAATTTAAGGGTAAGTTTTCCGTATGTTGCATCACCTTCACCCTCTCC    660
GW-32           AGTAGTGCAAATAAATTTAAGGGTAAGTTTTCCGTATGTTGCATCACCTTCACCCTCTCC    660
GW-33           AGTAGTGCAAATAAATTTAAGGGTAAGTTTTCCGTATGTTGCATCACCTTCACCCTCTCC    660
GW-34           AGTAGTGCAAATAAATTTAAGGGTAAGTTTTCCGTATGTTGCATCACCTTCACCCTCTCC    660
GW-35           AGTAGTGCAAATAAATTTAAGGGTAAGTTTTCCGTATGTTGCATCACCTTCACCCTCTCC    660
GW-36           AGTAGTGCAAATAAATTTAAGGGTAAGTTTTCCGTATGTTGCATCACCTTCACCCTCTCC    650
GW-38           AGTAGTGCAAATAAATTTAAGGGTAAGTTTTCCGTATGTTGCATCACCTTCACCCTCTCC    660
GW-39           AGTAGTGCAAATAAATTTAAGGGTAAGTTTTCCGTATGTTGCATCACCTTCACCCTCTCC    660
GW-40           AGTAGTGCAAATAAATTTAAGGGTAAGTTTTCCGTATGTTGCATCACCTTCACCCTCTCC    660
GW-42           AGTAGTGCAAATAAATTTAAGGGTAAGTTTTCCGTATGTTGCATCACCTTCACCCTCTCC    660
GW-45           AGTAGTGCAAATAAATTTAAGGGTAAGTTTTCCGTATGTTGCATCACCTTCACCCTCTCC    660
GW-48           AGTAGTGCAAATAAATTTAAGGGTAAGTTTTCCGTATGTTGCATCACCTTCACCCTCTCC    660
                ************************************************************

800mer_Ref      ACTGACAGAAAATTTGTGCCCATTAACATCACCATCTAATTCAACAAGAATTGGGACAAC    720
GW-1            ACTGACAGAAAATTTGTGCCCATTAACATCACCATCTAATTCAACAAGAATTGGGACAAC    720
GW-2            ACTGACAGAAAATTTGTGCCCATTAACATCACCATCTAATTCAACAAGAATTGGGACAAC    720
GW-3            ACTGACAGAAAATTTGTGCCCATTAACATCACCATCTAATTCAACAAGAATTGGGACAAC    720
GW-4            ACTGACAGAAAATTTGTGCCCATTAACATCACCATCTAATTCAACAAGAATTGGGACAAC    719
```

```
GW-5           ACTGACAGAAAATTTGTGCCCATTAACATCACCATCTAATTCAACAAGAATTGGGACAAC    720
GW-6           ACTGACAGAAAATTTGTGCCCATTAACATCACCATCTAATTCAACAAGAATTGGGACAAC    720
GW-8           ACTGACAGAAAATTTGTGCCCATTAACATCACCATCTAATTCAACAAGAATTGGGACAAC    720
GW-9           ACTGACAGAAAATTTGTGCCCATTAACATCACCATCTAATTCAACAAGAATTGGGACAAC    720
GW-11          ACTGACAGAAAATTTGTGCCCATTAACATCACCATCTAATTCAACAAGAATTGGGACAAC    719
GW-12          ACTGACAGAAAATTTGTGCCCATTAACATCACCATCTAATTCAACAAGAATTGGGACAAC    720
GW-13          ACTGACAGAAAATTTGTGCCCATTAACATCACCATCTAATTCAACAAGAATTGGGACAAC    720
GW-14          ACTGACAGAAAATTTGTGCCCATTAACATCACCATCTAATTCAACAAGAATTGGGACAAC    720
GW-15          ACTGACAGAAAATTTGTGCCCATTAACATCACCATCTAATTCAACAAGAATTGGGACAAC    720
GW-16          ACTGACAGAAAATTTGTGCCCATTAACATCACCATCTAATTCAACAAGAATTGGGACAAC    720
GW-17          ACTGACAGAAAATTTGTGCCCATTAACATCACCATCTAATTCAACAAGAATTGGGACAAC    720
GW-18          ACTGACAGAAAATTTGTGCCCATTAACATCACCATCTAATTCAACAAGAATTGGGACAAC    720
GW-20          ACTGACAGAAAATTTGTGCCCATTAACATCACCATCTAATTCAACAAGAATTGGGACAAC    720
GW-21          ACTGACAGAAAATTTGTGCCCATTAACATCACCATCTAATTCAACAAGAATTGGGACAAC    720
GW-23          ACTGACAGAAAATTTGTGCCCATTAACATCACCATCTAATTCAACAAGAATTGGGACAAC    720
GW-24          ACTGACAGAAAATTTGTGCCCATTAACATCACCATCTAATTCAACAAGAATTGGGACAAC    720
GW-25          ACTGACAGAAAATTTGTGCCCATTAACATCACCATCTAATTCAACAAGAATTGGGACAAC    720
GW-26          ACTGACAGAAAATTTGTGCCCATTAACATCACCATCTAATTCAACAAGAATTGGGACAAC    720
GW-27          ACTGACAGAAAATTTGTGCCCATTAACATCACCATCTAATTCAACAAGAATTGGGACAAC    720
GW-28          ACTGACAGAAAATTTGTGCCCATTAACATCACCATCTAATTCAACAAGAATTGGGACAAC    720
GW-29          ACTGACAGAAAATTTGTGCCCATTAACATCACCATCTAATTCAACAAGAATTGGGACAAC    720
GW-30          ACTGACAGAAAATTTGTGCCCATTAACATCACCATCTAATTCAACAAGAATTGGGACAAC    720
GW-32          ACTGACAGAAAATTTGTGCCCATTAACATCACCATCTAATTCAACAAGAATTGGGACAAC    720
GW-33          ACTGACAGAAAATTTGTGCCCATTAACATCACCATCTAATTCAACAAGAATTGGGACAAC    720
GW-34          ACTGACAGAAAATTTGTGCCCATTAACATCACCATCTAATTCAACAAGAATTGGGACAAC    720
GW-35          ACTGACAGAAAATTTGTGCCCATTAACATCACCATCTAATTCAACAAGAATTGGGACAAC    720
GW-36          ACTGACAGAAAATTTGTGCCCATTAACATCACCATCTAATTCAACAAGAATTGGGACAAC    710
GW-38          ACTGACAGAAAATTTGTGCCCATTAACATCACCATCTAATTCAACAAGAATTGGGACAAC    720
GW-39          ACTGACAGAAAATTTGTGCCCATTAACATCACCATCTAATTCAACAAGAATTGGGACAAC    720
GW-40          ACTGACAGAAAATTTGTGCCCATTAACATCACCATCTAATTCAACAAGAATTGGGACAAC    720
GW-42          ACTGACAGAAAATTTGTGCCCATTAACATCACCATCTAATTCAACAAGAATTGGGACAAC    720
GW-45          ACTGACAGAAAATTTGTGCCCATTAACATCACCATCTAATTCAACAAGAATTGGGACAAC    720
GW-48          ACTGACAGAAAATTTGTGCCCATTAACATCACCATCTAATTCAACAAGAATTGGGACAAC    720
               ************************************************************

800mer_Ref     TCCAGTGAAAAGTTCTTCTCCTTTACTCATATTTTTTCCTCCTTATACTTAAGCCCTATA    780
GW-1           TCCAGTGAAAAGTTCTTCTCCTTTACTCATATTTTTTCCTCCTTATACTTAAGCCCTATA    780
GW-2           TCCAGTGAAAAGTTCTTCTCCTTTACTCATATTTTTTCCTCCTTATACTTAAGCCCTATA    780
GW-3           TCCAGTGAAAAGTTCTTCTCCTTTACTCATATTTTTTCCTCCTTATACTTAAGCCCTATA    780
GW-4           TCCAGTGAAAAGTTCTTCTCCTTTACTCATATTTTTTCCTCCTTATACTTAAGCCCTATA    779
GW-5           TCCAGTGAAAAGTTCTTCTCCTTTACTCATATTTTTTCCTCCTTATACTTAAGCCCTATA    780
GW-6           TCCAGTGAAAAGTTCTTCTCCTTTACTCATATTTTTTCCTCCTTATACTTAAGCCCTATA    780
GW-8           TCCAGTGAAAAGTTCTTCTCCTTTACTCATATTTTTTCCTCCTTATACTTAAGCCCTATA    780
GW-9           TCCAGTGAAAAGTTCTTCTCCTTTACTCATATTTTTTCCTCCTTATACTTAAGCCCTATA    780
GW-11          TCCAGTGAAAAGTTCTTCTCCTTTACTCATATTTTTTCCTCCTTATACTTAAGCCCTATA    779
GW-12          TCCAGTGAAAAGTTCTTCTCCTTTACTCATATTTTTTCCTCCTTATACTTAAGCCCTATA    780
GW-13          TCCAGTGAAAAGTTCTTCTCCTTTACTCATATTTTTTCCTCCTTATACTTAAGCCCTATA    780
GW-14          TCCAGTGAAAAGTTCTTCTCCTTTACTCATATTTTTTCCTCCTTATACTTAAGCCCTATA    780
GW-15          TCCAGTGAAAAGTTCTTCTCCTTTACTCATATTTTTTCCTCCTTATACTTAAGCCCTATA    780
GW-16          TCCAGTGAAAAGTTCTTCTCCTTTACTCATATTTTTTCCTCCTTATACTTAAGCCCTATA    780
```

```
GW-17           TCCAGTGAAAAGTTCTTCTCCTTTACTCATATTTTTTCCTCCTTATACTTAAGCCCTATA    780
GW-18           TCCAGTGAAAAGTTCTTCTCCTTTACTCATATTTT-TCCTCCTTATACTTAAGCCCTATA    779
GW-20           TCCAGTGAAAAGTTCTTCTCCTTTACTCATATTTTTTCCTCCTTATACTTAAGCCCTATA    780
GW-21           TCCAGTGAAAAGTTCTTCTCCTTTACTCATATTTTTTCCTCCTTATACTTAAGCCCTATA    780
GW-23           TCCAGTGAAAAGTTCTTCTCCTTTACTCATATTTTTTCCTCCTTATACTTAAGCCCTATA    780
GW-24           TCCAGTGAAAAGTTCTTCTCCTTTACTCATATTTTTTCCTCCTTATACTTAAGCCCTATA    780
GW-25           TCCAGTGAAAAGTTCTTCTCCTTTACTCATATTTTTTCCTCCTTATACTTAAGCCCTATA    780
GW-26           TCCAGTGAAAAGTTCTTCTCCTTTACTCATATTTTTTCCTCCTTATACTTAAGCCCTATA    780
GW-27           TCCAGTGAAAAGTTCTTCTCCTTTACTCATATTTT-TCCTCCTTATACTTAAGCCCTATA    779
GW-28           TCCAGTGAAAAGTTCTTCTCCTTTACTCATATTTTTTCCTCCTTATACTTAAGCCCTATA    780
GW-29           TCCAGTGAAAAGTTCTTCTCCTTTACTCATATTTTTTCCTCCTTATACTTAAGCCCTATA    780
GW-30           TCCAGTGAAAAGTTCTTCTCCTTTACTCATATTTTTTCCTCCTTATACTTAAGCCCTATA    780
GW-32           TCCAGTGAAAAGTTCTTCTCCTTTACTCATATTTTTTCCTCCTTATACTTAAGCCCTATA    780
GW-33           TCCAGTGAAAAGTTCTTCTCCTTTACTCATATTTTTTCCTCCTTATACTTAAGCCCTATA    780
GW-34           TCCAGTGAAAAGTTCTTCTCCTTTACTCATATTTTTTCCTCCTTATACTTAAGCCCTATA    780
GW-35           TCCAGTGAAAAGTTCTTCTCCTTTACTCATATTTTTTCCTCCTTATACTTAAGCCCTATA    780
GW-36           TCCAGTGAAAAGTTCTTCTCCTTTACTCATATTTTTTCCTCCTTATACTTAAGCCCTATA    770
GW-38           TCCAGTGAAAAGTTCTTCTCCTTTACTCATATTTTTTCCTCCTTATACTTAAGCCCTATA    780
GW-39           TCCAGTGAAAAGTTCTTCTCCTTTACTCATATTTTTTCCTCCTTATACTTAAGCCCTATA    780
GW-40           TCCAGTGAAAAGTTCTTCTCCTTTACTCATATTTTTTCCTCCTTATACTTAAGCCCTATA    780
GW-42           TCCAGTGAAAAGTTCTTCTCCTTTACTCATATTTTTTCCTCCTTATACTTAAGCCCTATA    780
GW-45           TCCAGTGAAAAGTTCTTCTCCTTTACTCATATTTTTTCCTCCTTATACTTAAGCCCTATA    780
GW-48           TCCAGTGAAAAGTTCTTCTCCTTTACTCATATTTTTTCCTCCTTATACTTAAGCCCTATA    780
                *********************************** ************************

800mer_Ref      GTGAGTCGTATTAATTCGC     799
GW-1            GTGAGTCGTATTAATTCGC     799
GW-2            GTGAGTCGTATTAATTCGC     799
GW-3            GTGAGTCGTATTAATTCGC     799
GW-4            GTGAGTCGTATTAATTCGC     798
GW-5            GTGAGTCGTATTAATTCGC     799
GW-6            GTGAGTCGTATTAATTCGC     799
GW-8            GTGAGTCGTATTAATTCGC     799
GW-9            GTGAGTCGTATTAATTCGC     799
GW-11           GTGAGTCGTATTAATTCGC     798
GW-12           GTGAGTCGTATTAATTCGC     799
GW-13           GTGAGTCGTATTAATTCGC     799
GW-14           GTGAGTCGTATTAATTCGC     799
GW-15           GTGAGTCGTATTAATTCGC     799
GW-16           GTGAGTCGTATTAATTCGC     799
GW-17           GTGAGTCGTATTAATTCGC     799
GW-18           GTGAGTCGTATTAATTCGC     798
GW-20           GTGAGTCGTATTAATTCGC     799
GW-21           GTGAGTCGTATTAATTCGC     799
GW-23           GTGAGTCGTATTAATTCGC     799
GW-24           GTGAGTCGTATTAATTCGC     799
GW-25           GTGAGTCGTATTAATTCGC     799
GW-26           GTGAGTCGTATTAATTCGC     799
GW-27           GTGAGTCGTATTAATTCGC     798
GW-28           GTGAGTCGTATTAATTCGC     799
```

```
GW-29          GTGAGTCGTATTAATTCGC     799
GW-30          GTGAGTCGTATTAATTCGC     799
GW-32          GTGAGTCGTATTAATTCGC     799
GW-33          GTGAGTCGTATTAATTCGC     799
GW-34          GTGAGTCGTATTAATTCGC     799
GW-35          GTGAGTCGTATTAATTCGC     799
GW-36          GTGAGTCGTATTAATTCGC     789
GW-38          GTGAGTCGTATTAATTCGC     799
GW-39          GTGAGTCGTATTAATTCGC     799
GW-40          GTGAGTCGTATTAATTCGC     799
GW-42          GTGAGTCGTATTAATTCGC     799
GW-45          GTGAGTCGTATTAATTCGC     799
GW-48          GTGAGTCGTATTAATTCGC     799
               *******************
```

Sequencing results for the 800-mer GFP gene synthesized on glass beads (GB). "800mer_Ref" is the reference sequence. The numbers in the names of the sequences correspond to the lane numbers in Figures 3D-F.

```
800mer_Ref      AAACCCCTCCGTTTTAGAGAGGGGTTATGCTAGTTATTTGTAGAGCTCATCCATGCCATG    60
GB-1            AAACCCCTCCGTTTTAGAGAGGGGTTATGCTAGTTATTTGTAGAGCTCATCCATGCCATG    60
GB-2            AAACCCCTCCGTTTTAGAGAGGGGTTATGCTAGTTATTTGTAGAGCTCATCCATGCCATG    60
GB-3            AAACCCCTCCGTTTTAGAGAGGGGTTATGCTAGTTATTTGTAGAGCTCATCCATGCCATG    60
GB-4            AAACCCCTCCGTTTTAGAGAGGGGTTATGCTAGTTATTTGTAGAGCTCATCCATGCCATG    60
GB-5            AAACCCCTCCGTTTTAGAGAGGGGTTATGCTAGTTATTTGTAGAGCTCATCCATGCCATG    60
GB-7            AAACCCCTCCGTTTTAGAGAGGGGTTATGCTAGTTATTTGTAGAGCTCATCCATGCCATG    60
GB-10           AAACCCCTCCGTTTTAGAGAGGGGTTATGCTAGTTATTTGTAGAGCTCATCCATGCCATG    60
GB-12           AAACCCCTCCGTTTTAGAGAGGGGTTATGCTAGTTATTTGTAGAGCTCATCCATGCCATG    60
GB-15           AAACCCCTCCGTTTTAGAGAGGGGTTATGCTAGTTATTTGTAGAGCTCATCCATGCCATG    60
GB-16           AAACCCCTCCGTTTTAGAGAGGGGTTATGCTAGTTATTTGTAGAGCTCATCCATGCCATG    60
GB-17           AAACCCCTCCGTTTTAGAGAGGGGTTATGCTAGTTATTTGTAGAGCTCATCCATGCCATG    60
GB-18           AAACCCCTCCGTTTTAGAGAGGGGTTATGCTAGTTATTTGTAGAGCTCATCCATGCCATG    60
GB-19           AAACCCCTCCGTTTTAGAGAGGGGTTATGCTAGTTATTTGTAGAGCTCATCCATGCCATG    60
GB-20           AAACCCCTCCGTTTTAGAGAGGGGTTATGCTAGTTATTTGTAGAGCTCATCCATGCCATG    60
GB-21           AAACCCCTCCGTTTTAGAGAGGGGTTATGCTAGTTATTTGTAGAGCTCATCCATGCCATG    60
GB-22           AAACCCCTCCGTTTTAGAGAGGGGTTATGCTAGTTATTTGTAGAGCTCATCCATGCCATG    60
GB-23           AAACCCCTCCGTTTTAGAGAGGGGTTATGCTAGTTATTTGTAGAGCTCATCCATGCCATG    60
GB-24           AAACCCCTCCGTTTTAGAGAGGGGTTATGCTAGTTATTTGTAGAGCTCATCCATGCCATG    60
GB-25           AAACCCCTCCGTTTTAGAGAGGGGTTATGCTAGTTATTTGTAGAGCTCATCCATGCCATG    60
GB-27           AAACCCCTCCGTTTTAGAGAGGGGTTATGCTAGTTATTTGTAGAGCTCATCCATGCCATG    60
GB-29           AAACCCCTCCGTTTTAGAGAGGGGTTATGCTAGTTATTTGTAGAGCTCATCCATGCCATG    60
GB-30           AAACCCCTCCGTTTTAGAGAGGGGTTATGCTAGTTATTTGTAGAGCTCATCCATGCCATG    60
GB-31           AAACCCCTCCGTTTTAGAGAGGGGTTATGCTAGTTATTTGTAGAGCTCATCCATGCCATG    60
GB-33           AAACCCCTCCGTTTTAGAGAGGGGTTATGCTAGTTATTTGTAGAGCTCATCCATGCCATG    60
GB-34           AAACCCCTCCGTTTTAGAGAGGGGTTATGCTAGTTATTTGTAGAGCTCATCCATGCCATG    60
GB-36           AAACCCCTCCGTTTTAGAGAGGGGTTATGCTAGTTATTTGTAGAGCTCATCCATGCCATG    60
GB-38           AAACCCCTCCGTTTTAGAGAGGGGTTATGCTAGTTATTTGTAGAGCTCATCCATGCCATG    60
GB-40           AAACCCCTCCGTTTTAGAGAGGGGTTATGCTAGTTATTTGTAGAGCTCATCCATGCCATG    60
GB-41           AAACCCCTCCGTTTTAGAGAGGGGTTATGCTAGTTATTTGTAGAGCTCATCCATGCCATG    60
GB-42           AAACCCCTCCGTTTTAGAGAGGGGTTATGCTAGTTATTTGTAGAGCTCATCCATGCCATG    60
GB-43           AAACCCCTCCGTTTTAGAGAGGGGTTATGCTAGTTATTTGTAGAGCTCATCCATGCCATG    60
GB-44           AAACCCCTCCGTTTTAGAGAGGGGTTATGCTAGTTATTTGTAGAGCTCATCCATGCCATG    60
GB-45           AAACCCCTCCGTTTTAGAGAGGGGTTATGCTAGTTATTTGTAGAGCTCATCCATGCCATG    60
GB-46           AAACCCCTCCGTTTTAGAGAGGGGTTATGCTAGTTATTTGTAGAGCTCATCCATGCCATG    60
GB-47           AAACCCCTCCGTTTTAGAGAGGGGTTATGCTAGTTATTTGTAGAGCTCATCCATGCCATG    60
                ************************************************************

800mer_Ref      TGTAATCCCAGCAGCAGTTACAAACTCAAGAAGGACCATGTGGTCACGCTTTTCGTTGGG    120
GB-1            TGTAATCCCAGCAGCAGTTACAAACTCAAGAAGGACCATGTGGTCACGCTTTTCGTTGGG    120
GB-2            TGTAATCCCAGCAGCAGTTACAAACTCAAGAAGGACCATGTGGTCACGCTTTTCGTTGGG    120
GB-3            TGTAATCCCAGCAGCAGTTACAAACTCAAGAAGGACCATGTGGTCACGCTTTTCGTTGGG    120
GB-4            TGTAATCCCAGCAGCAGTTACAAACTCAAGAAGGACCATGTGGTCACGCTTTTCGTTGGG    120
GB-5            TGTAATCCCAGCAGCAGTTACAAACTCAAGAAGGACCATGTGGTCACGCTTTTCGTTGGG    120
GB-7            TGTAATCCCAGCAGCAGTTACAAACTCAAGAAGGACCATGTGGTCACGCTTTTCGTTGGG    120
```

```
GB-10          TGTAATCCCAGCAGCAGTTACAAACTCAAGAAGGACCATGTGGTCACGCTTTTCGTTGGG    120
GB-12          TGTAATCCCAGCAGCAGTTACAAACTCAAGAAGGACCATGTGGTCACGCTTTTCGTTGGG    120
GB-15          TGTAATCCCAGCAGCAGTTACAAACTCAAGAAGGACCATGTGGTCACGCTTTTCGTTGGG    120
GB-16          TGTAATCCCAGCAGCAGTTACAAACTCAAGAAGGACCATGTGGTCACGCTTTTCGTTGGG    120
GB-17          TGTAATCCCAGCAGCAGTTACAAACTCAAGAAGGACCATGTGGTCACGCTTTTCGTTGGG    120
GB-18          TGTAATCCCAGCAGCAGTTACAAACTCAAGAAGGACCATGTGGTCACGCTTTTCGTTGGG    120
GB-19          TGTAATCCCAGCAGCAGTTACAAACTCAAGAAGGACCATGTGGTCACGCTTTTCGTTGGG    120
GB-20          TGTAATCCCAGCAGCAGTTACAAACTCAAGAAGGACCATGTGGTCACGCTTTTCGTTGGG    120
GB-21          TGTAATCCCAGCAGCAGTTACAAACTCAAGAAGGACCATGTGGTCACGCTTTTCGTTGGG    120
GB-22          TGTAATCCCAGCAGCAGTTACAAACTCAAGAAGGACCATGTGGTCACGCTTTTCGTTGGG    120
GB-23          TGTAATCCCAGCAGCAGTTACAAACTCAAGAAGGACCATGTGGTCACGCTTTTCGTTGGG    120
GB-24          TGTAATCCCAGCAGCAGTTACAAACTCAAGAAGGACCATGTGGTCACGCTTTTCGTTGGG    120
GB-25          TGTAATCCCAGCAGCAGTTACAAACTCAAGAAGGACCATGTGGTCACGCTTTTCGTTGGG    120
GB-27          TGTAATCCCAGCAGCAGTTACAAACTCAAGAAGGACCATGTGGTCACGCTTTTCGTTGGG    120
GB-29          TGTAATCCCAGCAGCAGTTACAAACTCAAGAAGGACCATGTGGTCACGCTTTTCGTTGGG    120
GB-30          TGTAATCCCAGCAGCAGTTACAAACTCAAGAAGGACCATGTGGTCACGCTTTTCGTTGGG    120
GB-31          TGTAATCCCAGCAGCAGTTACAAACTCAAGAAGGACCATGTGGTCACGCTTTTCGTTGGG    120
GB-33          TGTAATCCCAGCAGCAGTTACAAACTCAAGAAGGACCATGTGGTCACGCTTTTCGTTGGG    120
GB-34          TGTAATCCCAGCAGCAGTTACAAACTCAAGAAGGACCATGTGGTCACGCTTTTCGTTGGG    120
GB-36          TGTAATCCCAGCAGCAGTTACAAACTCAAGAAGGACCATGTGGTCACGCTTTTCGTTGGG    120
GB-38          TGTAATCCCAGCAGCAGTTACAAACTCAAGAAGGACCATGTGGTCACGCTTTTCGTTGGG    120
GB-40          TGTAATCCCAGCAGCAGTTACAAACTCAAGAAGGACCATGTGGTCACGCTTTTCGTTGGG    120
GB-41          TGTAATCCCAGCAGCAGTTACAAACTCAAGAAGGACCATGTGGTCACGCTTTTCGTTGGG    120
GB-42          TGTAATCCCAGCAGCAGTTACAAACTCAAGAAGGACCATGTGGTCACGCTTTTCGTTGGG    120
GB-43          TGTAATCCCAGCAGCAGTTACAAACTCAAGAAGGACCATGTGGTCACGCTTTTCGTTGGG    120
GB-44          TGTAATCCCAGCAGCAGTTACAAACTCAAGAAGGACCATGTGGTCACGCTTTTCGTTGGG    120
GB-45          TGTAATCCCAGCAGCAGTTACAAACTCAAGAAGGACCATGTGGTCACGCTTTTCGTTGGG    120
GB-46          TGTAATCCCAGCAGCAGTTACAAACTCAAGAAGGACCATGTGGTCACGCTTTTCGTTGGG    120
GB-47          TGTAATCCCAGCAGCAGTTACAAACTCAAGAAGGACCATGTGGTCACGCTTTTCGTTGGG    120
               ************************************************************

800mer_Ref     ATCTTTCGAAAGGGCAGATTGTGTCGACAGGTAATGGTTGTCTGGTAAAAGGACAGGGCC    180
GB-1           ATCTTTCGAAAGGGCAGATTGTGTCGACAGGTAATGGTTGTCTGGTAAAAGGACAGGGCC    180
GB-2           ATCTTTCGAAAGGGCAGATTGTGTCGACAGGTAATGGTTGTCTGGTAAAAGGACAGGGCC    180
GB-3           ATCTTTCGAAAGGGCAGATTGTGTCGACAGGTAATGGTTGTCTGGTAAAAGGACAGGGCC    180
GB-4           ATCTTTCGAAAGGGCAGATTGTGTCGACAGGTAATGGTTGTCTGGTAAAAGGACAGGGCC    180
GB-5           ATCTTTCGAAAGGGCAGATTGTGTCGACAGGTAATGGTTGTCTGGTAAAAGGACAGGGCC    180
GB-7           ATCTTTCGAAAGGGCAGATTGTGTCGACAGGTAATGGTTGTCTGGTAAAAGGACAGGGCC    180
GB-10          ATCTTTCGAAAGGGCAGATTGTGTCGACAGGTAATGGTTGTCTGGTAAAAGGACAGGGCC    180
GB-12          ATCTTTCGAAAGGGCAGATTGTGTCGACAGGTAATGGTTGTCTGGTAAAAGGACAGGGCC    180
GB-15          ATCTTTCGAAAGGGCAGATTGTGTCGACAGGTAATGGTTGTCTGGTAAAAGGACAGGGCC    180
GB-16          ATCTTTCGAAAGGGCAGATTGTGTCGACAGGTAATGGTTGTCTGGTAAAAGGACAGGGCC    180
GB-17          ATCTTTCGAAAGGGCAGATTGTGTCGACAGGTAATGGTTGTCTGGTAAAAGGACAGGGCC    180
GB-18          ATCTTTCGAAAGGGCAGATTGTGTCGACAGGTAATGGTTGTCTGGTAAAAGGACAGGGCC    180
GB-19          ATCTTTCGAAAGGGCAGATTGTGTCGACAGGTAATGGTTGTCTGGTAAAAGGACAGGGCC    180
GB-20          ATCTTTCGAAAGGGCAGATTGTGTCGACAGGTAATGGTTGTCTGGTAAAAGGACAGGGCC    180
GB-21          ATCTTTCGAAAGGGCAGATTGTGTCGACAGGTAATGGTTGTCTGGTAAAAGGACAGGGCC    180
GB-22          ATCTTTCGAAAGGGCAGATTGTGTCGACAGGTAATGGTTGTCTGGTAAAAGGACAGGGCC    180
GB-23          ATCTTTCGAAAGGGCAGATTGTGTCGACAGGTAATGGTTGTCTGGTAAAAGGACAGGGCC    180
GB-24          ATCTTTCGAAAGGGCAGATTGTGTCGACAGGTAATGGTTGTCTGGTAAAAGGACAGGGCC    180
```

```
GB-25           ATCTTTCGAAAGGGCAGATTGTGTCGACAGGTAATGGTTGTCTGGTAAAAGGACAGGGCC    180
GB-27           ATCTTTCGAAAGGGCAGATTGTGTCGACAGGTAATGGTTGTCTGGTAAAAGGACAGGGCC    180
GB-29           ATCTTTCGAAAGGGCAGATTGTGTCGACAGGTAATGGTTGTCTGGTAAAAGGACAGGGCC    180
GB-30           ATCTTTCGAAAGGGCAGATTGTGTCGACAGGTAATGGTTGTCTGGTAAAAGGACAGGGCC    180
GB-31           ATCTTTCGAAAGGGCAGATTGTGTCGACAGGTAATGGTTGTCTGGTAAAAGGACAGGGCC    180
GB-33           ATCTTTCGAAAGGGCAGATTGTGTCGACAGGTAATGGTTGTCTGGTAAAAGGACAGGGCC    180
GB-34           ATCTTTCGAAAGGGCAGATTGTGTCGACAGGTAATGGTTGTCTGGTAAAAGGACAGGGCC    180
GB-36           ATCTTTCGAAAGGGCAGATTGTGTCGACAGGTAATGGTTGTCTGGTAAAAGGACAGGGCC    180
GB-38           ATCTTTCGAAAGGGCAGATTGTGTCGACAGGTAATGGTTGTCTGGTAAAAGGACAGGGCC    180
GB-40           ATCTTTCGAAAGGGCAGATTGTGTCGACAGGTAATGGTTGTCTGGTAAAAGGACAGGGCC    180
GB-41           ATCTTTCGAAAGGGCAGATTGTGTCGACAGGTAATGGTTGTCTGGTAAAAGGACAGGGCC    180
GB-42           ATCTTTCGAAAGGGCAGATTGTGTCGACAGGTAATGGTTGTCTGGTAAAAGGACAGGGCC    180
GB-43           ATCTTTCGAAAGGGCAGATTGTGTCGACAGGTAATGGTTGTCTGGTAAAAGGACAGGGCC    180
GB-44           ATCTTTCGAAAGGGCAGATTGTGTCGACAGGTAATGGTTGTCTGGTAAAAGGACAGGGCC    180
GB-45           ATCTTTCGAAAGGGCAGATTGTGTCGACAGGTAATGGTTGTCTGGTAAAAGGACAGGGCC    180
GB-46           ATCTTTCGAAAGGGCAGATTGTGTCGACAGGTAATGGTTGTCTGGTAAAAGGACAGGGCC    180
GB-47           ATCTTTCGAAAGGGCAGATTGTGTCGACAGGTAATGGTTGTCTGGTAAAAGGACAGGGCC    180
                ************************************************************

800mer_Ref      ATCGCCAATTGGAGTATTTTGTTGATAATGGTCTGCTAGTTGAACGGATCCATCTTCAAT    240
GB-1            ATCGCCAATTGGAGTATTTTGTTGATAATGGTCTGCTAGTTGAACGGATCCATCTTCAAT    240
GB-2            ATCGCCAATTGGAGTATTTTGTTGATAATGGTCTGCTAGTTGAACGGATCCATCTTCAAT    240
GB-3            ATCGCCAATTGGAGTATTTTGTTGATAATGGTCTGCTAGTTGAACGGATCCATCTTCAAT    240
GB-4            ATCGCCAATTGGAGTATTTTGTTGATAATGGTCTGCTAGTTGAACGGATCCATCTTCAAT    240
GB-5            ATCGCCAATTGGAGTATTTTGTTGATAATGGTCTGCTAGTTGAACGGATCCATCTTCAAT    240
GB-7            ATCGCCAATTGGAGTATTTTGTTGATAATGGTCTGCTAGTTGAACGGATCCATCTTCAAT    240
GB-10           ATCGCCAATTGGAGTATTTTGTTGATAATGGTCTGCTAGTTGAACGGATCCATCTTCAAT    240
GB-12           ATCGCCAATTGGAGTATTTTGTTGATAATGGTCTGCTAGTTGAACGGATCCATCTTCAAT    240
GB-15           ATCGCCAATTGGAGTATTTTGTTGATAATGGTCTGCTAGTTGAACGGATCCATCTTCAAT    240
GB-16           ATCGCCAATTGGAGTATTTTGTTGATAATGGTCTGCTAGTTGAACGGATCCATCTTCAAT    240
GB-17           ATCGCCAATTGGAGTATTTTGTTGATAATGGTCTGCTAGTTGAACGGATCCATCTTCAAT    240
GB-18           ATCGCCAATTGGAGTATTTTGTTGATAATGGTCTGCTAGTTGAACGGATCCATCTTCAAT    240
GB-19           ATCGCCAATTGGAGTATTTTGTTGATAATGGTCTGCTAGTTGAACGGATCCATCTTCAAT    240
GB-20           ATCGCCAATTGGAGTATTTTGTTGATAATGGTCTGCTAGTTGAACGGATCCATCTTCAAT    240
GB-21           ATCGCCAATTGGAGTATTTTGTTGATAATGGTCTGCTAGTTGAACGGATCCATCTTCAAT    240
GB-22           ATCGCCAATTGGAGTATTTTGTTGATAATGGTCTGCTAGTTGAACGGATCCATCTTCAAT    240
GB-23           ATCGCCAATTGGAGTATTTTGTTGATAATGGTCTGCTAGTTGAACGGATCCATCTTCAAT    240
GB-24           ATCGCCAATTGGAGTATTTTGTTGATAATGGTCTGCTAGTTGAACGGATCCATCTTCAAT    240
GB-25           ATCGCCAATTGGAGTATTTTGTTGATAATGGTCTGCTAGTTGAACGGATCCATCTTCAAT    240
GB-27           ATCGCCAATTGGAGTATTTTGTTGATAATGGTCTGCTAGTTGAACGGATCCATCTTCAAT    240
GB-29           ATCGCCAATTGGAGTATTTTGTTGATAATGGTCTGCTAGTTGAACGGATCCATCTTCAAT    240
GB-30           ATCGCCAATTGGAGTATTTTGTTGATAATGGTCTGCTAGTTGAACGGATCCATCTTCAAT    240
GB-31           ATCGCCAATTGGAGTATTTTGTTGATAATGGTCTGCTAGTTGAACGGATCCATCTTCAAT    240
GB-33           ATCGCCAATTGGAGTATTTTGTTGATAATGGTCTGCTAGTTGAACGGATCCATCTTCAAT    240
GB-34           ATCGCCAATTGGAGTATTTTGTTGATAATGGTCTGCTAGTTGAACGGATCCATCTTCAAT    240
GB-36           ATCGCCAATTGGAGTATTTTGTTGATAATGGTCTGCTAGTTGAACGGATCCATCTTCAAT    240
GB-38           ATCGCCAATTGGAGTATTTTGTTGATAATGGTCTGCTAGTTGAACGGATCCATCTTCAAT    240
GB-40           ATCGCCAATTGGAGTATTTTGTTGATAATGGTCTGCTAGTTGAACGGATCCATCTTCAAT    240
GB-41           ATCGCCAATTGGAGTATTTTGTTGATAATGGTCTGCTAGTTGAACGGATCCATCTTCAAT    240
GB-42           ATCGCCAATTGGAGTATTTTGTTGATAATGGTCTGCTAGTTGAACGGATCCATCTTCAAT    240
```

```
GB-43          ATCGCCAATTGGAGTATTTTGTTGATAATGGTCTGCTAGTTGAACGGATCCATCTTCAAT    240
GB-44          ATCGCCAATTGGAGTATTTTGTTGATAATGGTCTGCTAGTTGAACGGATCCATCTTCAAT    240
GB-45          ATCGCCAATTGGAGTATTTTGTTGATAATGGTCTGCTAGTTGAACGGATCCATCTTCAAT    240
GB-46          ATCGCCAATTGGAGTATTTTGTTGATAATGGTCTGCTAGTTGAACGGATCCATCTTCAAT    240
GB-47          ATCGCCAATTGGAGTATTTTGTTGATAATGGTCTGCTAGTTGAACGGATCCATCTTCAAT    240
               ************************************************************

800mer_Ref     GTTGTGGCGAATTTTGAAGTTAGCTTTGATTCCATTCTTTTGTTTGTCTGCCGTGATGTA    300
GB-1           GTTGTGGCGAATTTTGAAGTTAGCTTTGATTCCATTCTTTTGTTTGTCTGCCGTGATGTA    300
GB-2           GTTGTGGCGAATTTTGAAGTTAGCTTTGATTCCATTCTTTTGTTTGTCTGCCGTGATGTA    300
GB-3           GTTGTGGCGAATTTTGAAGTTAGCTTTGATTCCATTCTTTTGTTTGTCTGCCGTGATGTA    300
GB-4           GTTGTGGCGAATTTTGAAGTTAGCTTTGATTCCATTCTTTTGTTTGTCTGCCGTGATGTA    300
GB-5           GTTGTGGCGAATTTTGAAGTTAGCTTTGATTCCATTCTTTTGTTTGTCTGCCGTGATGTA    300
GB-7           GTTGTGGCGAATTTTGAAGTTAGCTTTGATTCCATTCTTTTGTTTGTCTGCCGTGATGTA    300
GB-10          GTTGTGGCGAATTTTGAAGTTAGCTTTGATTCCATTCTTTTGTTTGTCTGCCGTGATGTA    300
GB-12          GTTGTGGCGAATTTTGAAGTTAGCTTTGATTCCATTCTTTTGTTTGTCTGCCGTGATGTA    300
GB-15          GTTGTGGCGAATTTTGAAGTTAGCTTTGATTCCATTCTTTTGTTTGTCTGCCGTGATGTA    300
GB-16          GTTGTGGCGAATTTTGAAGTTAGCTTTGATTCCATTCTTTTGTTTGTCTGCCGTGATGTA    300
GB-17          GTTGTGGCGAATTTTGAAGTTAGCTTTGATTCCATTCTTTTGTTTGTCTGCCGTGATGTA    300
GB-18          GTTGTGGCGAATTTTGAAGTTAGCTTTGATTCCATTCTTTTGTTTGTCTGCCGTGATGTA    300
GB-19          GTTGTGGCGAATTTTGAAGTTAGCTTTGATTCCATTCTTTTGTTTGTCTGCCGTGATGTA    300
GB-20          GTTGTGGCGAATTTTGAAGTTAGCTTTGATTCCATTCTTTTGTTTGTCTGCCGTGATGTA    300
GB-21          GTTGTGGCGAATTTTGAAGTTAGCTTTGATTCCATTCTTTTGTTTGTCTGCCGTGATGTA    300
GB-22          GTTGTGGCGAATTTTGAAGTTAGCTTTGATTCCATTCTTTTGTTTGTCTGCCGTGATGTA    300
GB-23          GTTGTGGCGAATTTTGAAGTTAGCTTTGATTCCATTCTTTTGTTTGTCTGCCGTGATGTA    300
GB-24          GTTGTGGCGAATTTTGAAGTTAGCTTTGATTCCATTCTTTTGTTTGTCTGCCGTGATGTA    300
GB-25          GTTGTGGCGAATTTTGAAGTTAGCTTTGATTCCATTCTTTTGTTTGTCTGCCGTGATGTA    300
GB-27          GTTGTGGCGAATTTTGAAGTTAGCTTTGATTCCATTCTTTTGTTTGTCTGCCGTGATGTA    300
GB-29          GTTGTGGCGAATTTTGAAGTTAGCTTTGATTCCATTCTTTTGTTTGTCTGCCGTGATGTA    300
GB-30          GTTGTGGCGAATTTTGAAGTTAGCTTTGATTCCATTCTTTTGTTTGTCTGCCGTGATGTA    300
GB-31          GTTGTGGCGAATTTTGAAGTTAGCTTTGATTCCATTCTTTTGTTTGTCTGCCGTGATGTA    300
GB-33          GTTGTGGCGAATTTTGAAGTTAGCTTTGATTCCATTCTTTTGTTTGTCTGCCGTGATGTA    300
GB-34          GTTGTGGCGAATTTTGAAGTTAGCTTTGATTCCATTCTTTTGTTTGTCTGCCGTGATGTA    300
GB-36          GTTGTGGCGAATTTTGAAGTTAGCTTTGATTCCATTCTTTTGTTTGTCTGCCGTGATGTA    300
GB-38          GTTGTGGCGAATTTTGAAGTTAGCTTTGATTCCATTCTTTTGTTTGTCTGCCGTGATGTA    300
GB-40          GTTGTGGCGAATTTTGAAGTTAGCTTTGATTCCATTCTTTTGTTTGTCTGCCGTGATGTA    300
GB-41          GTTGTGGCGAATTTTGAAGTTAGCTTTGATTCCATTCTTTTGTTTGTCTGCCGTGATGTA    300
GB-42          GTTGTGGCGAATTTTGAAGTTAGCTTTGATTCCATTCTTTTGTTTGTCTGCCGTGATGTA    300
GB-43          GTTGTGGCGAATTTTGAAGTTAGCTTTGATTCCATTCTTTTGTTTGTCTGCCGTGATGTA    300
GB-44          GTTGTGGCGAATTTTGAAGTTAGCTTTGATTCCATTCTTTTGTTTGTCTGCCGTGATGTA    300
GB-45          GTTGTGGCGAATTTTGAAGTTAGCTTTGATTCCATTCTTTTGTTTGTCTGCCGTGATGTA    300
GB-46          GTTGTGGCGAATTTTGAAGTTAGCTTTGATTCCATTCTTTTGTTTGTCTGCCGTGATGTA    300
GB-47          GTTGTGGCGAATTTTGAAGTTAGCTTTGATTCCATTCTTTTGTTTGTCTGCCGTGATGTA    300
               ************************************************************

800mer_Ref     TACATTGTGTGAGTTATAGTTGTACTCGAGTTTGTGTCCGAGAATGTTTCCATCTTCTTT    360
GB-1           TACATTGTGTGAGTTATAGTTGTACTCGAGTTTGTGTCCGAGAATGTTTCCATCTTCTTT    360
GB-2           TACATTGTGTGAGTTATAGTTGTACTCGAGTTTGTGTCCGAGAATGTTTCCATCTTCTTT    360
GB-3           TACATTGTGTGAGTTATAGTTGTACTCGAGTTTGTGTCCGAGAATGTTTCCATCTTCTTT    360
GB-4           TACATTGTGTGAGTTATAGTTGTACTCGAGTTTGTGTCCGAGAATGTTTCCATCTTCTTT    360
```

```
GB-5            TACATTGTGTGAGTTATAGTTGTACTCGAGTTTGTGTCCGAGAATGTTTCCATCTTCTTT    360
GB-7            TACATTGTGTGAGTTATAGTTGTACTCGAGTTTGTGTCCGAGAATGTTTCCATCTTCTTT    360
GB-10           TACATTGTGTGAGTTATAGTTGTACTCGAGTTTGTGTCCGAGAATGTTTCCATCTTCTTT    360
GB-12           TACATTGTGTGAGTTATAGTTGTACTCGAGTTTGTGTCCGAGAATGTTTCCATCTTCTTT    360
GB-15           TACATTGTGTGAGTTATAGTTGTACTCGAGTTTGTGTCCGAGAATGTTTCCATCTTCTTT    360
GB-16           TACATTGTGTGAGTTATAGTTGTACTCGAGTTTGTGTCCGAGAATGTTTCCATCTTCTTT    360
GB-17           TACATTGTGTGAGTTATAGTTGTACTCGAGTTTGTGTCCGAGAATGTTTCCATCTTCTTT    360
GB-18           TACATTGTGTGAGTTATAGTTGTACTCGAGTTTGTGTCCGAGAATGTTTCCATCTTCTTT    360
GB-19           TACATTGTGTGAGTTATAGTTGTACTCGAGTTTGTGTCCGAGAATGTTTCCATCTTCTTT    360
GB-20           TACATTGTGTGAGTTATAGTTGTACTCGAGTTTGTGTCCGAGAATGTTTCCATCTTCTTT    360
GB-21           TACATTGTGTGAGTTATAGTTGTACTCGAGTTTGTGTCCGAGAATGTTTCCATCTTCTTT    360
GB-22           TACATTGTGTGAGTTATAGTTGTACTCGAGTTTGTGTCCGAGAATGTTTCCATCTTCTTT    360
GB-23           TACATTGTGTGAGTTATAGTTGTACTCGAGTTTGTGTCCGAGAATGTTTCCATCTTCTTT    360
GB-24           TACATTGTGTGAGTTATAGTTGTACTCGAGTTTGTGTCCGAGAATGTTTCCATCTTCTTT    360
GB-25           TACATTGTGTGAGTTATAGTTGTACTCGAGTTTGTGTCCGAGAATGTTTCCATCTTCTTT    360
GB-27           TACATTGTGTGAGTTATAGTTGTACTCGAGTTTGTGTCCGAGAATGTTTCCATCTTCTTT    360
GB-29           TACATTGTGTGAGTTATAGTTGTACTCGAGTTTGTGTCCGAGAATGTTTCCATCTTCTTT    360
GB-30           TACATTGTGTGAGTTATAGTTGTACTCGAGTTTGTGTCCGAGAATGTTTCCATCTTCTTT    360
GB-31           TACATTGTGTGAGTTATAGTTGTACTCGAGTTTGTGTCCGAGAATGTTTCCATCTTCTTT    360
GB-33           TACATTGTGTGAGTTATAGTTGTACTCGAGTTTGTGTCCGAGAATGTTTCCATCTTCTTT    360
GB-34           TACATTGTGTGAGTTATAGTTGTACTCGAGTTTGTGTCCGAGAATGTTTCCATCTTCTTT    360
GB-36           TACATTGTGTGAGTTATAGTTGTACTCGAGTTTGTGTCCGAGAATGTTTCCATCTTCTTT    360
GB-38           TACATTGTGTGAGTTATAGTTGTACTCGAGTTTGTGTCCGAGAATGTTTCCATCTTCTTT    360
GB-40           TACATTGTGTGAGTTATAGTTGTACTCGAGTTTGTGTCCGAGAATGTTTCCATCTTCTTT    360
GB-41           TACATTGTGTGAGTTATAGTTGTACTCGAGTTTGTGTCCGAGAATGTTTCCATCTTCTTT    360
GB-42           TACATTGTGTGAGTTATAGTTGTACTCGAGTTTGTGTCCGAGAATGTTTCCATCTTCTTT    360
GB-43           TACATTGTGTGAGTTATAGTTGTACTCGAGTTTGTGTCCGAGAATGTTTCCATCTTCTTT    360
GB-44           TACATTGTGTGAGTTATAGTTGTACTCGAGTTTGTGTCCGAGAATGTTTCCATCTTCTTT    360
GB-45           TACATTGTGTGAGTTATAGTTGTACTCGAGTTTGTGTCCGAGAATGTTTCCATCTTCTTT    360
GB-46           TACATTGTGTGAGTTATAGTTGTACTCGAGTTTGTGTCCGAGAATGTTTCCATCTTCTTT    360
GB-47           TACATTGTGTGAGTTATAGTTGTACTCGAGTTTGTGTCCGAGAATGTTTCCATCTTCTTT    360
                ************************************************************

800mer_Ref      AAAATCAATACCTTTTAACTCGATACGATTAACAAGGGTATCACCTTCAAACTTGACTTC    420
GB-1            AAAATCAATACCTTTTAACTCGATACGATTAACAAGGGTATCACCTTCAAACTTGACTTC    420
GB-2            AAAATCAATACCTTTTAACTCGATACGATTAACAAGGGTATCACCTTCAAACTTGACTTC    420
GB-3            AAAATCAATACCTTTTAACTCGATACGATTAACAAGGGTATCACCTTCAAACTTGACTTC    420
GB-4            AAAATCAATACCTTTTAACTCGATACGATTAACAAGGGTATCACCTTCAAACTTGACTTC    420
GB-5            AAAATCAATACCTTTTAACTCGATACGATTAACAAGGGTATCACCTTCAAACTTGACTTC    420
GB-7            AAAATCAATACCTTTTAACTCGATACGATTAACAAGGGTATCACCTTCAAACTTGACTTC    420
GB-10           AAAATCAATACCTTTTAACTCGATACGATTAACAAGGGTATCACCTTCAAACTTGACTTC    420
GB-12           AAAATCAATACCTTTTAACTCGATACGATTAACAAGGGTATCACCTTCAAACTTGACTTC    420
GB-15           AAAATCAATACCTTTTAACTCGATACGATTAACAAGGGTATCACCTTCAAACTTGACTTC    420
GB-16           AAAATCAATACCTTTTAACTCGATACGATTAACAAGGGTATCACCTTCAAACTTGACTTC    420
GB-17           AAAATCAATACCTTTTAACTCGATACGATTAACAAGGGTATCACCTTCAAACTTGACTTC    420
GB-18           AAAATCAATACCTTTTAACTCGATACGATTAACAAGGGTATCACCTTCAAACTTGACTTC    420
GB-19           AAAATCAATACCTTTTAACTCGATACGATTAACAAGGGTATCACCTTCAAACTTGACTTC    420
GB-20           AAAATCAATACCTTTTAACTCGATACGATTAACAAGGGTATCACCTTCAAACTTGACTTC    420
GB-21           AAAATCAATACCTTTTAACTCGATACGATTAACAAGGGTATCACCTTCAAACTTGACTTC    420
GB-22           AAAATCAATACCTTTTAACTCGATACGATTAACAAGGGTATCACCTTCAAACTTGACTTC    420
```

```
GB-23          AAAATCAATACCTTTTAACTCGATACGATTAACAAGGGTATCACCTTCAAACTTGACTTC    420
GB-24          AAAATCAATACCTTTTAACTCGATACGATTAACAAGGGTATCACCTTCAAACTTGACTTC    420
GB-25          AAAATCAATACCTTTTAACTCGATACGATTAACAAGGGTATCACCTTCAAACTTGACTTC    420
GB-27          AAAATCAATACCTTTTAACTCGATACGATTAACAAGGGTATCACCTTCAAACTTGACTTC    420
GB-29          AAAATCAATACCTTTTAACTCGATACGATTAACAAGGGTATCACCTTCAAACTTGACTTC    420
GB-30          AAAATCAATACCTTTTAACTCGATACGATTAACAAGGGTATCACCTTCAAACTTGACTTC    420
GB-31          AAAATCAATACCTTTTAACTCGATACGATTAACAAGGGTATCACCTTCAAACTTGACTTC    420
GB-33          AAAATCAATACCTTTTAACTCGATACGATTAACAAGGGTATCACCTTCAAACTTGACTTC    420
GB-34          AAAATCAATACCTTTTAACTCGATACGATTAACAAGGGTATCACCTTCAAACTTGACTTC    420
GB-36          AAAATCAATACCTTTTAACTCGATACGATTAACAAGGGTATCACCTTCAAACTTGACTTC    420
GB-38          AAAATCAATACCTTTTAACTCGATACGATTAACAAGGGTATCACCTTCAAACTTGACTTC    420
GB-40          AAAATCAATACCTTTTAACTCGATACGATTAACAAGGGTATCACCTTCAAACTTGACTTC    420
GB-41          AAAATCAATACCTTTTAACTCGATACGATTAACAAGGGTATCACCTTCAAACTTGACTTC    420
GB-42          AAAATCAATACCTTTTAACTCGATACGATTAACAAGGGTATCACCTTCAAACTTGACTTC    420
GB-43          AAAATCAATACCTTTTAACTCGATACGATTAACAAGGGTATCACCTTCAAACTTGACTTC    420
GB-44          AAAATCAATACCTTTTAACTCGATACGATTAACAAGGGTATCACCTTCAAACTTGACTTC    420
GB-45          AAAATCAATACCTTTTAACTCGATACGATTAACAAGGGTATCACCTTCAAACTTGACTTC    420
GB-46          AAAATCAATACCTTTTAACTCGATACGATTAACAAGGGTATCACCTTCAAACTTGACTTC    420
GB-47          AAAATCAATACCTTTTAACTCGATACGATTAACAAGGGTATCACCTTCAAACTTGACTTC    420
               ************************************************************

800mer_Ref     AGCACGCGTCTTGTAGTTCCCGTCATCTTTGAAAGATATAGTGCGTTCCTGTACATAACC    480
GB-1           AGCACGCGTCTTGTAGTTCCCGTCATCTTTGAAAGATATAGTGCGTTCCTGTACATAACC    480
GB-2           AGCACGCGTCTTGTAGTTCCCGTCATCTTTGAAAGATATAGTGCGTTCCTGTACATAACC    480
GB-3           AGCACGCGTCTTGTAGTTCCCGTCATCTTTGAAAGATATAGTGCGTTCCTGTACATAACC    480
GB-4           AGCACGCGTCTTGTAGTTCCCGTCATCTTTGAAAGATATAGTGCGTTCCTGTACATAACC    480
GB-5           AGCACGCGTCTTGTAGTTCCCGTCATCTTTGAAAGATATAGTGCGTTCCTGTACATAACC    480
GB-7           AGCACGCGTCTTGTAGTTCCCGTCATCTTTGAAAGATATAGTGCGTTCCTGTACATAACC    480
GB-10          AGCACGCGTCTTGTAGTTCCCGTCATCTTTGAAAGATATAGTGCGTTCCTGTACATAACC    480
GB-12          AGCACGCGTCTTGTAGTTCCCGTCATCTTTGAAAGATATAGTGCGTTCCTGTACATAACC    480
GB-15          AGCACGCGTCTTGTAGTTCCCGTCATCTTTGAAAGATATAGTGCGTTCCTGTACATAACC    480
GB-16          AGCACGCGTCTTGTAGTTCCCGTCATCTTTGAAAGATATAGTGCGTTCCTGTACATAACC    480
GB-17          AGCACGCGTCTTGTAGTTCCCGTCATCTTTGAAAGATATAGTGCGTTCCTGTACATAACC    480
GB-18          AGCACGCGTCTTGTAGTTCCCGTCATCTTTGAAAGATATAGTGCGTTCCTGTACATAACC    480
GB-19          AGCACGCGTCTTGTAGTTCCCGTCATCTTTGAAAGATATAGTGCGTTCCTGTACATAACC    480
GB-20          AGCACGCGTCTTGTAGTTCCCGTCATCTTTGAAAGATATAGTGCGTTCCTGTACATAACC    480
GB-21          AGCACGCGTCTTGTAGTTCCCGTCATCTTTGAAAGATATAGTGCGTTCCTGTACATAACC    480
GB-22          AGCACGCGTCTTGTAGTTCCCGTCATCTTTGAAAGATATAGTGCGTTCCTGTACATAACC    480
GB-23          AGCACGCGTCTTGTAGTTCCCGTCATCTTTGAAAGATATAGTGCGTTCCTGTACATAACC    480
GB-24          AGCACGCGTCTTGTAGTTCCCGTCATCTTTGAAAGATATAGTGCGTTCCTGTACATAACC    480
GB-25          AGCACGCGTCTTGTAGTTCCCGTCATCTTTGAAAGATATAGTGCGTTCCTGTACATAACC    480
GB-27          AGCACGCGTCTTGTAGTTCCCGTCATCTTTGAAAGATATAGTGCGTTCCTGTACATAACC    480
GB-29          AGCACGCGTCTTGTAGTTCCCGTCATCTTTGAAAGATATAGTGCGTTCCTGTACATAACC    480
GB-30          AGCACGCGTCTTGTAGTTCCCGTCATCTTTGAAAGATATAGTGCGTTCCTGTACATAACC    480
GB-31          AGCACGCGTCTTGTAGTTCCCGTCATCTTTGAAAGATATAGTGCGTTCCTGTACATAACC    480
GB-33          AGCACGCGTCTTGTAGTTCCCGTCATCTTTGAAAGATATAGTGCGTTCCTGTACATAACC    480
GB-34          AGCACGCGTCTTGTAGTTCCCGTCATCTTTGAAAGATATAGTGCGTTCCTGTACATAACC    480
GB-36          AGCACGCGTCTTGTAGTTCCCGTCATCTTTGAAAGATATAGTGCGTTCCTGTACATAACC    480
GB-38          AGCACGCGTCTTGTAGTTCCCGTCATCTTTGAAAGATATAGTGCGTTCCTGTACATAACC    480
GB-40          AGCACGCGTCTTGTAGTTCCCGTCATCTTTGAAAGATATAGTGCGTTCCTGTACATAACC    480
```

```
GB-41          AGCACGCGTCTTGTAGTTCCCGTCATCTTTGAAAGATATAGTGCGTTCCTGTACATAACC    480
GB-42          AGCACGCGTCTTGTAGTTCCCGTCATCTTTGAAAGATATAGTGCGTTCCTGTACATAACC    480
GB-43          AGCACGCGTCTTGTAGTTCCCGTCATCTTTGAAAGATATAGTGCGTTCCTGTACATAACC    480
GB-44          AGCACGCGTCTTGTAGTTCCCGTCATCTTTGAAAGATATAGTGCGTTCCTGTACATAACC    480
GB-45          AGCACGCGTCTTGTAGTTCCCGTCATCTTTGAAAGATATAGTGCGTTCCTGTACATAACC    480
GB-46          AGCACGCGTCTTGTAGTTCCCGTCATCTTTGAAAGATATAGTGCGTTCCTGTACATAACC    480
GB-47          AGCACGCGTCTTGTAGTTCCCGTCATCTTTGAAAGATATAGTGCGTTCCTGTACATAACC    480
               ************************************************************

800mer_Ref     TTCGGGCATGGCACTCTTGAAAAAGTCATGCCGTTTCATATGATCCGGATAACGGGAAAA    540
GB-1           TTCGGGCATGGCACTCTTGAAAAAGTCATGCCGTTTCATATGATCCGGATAACGGGAAAA    540
GB-2           TTCGGGCATGGCACTCTTGAAAAAGTCATGCCGTTTCATATGATCCGGATAACGGGAAAA    540
GB-3           TTCGGGCATGGCACTCTTGAAAAAGTCATGCCGTTTCATATGATCCGGATAACGGGAAAA    540
GB-4           TTCGGGCATGGCACTCTTGAAAAAGTCATGCCGTTTCATATGATCCGGATAACGGGAAAA    540
GB-5           TTCGGGCATGGCACTCTTGAAAAAGTCATGCCGTTTCATATGATCCGGATAACGGGAAAA    540
GB-7           TTCGGGCATGGCACTCTTGAAAAAGTCATGCCGTTTCATATGATCCGGATAACGGGAAAA    540
GB-10          TTCGGGCATGGCACTCTTGAAAAAGTCATGCCGTTTCATATGATCCGGATAACGGGAAAA    540
GB-12          TTCGGGCATGGCACTCTTGAAAAAGTCATGCCGTTTCATATGATCCGGATAACGGGAAAA    540
GB-15          TTCGGGCATGGCACTCTTGAAAAAGTCATGCCGTTTCATATGATCCGGATAACGGGAAAA    540
GB-16          TTCGGGCATGGCACTCTTGAAAAAGTCATGCCGTTTCATATGATCCGGATAACGGGAAAA    540
GB-17          TTCGGGCATGGCACTCTTGAAAAAGTCATGCCGTTTCATATGATCCGGATAACGGGAAAA    540
GB-18          TTCGGGCATGGCACTCTTGAAAAAGTCATGCCGTTTCATATGATCCGGATAACGGGAAAA    540
GB-19          TTCGGGCATGGCACTCTTGAAAAAGTCATGCCGTTTCATATGATCCGGATAACGGGAAAA    540
GB-20          TTCGGGCATGGCACTCTTGAAAAAGTCATGCCGTTTCATATGATCCGGATAACGGGAAAA    540
GB-21          TTCGGGCATGGCACTCTTGAAAAAGTCATGCCGTTTCATATGATCCGGATAACGGGAAAA    540
GB-22          TTCGGGCATGGCACTCTTGAAAAAGTCATGCCGTTTCATATGATCCGGATAACGGGAAAA    540
GB-23          TTCGGGCATGGCACTCTTGAAAAAGTCATGCCGTTTCATATGATCCGGATAACGGGAAAA    540
GB-24          TTCGGGCATGGCACTCTTGAAAAAGTCATGCCGTTTCATATGATCCGGATAACGGGAAAA    540
GB-25          TTCGGGCATGGCACTCTTGAAAAAGTCATGCCGTTTCATATGATCCGGATAACGGGAAAA    540
GB-27          TTCGGGCATGGCACTCTTGAAAAAGTCATGCCGTTTCATATGATCCGGATAACGGGAAAA    540
GB-29          TTCGGGCATGGCACTCTTGAAAAAGTCATGCCGTTTCATATGATCCGGATAACGGGAAAA    540
GB-30          TTCGGGCATGGCACTCTTGAAAAAGTCATGCCGTTTCATATGATCCGGATAACGGGAAAA    540
GB-31          TTCGGGCATGGCACTCTTGAAAAAGTCATGCCGTTTCATATGATCCGGATAACGGGAAAA    540
GB-33          TTCGGGCATGGCACTCTTGAAAAAGTCATGCCGTTTCATATGATCCGGATAACGGGAAAA    540
GB-34          TTCGGGCATGGCACTCTTGAAAAAGTCATGCCGTTTCATATGATCCGGATAACGGGAAAA    540
GB-36          TTCGGGCATGGCACTCTTGAAAAAGTCATGCCGTTTCATATGATCCGGATAACGGGAAAA    540
GB-38          TTCGGGCATGGCACTCTTGAAAAAGTCATGCCGTTTCATATGATCCGGATAACGGGAAAA    540
GB-40          TTCGGGCATGGCACTCTTGAAAAAGTCATGCCGTTTCATATGATCCGGATAACGGGAAAA    540
GB-41          TTCGGGCATGGCACTCTTGAAAAAGTCATGCCGTTTCATATGATCCGGATAACGGGAAAA    540
GB-42          TTCGGGCATGGCACTCTTGAAAAAGTCATGCCGTTTCATATGATCCGGATAACGGGAAAA    540
GB-43          TTCGGGCATGGCACTCTTGAAAAAGTCATGCCGTTTCATATGATCCGGATAACGGGAAAA    540
GB-44          TTCGGGCATGGCACTCTTGAAAAAGTCATGCCGTTTCATATGATCCGGATAACGGGAAAA    540
GB-45          TTCGGGCATGGCACTCTTGAAAAAGTCATGCCGTTTCATATGATCCGGATAACGGGAAAA    540
GB-46          TTCGGGCATGGCACTCTTGAAAAAGTCATGCCGTTTCATATGATCCGGATAACGGGAAAA    540
GB-47          TTCGGGCATGGCACTCTTGAAAAAGTCATGCCGTTTCATATGATCCGGATAACGGGAAAA    540
               ************************************************************

800mer_Ref     GCATTGAACACCATAAGAGAAAGTAGTGACAAGTGTTGGCCATGGAACAGGTAGTTTTCC    600
GB-1           GCATTGAACACCATAAGAGAAAGTAGTGACAAGTGTTGGCCATGGAACAGGTAGTTTTCC    600
GB-2           GCATTGAACACCATAAGAGAAAGTAGTGACAAGTGTTGGCCATGGAACAGGTAGTTTTCC    600
```

```
GB-3            GCATTGAACACCATAAGAGAAAGTAGTGACAAGTGTTGGCCATGGAACAGGTAGTTTTCC    600
GB-4            GCATTGAACACCATAAGAGAAAGTAGTGACAAGTGTTGGCCATGGAACAGGTAGTTTTCC    600
GB-5            GCATTGAACACCATAAGAGAAAGTAGTGACAAGTGTTGGCCATGGAACAGGTAGTTTTCC    600
GB-7            GCATTGAACACCATAAGAGAAAGTAGTGACAAGTGTTGGCCATGGAACAGGTAGTTTTCC    600
GB-10           GCATTGAACACCATAAGAGAAAGTAGTGACAAGTGTTGGCCATGGAACAGGTAGTTTTCC    600
GB-12           GCATTGAACACCATAAGAGAAAGTAGTGACAAGTGTTGGCCATGGAACAGGTAGTTTTCC    600
GB-15           GCATTGAACACCATAAGAGAAAGTAGTGACAAGTGTTGGCCATGGAACAGGTAGTTTTCC    600
GB-16           GCATTGAACACCATAAGAGAAAGTAGTGACAAGTGTTGGCCATGGAACAGGTAGTTTTCC    600
GB-17           GCATTGAACACCATAAGAGAAAGTAGTGACAAGTGTTGGCCATGGAACAGGTAGTTTTCC    600
GB-18           GCATTGAACACCATAAGAGAAAGTAGTGACAAGTGTTGGCCATGGAACAGGTAGTTTTCC    600
GB-19           GCATTGAACACCATAAGAGAAAGTAGTGACAAGTGTTGGCCATGGAACAGGTAGTTTTCC    600
GB-20           GCATTGAACACCATAAGAGAAAGTAGTGACAAGTGTTGGCCATGGAACAGGTAGTTTTCC    600
GB-21           GCATTGAACACCATAAGAGAAAGTAGTGACAAGTGTTGGCCATGGAACAGGTAGTTTTCC    600
GB-22           GCATTGAACACCATAAGAGAAAGTAGTGACAAGTGTTGGCCATGGAACAGGTAGTTTTCC    600
GB-23           GCATTGAACACCATAAGAGAAAGTAGTGACAAGTGTTGGCCATGGAACAGGTAGTTTTCC    600
GB-24           GCATTGAACACCATAAGAGAAAGTAGTGACAAGTGTTGGCCATGGAACAGGTAGTTTTCC    600
GB-25           GCATTGAACACCATAAGAGAAAGTAGTGACAAGTGTTGGCCATGGAACAGGTAGTTTTCC    600
GB-27           GCATTGAACACCATAAGAGAAAGTAGTGACAAGTGTTGGCCATGGAACAGGTAGTTTTCC    600
GB-29           GCATTGAACACCATAAGAGAAAGTAGTGACAAGTGTTGGCCATGGAACAGGTAGTTTTCC    600
GB-30           GCATTGAACACCATAAGAGAAAGTAGTGACAAGTGTTGGCCATGGAACAGGTAGTTTTCC    600
GB-31           GCATTGAACACCATAAGAGAAAGTAGTGACAAGTGTTGGCCATGGAACAGGTAGTTTTCC    600
GB-33           GCATTGAACACCATAAGAGAAAGTAGTGACAAGTGTTGGCCATGGAACAGGTAGTTTTCC    600
GB-34           GCATTGAACACCATAAGAGAAAGTAGTGACAAGTGTTGGCCATGGAACAGGTAGTTTTCC    600
GB-36           GCATTGAACACCATAAGAGAAAGTAGTGACAAGTGTTGGCCATGGAACAGGTAGTTTTCC    600
GB-38           GCATTGAACACCATAAGAGAAAGTAGTGACAAGTGTTGGCCATGGAACAGGTAGTTTTCC    600
GB-40           GCATTGAACACCATAAGAGAAAGTAGTGACAAGTGTTGGCCATGGAACAGGTAGTTTTCC    600
GB-41           GCATTGAACACCATAAGAGAAAGTAGTGACAAGTGTTGGCCATGGAACAGGTAGTTTTCC    600
GB-42           GCATTGAACACCATAAGAGAAAGTAGTGACAAGTGTTGGCCATGGAACAGGTAGTTTTCC    600
GB-43           GCATTGAACACCATAAGAGAAAGTAGTGACAAGTGTTGGCCATGGAACAGGTAGTTTTCC    600
GB-44           GCATTGAACACCATAAGAGAAAGTAGTGACAAGTGTTGGCCATGGAACAGGTAGTTTTCC    600
GB-45           GCATTGAACACCATAAGAGAAAGTAGTGACAAGTGTTGGCCATGGAACAGGTAGTTTTCC    600
GB-46           GCATTGAACACCATAAGAGAAAGTAGTGACAAGTGTTGGCCATGGAACAGGTAGTTTTCC    600
GB-47           GCATTGAACACCATAAGAGAAAGTAGTGACAAGTGTTGGCCATGGAACAGGTAGTTTTCC    600
                ************************************************************

800mer_Ref      AGTAGTGCAAATAAATTTAAGGGTAAGTTTTCCGTATGTTGCATCACCTTCACCCTCTCC    660
GB-1            AGTAGTGCAAATAAATTTAAGGGTAAGTTTTCCGTATGTTGCATCACCTTCACCCTCTCC    660
GB-2            AGTAGTGCAAATAAATTTAAGGGTAAGTTTTCCGTATGTTGCATCACCTTCACCCTCTCC    660
GB-3            AGTAGTGCAAATAAATTTAAGGGTAAGTTTTCCGTATGTTGCATCACCTTCACCCTCTCC    660
GB-4            AGTAGTGCAAATAAATTTAAGGGTAAGTTTTCCGTATGTTGCATCACCTTCACCCTCTCC    660
GB-5            AGTAGTGCAAATAAATTTAAGGGTAAGTTTTCCGTATGTTGCATCACCTTCACCCTCTCC    660
GB-7            AGTAGTGCAAATAAATTTAAGGGTAAGTTTTCCGTATGTTGCATCACCTTCACCCTCTCC    660
GB-10           AGTAGTGCAAATAAATTTAAGGGTAAGTTTTCCGTATGTTGCATCACCTTCACCCTCTCC    660
GB-12           AGTAGTGCAAATAAATTTAAGGGTAAGTTTTCCGTATGTTGCATCACCTTCACCCTCTCC    660
GB-15           AGTAGTGCAAATAAATTTAAGGGTAAGTTTTCCGTATGTTGCATCACCTTCACCCTCTCC    660
GB-16           AGTAGTGCAAATAAATTTAAGGGTAAGTTTTCCGTATGTTGCATCACCTTCACCCTCTCC    660
GB-17           AGTAGTGCAAATAAATTTAAGGGTAAGTTTTCCGTATGTTGCATCACCTTCACCCTCTCC    660
GB-18           AGTAGTGCAAATAAATTTAAGGGTAAGTTTTCCGTATGTTGCATCACCTTCACCCTCTCC    660
GB-19           AGTAGTGCAAATAAATTTAAGGGTAAGTTTTCCGTATGTTGCATCACCTTCACCCTCTCC    660
GB-20           AGTAGTGCAAATAAATTTAAGGGTAAGTTTTCCGTATGTTGCATCACCTTCACCCTCTCC    660
```

```
GB-21           AGTAGTGCAAATAAATTTAAGGGTAAGTTTTCCGTATGTTGCATCACCTTCACCCTCTCC    660
GB-22           AGTAGTGCAAATAAATTTAAGGGTAAGTTTTCCGTATGTTGCATCACCTTCACCCTCTCC    660
GB-23           AGTAGTGCAAATAAATTTAAGGGTAAGTTTTCCGTATGTTGCATCACCTTCACCCTCTCC    660
GB-24           AGTAGTGCAAATAAATTTAAGGGTAAGTTTTCCGTATGTTGCATCACCTTCACCCTCTCC    660
GB-25           AGTAGTGCAAATAAATTTAAGGGTAAGTTTTCCGTATGTTGCATCACCTTCACCCTCTCC    660
GB-27           AGTAGTGCAAATAAATTTAAGGGTAAGTTTTCCGTATGTTGCATCACCTTCACCCTCTCC    660
GB-29           AGTAGTGCAAATAAATTTAAGGGTAAGTTTTCCGTATGTTGCATCACCTTCACCCTCTCC    660
GB-30           AGTAGTGCAAATAAATTTAAGGGTAAGTTTTCCGTATGTTGCATCACCTTCACCCTCTCC    660
GB-31           AGTAGTGCAAATAAATTTAAGGGTAAGTTTTCCGTATGTTGCATCACCTTCACCCTCTCC    660
GB-33           AGTAGTGCAAATAAATTTAAGGGTAAGTTTTCCGTATGTTGCATCACCTTCACCCTCTCC    660
GB-34           AGTAGTGCAAATAAATTTAAGGGTAAGTTTTCCGTATGTTGCATCACCTTCACCCTCTCC    660
GB-36           AGTAGTGCAAATAAATTTAAGGGTAAGTTTTCCGTATGTTGCATCACCTTCACCCTCTCC    660
GB-38           AGTAGTGCAAATAAATTTAAGGGTAAGTTTTCCGTATGTTGCATCACCTTCACCCTCTCC    660
GB-40           AGTAGTGCAAATAAATTTAAGGGTAAGTTTTCCGTATGTTGCATCACCTTCACCCTCTCC    660
GB-41           AGTAGTGCAAATAAATTTAAGGGTAAGTTTTCCGTATGTTGCATCACCTTCACCCTCTCC    660
GB-42           AGTAGTGCAAATAAATTTAAGGGTAAGTTTTCCGTATGTTGCATCACCTTCACCCTCTCC    660
GB-43           AGTAGTGCAAATAAATTTAAGGGTAAGTTTTCCGTATGTTGCATCACCTTCACCCTCTCC    660
GB-44           AGTAGTGCAAATAAATTTAAGGGTAAGTTTTCCGTATGTTGCATCACCTTCACCCTCTCC    660
GB-45           AGTAGTGCAAATAAATTTAAGGGTAAGTTTTCCGTATGTTGCATCACCTTCACCCTCTCC    660
GB-46           AGTAGTGCAAATAAATTTAAGGGTAAGTTTTCCGTATGTTGCATCACCTTCACCCTCTCC    660
GB-47           AGTAGTGCAAATAAATTTAAGGGTAAGTTTTCCGTATGTTGCATCACCTTCACCCTCTCC    660
                ************************************************************

800mer_Ref      ACTGACAGAAAATTTGTGCCCATTAACATCACCATCTAATTCAACAAGAATTGGGACAAC    720
GB-1            ACTGACAGAAAATTTGTGCCCATTAACATCACCATCTAATTCAACAAGAATTGGGAC-AC    719
GB-2            ACTGACAGAAAATTTGTGCCCATTAACATCACCATCTAATTCAACAAGAATTGGGACAAC    720
GB-3            ACTGACAGAAAATTTGTGCCCATTAACATCACCATCTAATTCAACAAGAATTGGGACAAC    720
GB-4            ACTGACAGAAAATTTGTGCCCATTAACATCACCATCTAATTCAACAAGAATTGGGACAAC    720
GB-5            ACTGACAGAAAATTTGTGCCCATTAACATCACCATCTAATTCAACAAGAATTGGGACAAC    720
GB-7            ACTGACAGAAAATTTGTGCCCATTAACATCACCATCTAATTCAACAAGAATTGGGACAAC    720
GB-10           ACTGACAGAAAATTTGTGCCCATTAACATCACCATCTAATTCAACAAGAATTGGGACAAC    720
GB-12           ACTGACAGAAAATTTGTGCCCATTAACATCACCATCTAATTCAACAAGAATTGGGACAAC    720
GB-15           ACTGACAGAAAATTTGTGCCCATTAACATCACCATCTAATTCAACAAGAATTGGGACAAC    720
GB-16           ACTGACAGAAAATTTGTGCCCATTAACATCACCATCTAATTCAACAAGAATTGGGACAAC    720
GB-17           ACTGACAGAAAATTTGTGCCCATTAACATCACCATCTAATTCAACAAGAATTGGGACAAC    720
GB-18           ACTGACAGAAAATTTGTGCCCATTAACATCACCATCTAATTCAACAAGAATTGGGACAAC    720
GB-19           ACTGACAGAAAATTTGTGCCCATTAACATCACCATCTAATTCAACAAGAATTGGGACAAC    720
GB-20           ACTGACAGAAAATTTGTGCCCATTAACATCACCATCTAATTCAACAAGAATTGGGACAAC    720
GB-21           ACTGACAGAAAATTTGTGCCCATTAACATCACCATCTAATTCAACAAGAATTGGGACAAC    720
GB-22           ACTGACAGAAAATTTGTGCCCATTAACATCACCATCTAATTCAACAAGAATTGGGACAAC    720
GB-23           ACTGACAGAAAATTTGTGCCCATTAACATCACCATCTAATTCAACAAGAATTGGGACAAC    720
GB-24           ACTGACAGAAAATTTGTGCCCATTAACATCACCATCTAATTCAACAAGAATTGGGACAAC    720
GB-25           ACTGACAGAAAATTTGTGCCCATTAACATCACCATCTAATTCAACAAGAATTGGGACAAC    720
GB-27           ACTGACAGAAAATTTGTGCCCATTAACATCACCATCTAATTCAACAAGAATTGGGACAAC    720
GB-29           ACTGACAGAAAATTTGTGCCCATTAACATCACCATCTAATTCAACAAGAATTGGGACAAC    720
GB-30           ACTGACAGAAAATTTGTGCCCATTAACATCACCATCTAATTCAACAAGAATTGGGACAAC    720
GB-31           ACTGACAGAAAATTTGTGCCCATTAACATCACCATCTAATTCAACAAGAATTGGGACAAC    720
GB-33           ACTGACAGAAAATTTGTGCCCATTAACATCACCATCTAATTCAACAAGAATTGGGACAAC    720
GB-34           ACTGACAGAAAATTTGTGCCCATTAACATCACCATCTAATTCAACAAGAATTGGGACAAC    720
GB-36           ACTGACAGAAAATTTGTGCCCATTAACATCACCATCTAATTCAACAAGAATTGGGACAAC    720
```

```
GB-38         ACTGACAGAAAATTTGTGCCCATTAACATCACCATCTAATTCAACAAGAATTGGGACAAC    720
GB-40         ACTGACAGAAAATTTGTGCCCATTAACATCACCATCTAATTCAACAAGAATTGGGACAAC    720
GB-41         ACTGACAGAAAATTTGTGCCCATTAACATCACCATCTAATTCAACAAGAATTGGGACAAC    720
GB-42         ACTGACAGAAAATTTGTGCCCATTAACATCACCATCTAATTCAACAAGAATTGGGACAAC    720
GB-43         ACTGACAGAAAATTTGTGCCCATTAACATCACCATCTAATTCAACAAGAATTGGGACAAC    720
GB-44         ACTGACAGAAAATTTGTGCCCATTAACATCACCATCTAATTCAACAAGAATTGGGACAAC    720
GB-45         ACTGACAGAAAATTTGTGCCCATTAACATCACCATCTAATTCAACAAGAATTGGGACAAC    720
GB-46         ACTGACAGAAAATTTGTGCCCATTAACATCACCATCTAATTCAACAAGAATTGGGACAAC    720
GB-47         ACTGACAGAAAATTTGTGCCCATTAACATCACCATCTAATTCAACAAGAATTGGGACAAC    720
              ********************************************************* **

800mer_Ref    TCCAGTGAAAAGTTCTTCTCCTTTACTCATATTTTTTCCTCCTTATACTTAAGCCCTATA    780
GB-1          TCCAGTGAAAAGTTCTTCTCCTTTACTCATATTTTTTCCTCCTTATACTTAAGCCCTATA    779
GB-2          TCCAGTGAAAAGTTCTTCTCCTTTACTCATATTTTTTCCTCCTTATACTTAAGCCCTATA    780
GB-3          TCCAGTGAAAAGTTCTTCTCCTTTACTCATATTTTTTCCTCCTTATACTTAAGCCCTATA    780
GB-4          TCCAGTGAAAAGTTCTTCTCCTTTACTCATATTTTTTCCTCCTTATACTTAAGCCCTATA    780
GB-5          TCCAGTGAAAAGTTCTTCTCCTTTACTCATATTTTTTCCTCCTTATACTTAAGCCCTATA    780
GB-7          TCCAGTGAAAAGTTCTTCTCCTTTACTCATATTTTTTCCTCCTTATACTTAAGCCCTATA    780
GB-10         TCCAGTGAAAAGTTCTTCTCCTTTACTCATATTTTTTCCTCCTTATACTTAAGCCCTATA    780
GB-12         TCCAGTGAAAAGTTCTTCTCCTTTACTCATATTTTTTCCTCCTTATACTTAAGCCCTATA    780
GB-15         TCCAGTGAAAAGTTCTTCTCCTTTACTCATATTTTTTCCTCCTTATACTTAAGCCCTATA    780
GB-16         TCCAGTGAAAAGTTCTTCTCCTTTACTCATATTTTTTCCTCCTTATACTTAAGCCCTATA    780
GB-17         TCCAGTGAAAAGTTCTTCTCCTTTACTCATATTTTTTCCTCCTTATACTTAAGCCCTATA    780
GB-18         TCCAGTGAAAAGTTCTTCTCCTTTACTCATATTTTTTCCTCCTTATACTTAAGCCCTATA    780
GB-19         TCCAGTGAAAAGTTCTTCTCCTTTACTCATATTTTTTCCTCCTTATACTTAAGCCCTATA    780
GB-20         TCCAGTGAAAAGTTCTTCTCCTTTACTCATATTTTTTCCTCCTTATACTTAAGCCCTATA    780
GB-21         TCCAGTGAAAAGTTCTTCTCCTTTACTCATATTTTTTCCTCCTTATACTTAAGCCCTATA    780
GB-22         TCCAGTGAAAAGTTCTTCTCCTTTACTCATATTTTTTCCTCCTTATACTTAAGCCCTATA    780
GB-23         TCCAGTGAAAAGTTCTTCTCCTTTACTCATATTTTTTCCTCCTTATACTTAAGCCCTATA    780
GB-24         TCCAGTGAAAAGTTCTTCTCCTTTACTCATATTTTTTCCTCCTTATACTTAAGCCCTATA    780
GB-25         TCCAGTGAAAAGTTCTTCTCCTTTACTCATATTTTTTCCTCCTTATACTTAAGCCCTATA    780
GB-27         TCCAGTGAAAAGTTCTTCTCCTTTACTCATATTTTTTCCTCCTTATACTTAAGCCCTATA    780
GB-29         TCCAGTGAAAAGTTCTTCTCCTTTACTCATATTTTTTCCTCCTTATACTTAAGCCCTATA    780
GB-30         TCCAGTGAAAAGTTCTTCTCCTTTACTCATATTTTTTCCTCCTTATACTTAAGCCCTATA    780
GB-31         TCCAGTGAAAAGTTCTTCTCCTTTACTCATATTTTTTCCTCCTTATACTTAAGCCCTATA    780
GB-33         TCCAGTGAAAAGTTCTTCTCCTTTACTCATATTTTTTCCTCCTTATACTTAAGCCCTATA    780
GB-34         TCCAGTGAAAAGTTCTTCTCCTTTACTCATATTTTTTCCTCCTTATACTTAAGCCCTATA    780
GB-36         TCCAGTGAAAAGTTCTTCTCCTTTACTCATATTTTTTCCTCCTTATACTTAAGCCCTATA    780
GB-38         TCCAGTGAAAAGTTCTTCTCCTTTACTCATATTTTTTCCTCCTTATACTTAAGCCCTATA    780
GB-40         TCCAGTGAAAAGTTCTTCTCCTTTACTCATATTTTTTCCTCCTTATACTTAAGCCCTATA    780
GB-41         TCCAGTGAAAAGTTCTTCTCCTTTACTCATATTTTTTCCTCCTTATACTTAAGCCCTATA    780
GB-42         TCCAGTGAAAAGTTCTTCTCCTTTACTCATATTTTTTCCTCCTTATACTTAAGCCCTATA    780
GB-43         TCCAGTGAAAAGTTCTTCTCCTTTACTCATATTTTTTCCTCCTTATACTTAAGCCCTATA    780
GB-44         TCCAGTGAAAAGTTCTTCTCCTTTACTCATATTTTTTCCTCCTTATACTTAAGCCCTATA    780
GB-45         TCCAGTGAAAAGTTCTTCTCCTTTACTCATATTTTTTCCTCCTTATACTTAAGCCCTATA    780
GB-46         TCCAGTGAAAAGTTCTTCTCCTTTACTCATATTTTTTCCTCCTTATACTTAAGCCCTATA    780
GB-47         TCCAGTGAAAAGTTCTTCTCCTTTACTCATATTTTTTCCTCCTTATACTTAAGCCCTATA    780
              ************************************************************

800mer_Ref    GTGAGTCGTATTAATTCGC     799
```

GB-1	GTGAGTCGTATTAATTCGC	798
GB-2	GTGAGTCGTATTAATTCGC	799
GB-3	GTGAGTCGTATTAATTCGC	799
GB-4	GTGAGTCGTATTAATTCGC	799
GB-5	GTGAGTCGTATTAATTCGC	799
GB-7	GTGAGTCGTATTAATTCGC	799
GB-10	GTGAGTCGTATTAATTCGC	799
GB-12	GTGAGTCGTATTAATTCGC	799
GB-15	GTGAGTCGTATTAATTCGC	799
GB-16	GTGAGTCGTATTAATTCGC	799
GB-17	GTGAGTCGTATTAATTCGC	799
GB-18	GTGAGTCGTATTAATTCGC	799
GB-19	GTGAGTCGTATTAATTCGC	799
GB-20	GTGAGTCGTATTAATTCGC	799
GB-21	GTGAGTCGTATTAATTCGC	799
GB-22	GTGAGTCGTATTAATTCGC	799
GB-23	GTGAGTCGTATTAATTCGC	799
GB-24	GTGAGTCGTATTAATTCGC	799
GB-25	GTGAGTCGTATTAATTCGC	799
GB-27	GTGAGTCGTATTAATTCGC	799
GB-29	GTGAGTCGTATTAATTCGC	799
GB-30	GTGAGTCGTATTAATTCGC	799
GB-31	GTGAGTCGTATTAATTCGC	799
GB-33	GTGAGTCGTATTAATTCGC	799
GB-34	GTGAGTCGTATTAATTCGC	799
GB-36	GTGAGTCGTATTAATTCGC	799
GB-38	GTGAGTCGTATTAATTC--	797
GB-40	GTGAGTCGTATTAATTCGC	799
GB-41	GTGAGTCGTATTAATTCGC	799
GB-42	GTGAGTCGTATTAATTCGC	799
GB-43	GTGAGTCGTATTAATTCGC	799
GB-44	GTGAGTCGTATTAATTCGC	799
GB-45	GTGAGTCGTATTAATTCGC	799
GB-46	GTGAGTCGTATTAATTCGC	799
GB-47	GTGAGTCGTATTAATTCGC	799
	*****************	

Sequencing results for the 1,728-mer Phi29 DNA polymerase gene synthesized on glass beads (GB). "1728mer_Reference" is the reference sequence. The numbers in the names of the sequences correspond to the lane numbers in Figure 3G.

```
1728mer_Reference atgaagcatatgccgagaaagatgtatagttgtgactttgagacaactactaaagtggaa  60
1728mer_1         ATGAAGCATATGCCGAGAAAGATGTATAGTTGTGACTTTGAGACAACTACTAAAGTGGAA  60
1728mer_2         ATGAAGCATATGCCGAGAAAGATGTATAGTTGTGACTTTGAGACAACTACTAAAGTGGAA  60
1728mer_3         ATGAAGCATATGCCGAGAAAGATGTATAGTTGTGACTTTGAGACAACTACTAAAGTGGAA  60
1728mer_4         ATGAAGCATATGCCGAGAAAGATGTATAGTTGTGACTTTGAGACAACTACTAAAGTGGAA  60
1728mer_5         ATGAAGCATATGCCGAGAAAGATGTATAGTTGTGACTTTGAGACAACTACTAAAGTGGAA  60
1728mer_6         ATGAAGCATATGCCGAGAAAGATGTATAGTTGTGACTTTGAGACAACTACTAAAGTGGAA  60
1728mer_7         ATGAAGCATATGCCGAGAAAGATGTATAGTTGTGACTTTGAGACAACTACTAAAGCGGAA  60
1728mer_9         ATGAAGCATATGCCGAGAAAGATGTATAGTTGTGACTTTGAGACAACTACTAAAGTGGAA  60
1728mer_8         ATGAAGCATATGCCGAGAAAGATGTATAGTTGTGACTTTGAGACAACTACTAAAGTGGAA  60
1728mer_10        ATGAAGCATATGCCGAGAAAGATGTATAGTTGTGACTTTGAGACAACTACTAAAGTGGAA  60
1728mer_11        ATGAAGCATATGCCGAGAAAGATGTATAGTTGTGACTTTGAGACAACTACTAAAGTGGAA  60
1728mer_12        ATGAAGCATATGCCGAGAAAGATGTATAGTTGTGACTTTGAGACAACTACTAAAGTGGAA  60
1728mer_13        ATGAAGCATATGCCGAGAAAGATGTATAGTTGTGACTTTGAGACAACTACTAAAGTGGAA  60
1728mer_14        ATGAAGCATATGCCGAGAAAGATGTATAGTTGTGACTTTGAGACAACTACTAAAGTGGAA  60
1728mer_15        ATGAAGCATATGCCGAGAAAGATGTATAGTTGTGACTTTGAGACAACTACTAAAGTGGAA  60
1728mer_16        ATGAAGCATATGCCGAGAAAGATGTATAGTTGTGACTTTGAGACAACTACTAAAGTGGAA  60
                  ******************************************************* ****

1728mer_Reference gactgtagggtatgggcgtatggttatatgaatatagaagatcacagtgagtacaaaata  120
1728mer_1         GACTGTAGGGTATGGGCGTATGGTTATATGAATATAGAAGATCACAGTGAGTACAAAATA  120
1728mer_2         GACTGTAGGGTATGGGCGTATGGTTAT--GAATATAGAAGATCACAGTGAGTACAAAATA  118
1728mer_3         GACTGTAGGGTATGGGCGTATGGTTATATGAATATAGAAGATCACAGTGAGTACAAAATA  120
1728mer_4         GACTGTAGGGTATGGGCGTATGGTTATATGAATATAGAAGATCACAGTGAGTACAAAATA  120
1728mer_5         GACTGTAGGGTATGGGCGTATGGTTATATGAATATAGAAGATCACAGTGAGTACAAAATA  120
1728mer_6         GACTGTAGGGTATGGGCGTATGGTTATATGAATATAGAAGATCACAGTGAGTACAAAATA  120
1728mer_7         GACTGTAGGGTATGGGCGTATGGTTATATGAATATAGAAGATCACAGTGAGTACAAAATA  120
1728mer_9         GACTGTAGGGTATGGGCGTATGGTTATATGAATATAGAAGATCACAGTGAGTACAAAATA  120
1728mer_8         GACTGTAGGGTATGGGCGTATGGTTATATGAATATAGAAGATCACAGTGAGTACAAAATA  120
1728mer_10        GACTGTAGGGTATGGGCGTATGGTTATATGAATATAGAAGATCACAGTGAGTACAAAATA  120
1728mer_11        GACTGTAGGGTATGGGCGTATGGTTATATGAATATAGAAGATCACAGTGAGTACAAAATA  120
1728mer_12        GACTGTAGGGTATGGGCGTATGGTTATATGAATATAGAAGATCACAGTGAGTACAAAATA  120
1728mer_13        GACTGTAGGGTATGGGCGTATGGTTATATGAATATAGAAGATCACAGTGAGTACAAAATA  120
1728mer_14        GACTGTAGGGTATGGGCGTATGGTTATATGAATATAGAAGATCACAGTGAGTACAAAATA  120
1728mer_15        GACTGTAGGGTATGGGCGTATGGTTATATGAATATAGAAGATCACAGTGAGTACAAAATA  120
1728mer_16        GACTGTAGGGTATGGGCGTATGGTTATATGAATATAGAAGATCACAGTGAGTACAAAATA  120
                  ***************************  *******************************

1728mer_Reference ggtaatagcctggatgagtttatggcgtgggtgttgaaggtacaagctgatctatatttc  180
1728mer_1         GGTAATAGCCTGGATGAGTTTATGGCGTGGGTGTTGAAGGTACAAGCTGATCTATATTTC  180
1728mer_2         GGTAATAGCCTGGATGAGTTTATGGCGTGGGTGTTGAAGGTACAAGCTGATCTATATTTC  178
1728mer_3         GGTAATAGCCTGGATGAGTTTATGGCGTGGGTGTTGAAGGTACAAGCTGATCTATATTTC  180
1728mer_4         GGTAATAGCCTGGATGAGTTTATGGCGTGGGTGTTGAAGGTACAAGCTGATCTATATTTC  180
1728mer_5         GGTAATAGCCTGGATGAGTTTATGGCGTGGGTGTTGAAGGTACAAGCTGATCTATATTTC  180
1728mer_6         GGTAATAGCCTGGATGAGTTTATGGCGTGGGTGTTGAAGGTACAAGCTGATCTATATTTC  180
```

```
1728mer_7         GGTAATAGCCTGGATGAGTTTATGGCGTGGGTGTTGAAGGTACAAGCTGATCTATATTTC  180
1728mer_9         GGTAATAGCCTGGATGAGTTTATGGCGTGGGTGTTGAAGGTACAAGCTGATCTATATTTC  180
1728mer_8         GGTAATAGCCTGGATGAGTTTATGGCGTGGGTGTTGAAGGTACAAGCTGATCTATATTTC  180
1728mer_10        GGTAATAGCCTGGATGAGTTTATGGCGTGGGTGTTGAAGGTACAAGCTGATCTATATTTC  180
1728mer_11        GGTAATAGCCTGGATGAGTTTATGGCGTGGGTGTTGAAGGTACAAGCTGATCTATATTTC  180
1728mer_12        GGTAATAGCCTGGATGAGTTTATGGCGTGGGTGTTGAAGGTACAAGCTTATCTATATTTC  180
1728mer_13        GGTAATAGCCTGGATGAGTTTATGGCGTGGGTGTTGAAGGTACAAGCTGATCTATATTTC  180
1728mer_14        GGTAATAGCCTGGATGAGTTTATGGCGTGGGTGTTGAAGGTACAAGCTGATCTATATTTC  180
1728mer_15        GGTAATAGCCTGGATGAGTTTATGGCGTGGGTGTTGAAGGTACAAGCTGATCTATATTTC  180
1728mer_16        GGTAATAGCCTGGATGAGTTTATGGCGTGGGTGTTGAAGGTACAAGCTGATCTATATTTC  180
                  ************************************************ ***********

1728mer_Reference cataacctcaaatttgacggagcttttatcattaactggttggaacgtaatggttttaag  240
1728mer_1         CATAACCTCAAATTTGACGGAGCTTTTATCATTAACTGGTTGGAACGTAATGGTTTTAAG  240
1728mer_2         CATAACCTCAAATTTGACGGAGCTTTTATCATTAACTGGTTGGAACGTAATGGTTTTAAG  238
1728mer_3         CATAACCTCAAATTTGACGGAGCTTTTATCATTAACTGGTTGGAACGTAATGGTTTTAAG  240
1728mer_4         CATAACCTCAAATTTGACGGAGCTTTTATCATTAACTGGTTGGAACGTAATGGTTTTAAG  240
1728mer_5         CATAACCTCAAATTTGACGGAGCTTTTATCATTAACTGGTTGGAACGTAATGGTTTTAAG  240
1728mer_6         CATAACCTCAAATTTGACGGAGCTTTTATCATTAACTGGTTGGAACGTAATGGTTTTAAG  240
1728mer_7         CATAACCTCAAATTTGACGGAGCTTTTATCATTAACTGGTTGGAACGTAATGGTTTTAAG  240
1728mer_9         CATAACCTCAAATTTGACGGAGCTTTTATCATTAACTGGTTGGAACGTAATGGTTTTAAG  240
1728mer_8         CATAACCTCAAATTTGACGGAGCTTTTATCATTAACTGGTTGGAACGTAATGGTTTTAAG  240
1728mer_10        CATAACCTCAAATTTGACGGAGCTTTTATCATTAACTGGTTGGAACGTAATGGTTTTAAG  240
1728mer_11        CATAACCTCAAATTTGACGGAGCTTTTATCATTAACTGGTTGGAACGTAATGGTTTTAAG  240
1728mer_12        CATAACCTCAAATTTGACGGAGCTTTTATCATTAACTGGTTGGAACGTAATGGTTTTAAG  240
1728mer_13        CATAACCTCAAATTTGACGGAGCTTTTATCATTAACTGGTTGGAACGTAATGGTTTTAAG  240
1728mer_14        CATAACCTCAAATTTGACGGAGCTTTTATCATTAACTGGTTGGAACGTAATGGTTTTAAG  240
1728mer_15        CATAACCTCAAATTTGACGGAGCTTTTATCATTAACTGGTTGGAACGTAATGGTTTTAAG  240
1728mer_16        CATAACCTCAAATTTGACGGAGCTTTTATCATTAACTGGTTGGAACGTAATGGTTTTAAG  240
                  ************************************************************

1728mer_Reference tggtcggctgacggattgccaaacacatataatacgatcatatctcgcatgggacaatgg  300
1728mer_1         TGGTCGGCTGACGGATTGCCAAACACATATAATACGATCATATCTCGCATGGGACAATGG  300
1728mer_2         TGGTCGGCTGACGGATTGCCAAACACATATAATACGATCATATCTCGCATGGGACAATGG  298
1728mer_3         TGGTCGGCTGACGGATTGCCAAACACATATAATACGATCATATCTCGCATGGGACAATGG  300
1728mer_4         TGGTCGGCTGACGGATTGCCAAACACATATAATACGATCATATCTCGCATGGGACAATGG  300
1728mer_5         TGGTCGGCTGACGGATTGCCAAACACATATAATACGATCATATCTCGCATGGGACAATGG  300
1728mer_6         TGGTCGGCTGACGGATTGCCAAACACATATAATACGATCATATCTCGCAGG-GACAATGG  299
1728mer_7         TGGTCGGCTGACGGATTGCCAAACACATATAATACGATCATATCTCGCATGGGACAATGG  300
1728mer_9         TGGTCGGCTGACGGATTGCCAAACACATATAATACGATCATATCTCGCATGGGACAATGG  300
1728mer_8         TGGTCGGCTGACGGATTGCCAAACACATATAATACGATCATATCTCGCATGGGACAATGG  300
1728mer_10        TGGTCGGCTGACGGATTGCCAAACACATATAATACGATCATATCTCGCATGGGACAATGG  300
1728mer_11        TGGTCGGCTGACGGATTGCCAAACACATATAATACGATCATATCTCGCATGGGACAATGG  300
1728mer_12        TGGTCGGCTGACGGATTGCCAAACACATATAATACGATCATATCTCGCATGGGACAATGG  300
1728mer_13        TGGTCGGCTGACGGATTGCCAAACACATATAATACGATCATATCTCGCATGGGACAATGG  300
1728mer_14        TGGTCGGCTGACGGATTGCCAAACACATATAATACGATCATATCTCGCATGGGACAATGG  300
1728mer_15        TGGTCGGCTGACGGATTGCCAAACACATATAATACGATCATATCTCGCAT--GACAATGG  298
1728mer_16        TGGTCGGCTGACGGATTGCCAAACACATATAATACGATCATATCTCGCATGGGACAATGG  300
                  *************************************************   ********
```

```
1728mer_Reference tacatgattgatatatgtttaggctacaaagggaaacgtaagatacatacagtgatatat  360
1728mer_1         TACATGATTGATATATGTTTAGGCTACAAAGGGAAACGTAAGATACATACAGTGATATAT  360
1728mer_2         TACATGATTGATATATGTTTAGGCTACAAAGGGAAACGTAAGATACATACAGTGATATAT  358
1728mer_3         TACATGATTGATATATGTTTAGGCTACAAAGGGAAACGTAAGATACATACAGTGATATAT  360
1728mer_4         TACATGATTGATATATGTTTAGGCTACAAAGGGAAACGTAAGATACATACAGTGATATAT  360
1728mer_5         TACATGATTGATATATGTTTAGGCTACAAAGGGAAACGTAAGATACATACAGTGATATAT  360
1728mer_6         TACATGATTGATATATGTTTAGGCTACAAAGGGAAACGTAAGATACATACAGTGATATAT  359
1728mer_7         TACATGATTGATATATGTTTAGGCTACAAAGGGAAACGTAAGATACATACAGTGATATAT  360
1728mer_9         TACATGATTGATATATGTTTAGGCTACAAAGGGAAACGTAAGATACATACAGTGATATAT  360
1728mer_8         TACATGATTGATATATGTTTAGGCTACAAAGGGAAACGTAAGATACATACAGTGATATAT  360
1728mer_10        TACATGATTGATATATGTTTAGGCTACAAAGGGAAACGTAAGATACATACAGTGATATAT  360
1728mer_11        TACATGATTGATATATGTTTAGGCTACAAAGGGAAACGTAAGATACATACAGTGATATAT  360
1728mer_12        TACATGATTGATATATGTTTAGGCTACAAAGGGAAACGTAAGATACATACAGTGATATAT  360
1728mer_13        TACATGATTGATATATGTTTAGGCTACAAAGGGAAACGTAAGATACATACAGTGATATAT  360
1728mer_14        TACATGATTGATATATGTTTAGGCTACAAAGGGAAACGTAAGATACATACAGTGATATAT  360
1728mer_15        TACATGATTGATATATGTTTAGGCTACAAAGGGAAACGTAAGATACATACAGTGATATAT  358
1728mer_16        TACATGATTGATATATGTTTAGGCTACAAAGGGAAACGTAAGATACATACAGTGATATAT  360
                  ************************************************************

1728mer_Reference gacagcttaaagaaactaccgtttcctgttaagaagatagctaaagactttaaactaact  420
1728mer_1         GACAGCTTAAAGAAACTACCGTTTCCTGTTAAGAAGATAGCTAAAGACTTTAAACTAACT  420
1728mer_2         GACAGCTTAAAGAAACTACCGTTTCCTGTTAAGAAGATAGCTAAAGACTTTAAACTAACT  418
1728mer_3         GACAGCTTAAAGAAACTACCGTTTCCTGTTAAGAAGATAGCTAAAGACTTTAAACTAACT  420
1728mer_4         GACAGCTTAAAGAAACTACCGTTTCCTGTTAAG-AGATAGCTAAAGACTTTAAACTAACT  419
1728mer_5         GACAGCTTAAAGAAACTACCGTTTCCTGTTAAGAAGATAGCTAAAGACTTTAAACTAACT  420
1728mer_6         GACAGCTTAAAGAAACTACCGTTTCCTGTTAAGAAGATAGCTAAAGACTTTAAACTAACT  419
1728mer_7         GACAGCTTAAAGAAACTACCGTTTCCTGTTAAGAAGATAGCTAAAGACTTTAAACTAACT  420
1728mer_9         GACAGCTTAAAGAAACTACCGTTTCCTGTTAAGAAGATAGCTAAAGACTTTAAACTAACT  420
1728mer_8         GACAGCTTAAAGAAACTACCGTTTCCTGTTAAGAAGATAGCTAAAGACTTTAAACTAACT  420
1728mer_10        GACAGCTTAAAGAAACTACCGTTTCCTGTTAAGAAGATAGCTAAAGACTTTAAACTAACT  420
1728mer_11        GACAGCTTAAAGAAACTACCGTTTCCTGTTAAGAAGATAGCTAAAGACTTTAAACTAACT  420
1728mer_12        GACAGCTTAAAGAAACTACCGTTTCCTGTTAAGAAGATAGCTAAAGACTTTAAACTAACT  420
1728mer_13        GACAGCTTAAAGAAACTACCGTTTCCTGTTAAGAAGATAGCTAAAGACTTTAAACTAACT  420
1728mer_14        GACAGCTTAAAGAAACTACCGTTTCCTGTTAAGAAGATAGCTAAAGACTTTAAACTAACT  420
1728mer_15        GACAGCTTAAAGAAACTACCGTTTCCTGTTAAGAAGATAGCTAAAGACTTTAAACTAACT  418
1728mer_16        GACAGCTTAAAGAAACTACCGTTTCCTGTTAAGAAGATAGCTAAAGACTTTAAACTAACT  420
                  ********************************* **************************

1728mer_Reference gttcttaaaggtgatattgattaccacaaagaaagaccagtcggctataagataacaccc  480
1728mer_1         GTTCTTAAAGGTGATATTGATTACCACAAAGAAAGACCAGTCGGCTATAAGATAACACCC  480
1728mer_2         GTTCTTAAAGGTGATATTGATTACCACAAAGAAAGACCAGTCGGCTATAAGATAACACCC  478
1728mer_3         GTTCTTAAAGGTGATATTGATTACCACAAAGAAAGACCAGTCGGCTATAAGATAACACCC  480
1728mer_4         GTTCTTAAAGGTGATATTGATTACCACAAAGAAAGACCAGTCGGCTATAAGATAACACCC  479
1728mer_5         GTTCTTAAAGGTGATATTGATTACCACAAAGAAAGACCAGTCGGCTATAAGATAACACCC  480
1728mer_6         GTTCTTAAAGGTGATATTGATTACCACAAAGAAAGACCAGTCGGCTATAAGATAACACCC  479
1728mer_7         GTTCTTAAAGGTGATATTGATTACCACAAAGAAAGACCAGTCGGCTATAAGATAACACCC  480
1728mer_9         GTTCTTAAAGGTGATATTGATTACCACAAAGAAAGACCAGTCGGCTATAAGATAACACCC  480
1728mer_8         GTTCTTAAAGGTGATATTGATTACCACAAAGAAAGACCAGTCGGCTATAAGATAACACCC  480
1728mer_10        GTTCTTAAAGGTGATATTGATTACCACAAAGAAAGACCAGTCGGCTATAAGATAACACCC  480
1728mer_11        GTTCTTAAAGGTGATATTGATTACCACAAAGAAAGACCAGTCGGCTATAAGATAACACCC  480
```

```
1728mer_12        GTTCTTAAAGGTGATATTGATTACCACAAAGAAAGACCAGTCGGCTATAAGATAACACCC  480
1728mer_13        GTTCTTAAAGGTGATATTGATTACCACAAAGAAAGACCAGTCGGCTATAAGATAACACCC  480
1728mer_14        GTTCTTAAAGGTGATATTGATTACCACAAAGAAAGACCAGTCGGCTATAAGATAACACCC  480
1728mer_15        GTTCTTAAAGGTGATATTGATTACCACAAAGAAAGACCAGTCGGCTATAAGATAACACCC  478
1728mer_16        GTTCTTAAAGGTGATATTGATTACCACAAAGAAAGACCAGTCGGCTATAAGATAACACCC  480
                  ************************************************************

1728mer_Reference gaagaatacgcctatattaaaaacgatattcagattattgcggaacgtctgttaattcag  540
1728mer_1         GAAGAATACGCCTATATTAAAAACGATATTCAGATTATTGCGGAACGTCTGTTAATTCAG  540
1728mer_2         GAAGAATACGCCTATATTAAAAACGATATTCAGATTATTGCGGAACGTCTGTTAATTCAG  538
1728mer_3         GAAGAATACGCCTATATTAAAAACGATATTCAGATTATTGCGGAACGTCTGTTAATTCAG  540
1728mer_4         GAAGAATACGCCTATATTAAAAACGATATTCAGATTGTTGCGGAACGTCTGTTAATTCAG  539
1728mer_5         GAAGAATACGCCTATATTAAAAACGATATTCAGATTATTGCGGAACGTCTGTTAATTCAG  540
1728mer_6         GAAGAATACGCCTATATTAAAAACGATATTCAGATTATTGCGGAACGTCTGTTAATTCAG  539
1728mer_7         GAAGAATACGCCTATATTAAAAACGATATTCAGATTATTGCGGAACGTCTGTTAATTCAG  540
1728mer_9         GAAGAATACGCCTATATTAAAAACGATATTCAGATTATTGCGGAACGTCTGTTAATTCAG  540
1728mer_8         GAAGAATACGCCTATATTAAAAACGATATTCAGATTATTGCGGAACGTCTGTTAATTCAG  540
1728mer_10        GAAGAATACGCCTATATTAAAAACGATATTCAGATTATTGCGGAACGTCTGTTAATTCAG  540
1728mer_11        GAAGAATACGCCTATATTAAAAACGATATTCAGATTATTGCGGAACGTCTGTTAATTCAG  540
1728mer_12        GAAGAATACGCCTATATTAAAAACGATATTCAGATTATTGCGGAACGTCTGTTAATTCAG  540
1728mer_13        GAAGAATACGCCTATATTAAAAACGATATTCAGATTATTGCGGAACGTCTGTTAATTCAG  540
1728mer_14        GAAGAATACGCCTATATTAAAAACGATATTCAGATTATTGCGGAACGTCTGTTAATTCAG  540
1728mer_15        GAAGAATACGCCTATATTAAAAACGATATTCAGATTATTGCGGAACGTCTGTTAATTCAG  538
1728mer_16        GAAGAATACGCCTATATTAAAAACGATATTCAGATTATTGCGGAACGTCTGTTAATTCAG  540
                  ************************************ ***********************

1728mer_Reference tttaagcaag-gtttagaccggatgacagcaggcagtgacagtctaaaaggtttcaagga  599
1728mer_1         TTTAAGCAAG-GTTTAGACCGGATGACAGCAGGCAGTGACAGTCTAAAAGGTTTCAAGGA  599
1728mer_2         TTTAAGCAAG-GTTTAGACCGGATGACAGCAGGCAGTGACAGTCTAAAAGGTTTCAAGGA  597
1728mer_3         TTTAAGCAAGGGTTTAGACCGGATGACAGCAGGCAGTGACAGTCTAAAAGGTTTCAAGGA  600
1728mer_4         TTTAAGCAAG-GTTTAGACCTGATGACAGCAGGCAGTGACAGTCTAAAAGGTTTCAAGGA  598
1728mer_5         TTTAAGCAAG-GTTTAGACCGGATGACAGCAGGCAGTGACAGTCTAAAAGGTTTCAAGGA  599
1728mer_6         TTTAAGCAAG-GTTTAGACCGGATGACAGCAGGCAGTGACAGTCTAAAAGGTTTCAAGGA  598
1728mer_7         TTTAAGCAAG-GTTTAGACCGGATGACAGCAGGCAGTGACAGTCTAAAAGGTTTCAAGGA  599
1728mer_9         TTTAAGCAAG-GTTTAGACCGGATGACAGCAGGCAGTGACAGTCTAAAAGGTTTCAAGGA  599
1728mer_8         TTTAAGCAAG-GTTTAGACCGGATGACAGCAGGCAGTGACAGTCTAAAAGGTTTCAAGGA  599
1728mer_10        TTTAAGCAAG-GTTTAGACCGGATGACAGCAGGCAGTGACAGTCTAAAAGGTTTCAAGGA  599
1728mer_11        TTTAAGCAAG-GTTTAGACCGGATGACAGCAGGCAGTGACAGTCTAAAAGGTTTCAAGGA  599
1728mer_12        TTTAAGCAAG-GTTTAGACCGGATGACAGCAGGCAGTGACAGTCTAAAAGGTTTCAAGGA  599
1728mer_13        TTTAAGCAAG-GTTTAGACCGGATGACAGCAGGCAGTGACAGTCTAAAAGGTTTCAAGGA  599
1728mer_14        TTTAAGCAAG-GTTTAGACCGGATGACAGCAGGCAGTGACAGTCTAAAAGGTTTCAAGGA  599
1728mer_15        TTTAAGCAAG-GTTTAGACCGGATGACAGCAGGCAGTGACAGTCTAAAAGGTTTCAAGGA  597
1728mer_16        TTTAAGCAAG-GTTTAGACCGGATGACAGCAGGCAGTGACAGTCTAAAAGGTTTCAAGGA  599
                  ********** ********* ***************************************

1728mer_Reference tattataaccactaagaaattcaaaaaggtgtttcctacattgagtcttggactcgataa  659
1728mer_1         TATTATAACCACTAAGAAATTCAAAAAGGTGTTTCCTACATTGAGTCTTGGACTCGATAA  659
1728mer_2         TATTATAACCACTAAGAAATTCAAAAAGGTGTTTCCTACATTGAGTCTTGGACTCGATAA  657
1728mer_3         TATTATAACCACTAAGAAATTCAAAAAGGTGTTTCCTACATTGAGTCTTGGACTCGATAA  660
1728mer_4         TATTATAACCACTAAGAAATTCAAAAAGGTGTTTCCTACATTGAGTCTTGGACTCGATAA  658
```

```
1728mer_5          TATTATAACCACTAAGAAATTCAAAAAGGTGTTTCCTACATTGAGTCTTGGACTCGATAA  659
1728mer_6          TATTATAACCACTAAGAAATTCAAAAAGGTGTTTCCTACATTGAGTCTTGGACTCGATAA  658
1728mer_7          TATTATAACCACTAAGAAATTCAAAAAGGTGTTTCCTACATTGAGTCTTGGACTCGATAA  659
1728mer_9          TATTATAACCACTAAGAAATTCAAAAAGGTGTTTCCTACATTGAGTCTTGGACTCGATAA  659
1728mer_8          TATTATAACCACTAAGAAATTCAAAAAGGTGTTTCCTACATTGAGTCTTGGACTCGATAA  659
1728mer_10         TATTATAACCACTAAGAAATTCAAAAAGGTGTTTCCTACATTGAGTCTTGGACTCGATAA  659
1728mer_11         TATTATAACCACTAAGAAATTCAAAAAGGTGTTTCCTACATTGAGTCTTGGACTCGATAA  659
1728mer_12         TATTATAACCACTAAGAAATTCAAAAAGGTGTTTCCTACATTGAGTCTTGGACTCGATAA  659
1728mer_13         TATTATAACCACTAAGAAATTCAAAAAGGTGTTTCCTACATTGAGTCTTGGACTCGATAA  659
1728mer_14         TATTATAACCACTAAGAAATTCAAAAAGGTGTTTCCTACATTGAGTCTTGGACTCGATAA  659
1728mer_15         TATTATAACCACTAAGAAATTCAAAAAGGTGTTTCCTACATTGAGTCTTGGACTCGATAA  657
1728mer_16         TATTATAACCACTAAGAAATTCAAAAAGGTGTTTCCTACATTGAGTCTTGGACTCGATAA  659
                   ************************************************************

1728mer_Reference  ggaagtgagatacgcctatagagg-tggttttacatggttaaatgataggttcaaagaaa  718
1728mer_1          GGAAGTGAGATACGCCTATAGAGG-TGGTTTTACATGGTTAAATGATAGGTTCAAAGAAA  718
1728mer_2          GGAAGTGAGATACGCCTATAGAGG-TGGTTTTACATGGTTAAATGATAGGTTCAAAGAAA  716
1728mer_3          GGAAGTGAGATACGCCTATAGAGG-TGGTTTTACATGGTTAAATGATAGGTTCAAAGAAA  719
1728mer_4          GGAAGTGAGATACGCCTATAGAGG-TGGTTTTACATGGTTAAATGATAGGTTCAAAGAAA  717
1728mer_5          GGAAGTGAGATACGCCTATAGAGG-TGGTTTTACATGGTTAAATGATAGGTTCAAAGAAA  718
1728mer_6          GGAAGTGAGATACGCCTATAGAGG-TGGTTTTACATGGTTAAATGATAGGTTCAAAGAAA  717
1728mer_7          GGAAGTGAGATACGCCTATAGAGG-TGGTTTTACATGGTTAAATGATAGGTTCAAAGAAA  718
1728mer_9          GGAAGTGAGATACGCCTATAGAGG-TGGTTTTACATGGTTAAATGATAGGTTCAAAGAAA  718
1728mer_8          GGAAGTGAGATACGCCTATAGAGG-TGGTTTTACATGGTTAAATGATAGGTTCAAAGAAA  718
1728mer_10         GGAAGTGAGATACGCCTATAGAGG-TGGTTTTACATGGTTAAATGATAGGTTCAAAGAAA  718
1728mer_11         GGAAGTGAGATACGCCTATAGAGG-TGGTTTTACATGGTTAAATGATAGGTTCAAAGAAA  718
1728mer_12         GGAAGTGAGATACGCCTATAGAGG-TGGTTTTACATGGTTAAATGATAGGTTCAAAGAAA  718
1728mer_13         GGAAGTGAGATACGCCTATAGAGG-TGGTTTTACATGGTTAAATGATAGGTTCAAAGAAA  718
1728mer_14         GGAAGTGAGATACGCCTATAGAGG-TGGTTTTACATGGTTAAATGATAGGTTCAAAGAAA  718
1728mer_15         GGAAGTGAGATACGCCTATAGAGG-TGGTTTTACATGGTTAAATGATAGGTTCAAAGAAA  716
1728mer_16         GGAAGTGAGATACGCCTATAGAGGGTGGTTTTACATGGTTAAATGATAGGTTCAAAGAAA  719
                   ************************ ***********************************

1728mer_Reference  aagaaatcggagaaggcatggtcttcgatgttaatagtctatatcctgcacagatgtata  778
1728mer_1          AAGAAATCGGAGAAGGCATGGTCTTCGATGTTAATAGTCTATATCCTGCACAGATGTATA  778
1728mer_2          AAGAAATCGGAGAAGGCATGGTCTTCGATGTTAATAGTCTATATCCTGCACAGATGTATA  776
1728mer_3          AAGAAATCGGAGAAGGCATGGTCTTCGATGTTAATAGTCTATATCCTGCACAGATGTATA  779
1728mer_4          AAGAAATCGGAGAAGGCATGGTCTTCGATGTTAATAGTCTATATCCTGCACAGATGTATA  777
1728mer_5          AAGAAATCGGAGAAGGCATGGTCTTCGATGTTAATAGTCTATATCCTGCACAGATGTATA  778
1728mer_6          AAGAAATCGGAGAAGGCATGGTCTTCGATGTTAATAGTCTATATCCTGCACAGATGTATA  777
1728mer_7          AAGAAATCGGAGAAGGCATGGTCTTCGATGTTAATAGTCTATATCCTGCACAGATGTATA  778
1728mer_9          AAGAAATCGGAGAAGGCATGGTCTTCGATGTTAATAGTCTATATCCTGCACAGATGTATA  778
1728mer_8          AAGAAATCGGAGAAGGCATGGTCTTCGATGTTAATAGTCTATATCCTGCACAGATGTATA  778
1728mer_10         AAGAAATCGGAGAAGGCATGGTCTTCGATGTTAATAGTCTATATCCTGCACAGATGTATA  778
1728mer_11         AAGAAATCGGAGAAGGCATGGTCTTCGATGTTAATAGTCTATATCCTGCACAGATGTATA  778
1728mer_12         AAGAAATCGGAGAAGGCATGGTCTTCGATGTTAATAGTCTATATCCTGCACAGATGTATA  778
1728mer_13         AAGAAATCGGAGAAGGCATGGTCTTCGATGTTAATAGTCTATATCCTGCACAGATGTATA  778
1728mer_14         AAGAAATCGGAGAAGGCATGGTCTTCGATGTTAATAGTCTATATCCTGCACAGATGTATA  778
1728mer_15         AAGAAATCGGAGAAGGCATGGTCTTCGATGTTAATAGTCTATATCCTGCACAGATGTATA  776
1728mer_16         AAGAAATCGGAGAAGGCATGGTCTTCGATGTTAATAGTCTATATCCTGCACAGATGTATA  779
```

```
                   ************************************************************

1728mer_Reference gccgtctccttccatatggtgaacctatagtattcgagggtaaatacgtttgggacgaag  838
1728mer_1         GCCGTCTCCTTCCATATGGTGAACCTATAGTATTCGAGGGTAAATACGTTTGGGACGAAG  838
1728mer_2         GCCGTCTCCTTCCATATGGTGAACCTATAGTATTCGAGGGTAAATACGTTTGGGACGAAG  836
1728mer_3         GCCGTCTCCTTCCATATGGTGAACCTATAGTATTCGAGGGTAAATACGTTTGGGACGAAG  839
1728mer_4         GCCGTCTCCTTCCATATGGTGAACCTATAGTATTCGAGGGTAAATACGTTTGGGACGAAG  837
1728mer_5         GCCGTCTCCTTCCATATGGTGAACCTATAGTATTCGAGGGTAAATACGTTTGGGACGAAG  838
1728mer_6         GCCGTCTCCTTCCATATGGTGAACCTATAGTATTCGAGGGTAAATACGTTTGGGACGAAG  837
1728mer_7         GCCGTCTCCTTCCATATGGTGAACCTATAGTATTCGAGGGTAAATACGTTTGGGACGAAG  838
1728mer_9         GCCGTCTCCTTCCATATGGTGAACCTATAGTATTCGAGGGTAAATACGTTTGGGACGAAG  838
1728mer_8         GCCGTCTCCTTCCATATGGTGAACCTATAGTATTCGAGGGTAAATACGTTTGGGACGAAG  838
1728mer_10        GCCGTCTCCTTCCATATGGTGAACCTATAGTATTCGAGGGTAAATACGTTTGGGACGAAG  838
1728mer_11        GCCGTCTCCTTCCATATGGTGAACCTATAGTATTCGAGGGTAAATACGTTTGGGACGAAG  838
1728mer_12        GCCGTCTCCTTCCATATGGTGAACCTATAGTATTCGAGGGTAAATACGTTTGGGACGAAG  838
1728mer_13        GCCGTCTCCTTCCATATGGTGAACCTATAGTATTCGAGGGTAAATACGTTTGGGACGAAG  838
1728mer_14        GCCGTCTCCTTCCATATGGTGAACCTATAGTATTCGAGGGTAAATACGTTTGGGACGAAG  838
1728mer_15        GCCGTCTCCTTCCATATGGTGAACCTATAGTATTCGAGGGTAAATACGTTTGGGACGAAG  836
1728mer_16        GCCGTCTCCTTCCATATGGTGAACCTATAGTATTCGAGGGTAAATACGTTTGGGACGAAG  839
                  ************************************************************

1728mer_Reference attacccactacacatacagcatatcagatgtgagttcgaattgaaagagggctatatac  898
1728mer_1         ATTACCCACTACACATACAGCATATCAGATGTGAGTTCGAATTGAAAGAGGGCTATATAC  898
1728mer_2         ATTACCCACTACACATACAGCATATCAGATGTGAGTTCGAATTGAAAGAGGGCTATATAC  896
1728mer_3         ATTACCCACTACACATACAGCATATCAGATGTGAGTTCGAATTGAAAGAGGGCTATATAC  899
1728mer_4         ATTACCCACTACACATACAGCATATCAGATGTGAGTTCGAATTGAAAGAGGGCTATATAC  897
1728mer_5         ATTACCCACTACACATACAGCATATCAGATGTGAGTTCGAATTGAAAGAGGGCTATATAC  898
1728mer_6         ATTACCCACTACACATACAGCATATCAGATGTGAGTTCGAATTGAAAGAGGGCTATATAC  897
1728mer_7         ATTACCCACTACACATACAGCATATCAGATGTGAGTTCGAATTGAAAGAGGGCTATATAC  898
1728mer_9         ATTACCCACTACACATACAGCATATCAGATGTGAGTTCGAATTGAAAGAGGGCTATATAC  898
1728mer_8         ATTACCCACTACACATACAGCATATCAGATGTGAGTTCGAATTGAAAGAGGGCTATATAC  898
1728mer_10        ATTACCCACTACACATACAGCATATCAGATGTGAGTTCGAATTGAAAGAGGGCTATATAC  898
1728mer_11        ATTACCCACTACACATACAGCATATCAGATGTGAGTTCGAATTGAAAGAGGGCTATATAC  898
1728mer_12        ATTACCCACTACACATACAGCATATCAGATGTGAGTTCGAATTGAAAGAGGGCTATATAC  898
1728mer_13        ATTACCCACTACACATACAGCATATCAGATGTGAGTTCGAATTGAAAGAGGGCTATATAC  898
1728mer_14        ATTACCCACTACACATACAGCATATCAGATGTGAGTTCGAATTGAAAGAGGGCTATATAC  898
1728mer_15        ATTACCCACTACACATACAGCATATCAGATGTGAGTTCGAATTGAAAGAGGGCTATATAC  896
1728mer_16        ATTACCCACTACACATACAGCATATCAGATGTGAGTTCGAATTGAAAGAGGGCTATATAC  899
                  ************************************************************

1728mer_Reference ccactatacagataaaaagaagtaggttttataaaggtaatgagtacctaaaaagtagcg  958
1728mer_1         CCACTATACAGATAAAAAGAAGTAGGTTTTATAAAGGTAATGAGTACCTAAAAAGTAGCG  958
1728mer_2         CCACTATACAGATAAAAAGAAGTAGGTTTTATAAAGGTAATGAGTACCTAAAAAGTAGCG  956
1728mer_3         CCACTATACAGATAAAAAGAAGTAGGTTTTATAAAGGTAATGAGTACCTAAAAAGTAGCG  959
1728mer_4         CCACTATACAGATAAAAAGAAGTAGGTTTTATAAAGGTAATGAGTACCTAAAAAGTAGCG  957
1728mer_5         CCACTATACAGATAAAAAGAAGTAGGTTTTATAAAGGTAATGAGTACCTAAAAAGTAGCG  958
1728mer_6         CCACTATACAGATAAAAAGAAGTAGGTTTTATAAAGGTAATGAGTACCTAAAAAGTAGCG  957
1728mer_7         CCACTATACAGATAAAAAGAAGTAGGTTTTATAAAGGTAATGAGTACCTAAAAAGTAGCG  958
1728mer_9         CCACTATACAGATAAAAAGAAGTAGGTTTTATAAAGGTAATGAGTACCTAAAAAGTAGCG  958
1728mer_8         CCACTATACAGATAAAAAGAAGTAGGTTTTATAAAGGTAATGAGTACCTAAAAAGTAGCG  958
```

```
1728mer_10        CCACTATACAGATAAAAAGAAGTAGGTTTTATAAAGGTAATGAGTACCTAAAAAGTAGCG  958
1728mer_11        CCACTATACAGATAAAAAGAAGTAGGTTTTATAAAGGTAATGAGTACCTAAAAAGTAGCG  958
1728mer_12        CCACTATACAGATAAAAAGAAGTAGGTTTTATAAAGGTAATGAGTACCTAAAAAGTAGCG  958
1728mer_13        CCACTATACAGATAAAAAGAAGTAGGTTTTATAAAGGTAATGAGTACCTAAAAAGTAGCG  958
1728mer_14        CCACTATACAGATAAAAAGAAGTAGGTTTTATAAAGGTAATGAGTACCTAAAAAGTAGCG  958
1728mer_15        CCACTATACAGATAAAAAGAAGTAGGTTTTATAAAGGTAATGAGTACCTAAAAAGTAGCG  956
1728mer_16        CCACTATACAGATAAAAAGAAGTAGGTTTTATAAAGGTAATGAGTACCTAAAAAGTAGCG  959
                  ************************************************************

1728mer_Reference gcggggagatagccgacctctggttgtcaaatgtagacctagaattaatgaaagaacact  1018
1728mer_1         GCGGGGAGATAGCCGACCTCTGGTTGTCAAATGTAGACCTAGAATTAATGAAAGAACACT  1018
1728mer_2         GCGGGGAGATAGCCGACCTCTGGTTGTCAAATGTAGACCTAGAATTAATGAAAGAACACT  1016
1728mer_3         GCGGGGAGATAGCCGACCTCTGGTTGTCAAATGTAGACCTAGAATTAATGAAAGAACACT  1019
1728mer_4         GCGGGGAGATAGCCGACCTCTGGTTGTCAAATGTAGACCTAGAATTAATGAAAGAACACT  1017
1728mer_5         GCGGGGAGATAGCCGACCTCTGGTTGTCAAATGTAGACCTAGAATTAATGAAAGAACACT  1018
1728mer_6         GCGGGGAGATAGCCGACCTCTGGTTGTCAAATGTAGACCTAGAATTAATGAAAGAACACT  1017
1728mer_7         GCGGGGAGATAGCCGACCTCTGGTTGTCAAATGTAGACCTAGAATTAATGAAAGAACACT  1018
1728mer_9         GCGGGGAGATAGCCGACCTCTGGTTGTCAAATGTAGACCTAGAATTAATGAAAGAACACT  1018
1728mer_8         GCGGGGAGATAGCCGACCTCTGGTTGTCAAATGTAGACCTAGAATTAATGAAAGAACACT  1018
1728mer_10        GCGGGGAGATAGCCGACCTCTGGTTGTCAAATGTAGACCTAGAATTAATGAAAGAACACT  1018
1728mer_11        GCGGGGAGATAGCCGACCTCTGGTTGTCAAATGTAGACCTAGAATTAATGAAAGAACACT  1018
1728mer_12        GCGGGGAGATAGCCGACCTCTGGTTGTCAAATGTAGACCTAGAATTAATGAAAGAACACT  1018
1728mer_13        GCGGGGAGATAGCCGACCTCTGGTTGTCAAATGTAGACCTAGAATTAATGAAAGAACACT  1018
1728mer_14        GCGGGGAGATAGCCGACCTCTGGTTGTCAAATGTAGACCTAGAATTAATGAAAGAACACT  1018
1728mer_15        GCGGGGAGATAGCCGACCTCTGGTTGTCAAATGTAGACCTAGAATTAATGAAAGAACACT  1016
1728mer_16        GCGGGGAGATAGCCGACCTCTGGTTGTCAAATGTAGACCTAGAATTAATGAAAGAACACT  1019
                  ************************************************************

1728mer_Reference acgatttatataacgttgaatatatcagcggcttaaaatttaaagtaactacaggtttgt  1078
1728mer_1         ACGATTTATATAACGTTGAATATATCAGCGGCTTAAAATTTAAAGTAACTACAGGTTTGT  1078
1728mer_2         ACGATTTATATAACGTTGAATATATCAGCGGCTTAAAATTTAAAGTAACTACAGGTTTGT  1076
1728mer_3         ACGATTTATATAACGTTGAATATATCAGCGGCTTAAAATTTAAAGTAACTACAGGTTTGT  1079
1728mer_4         ACGATTTATATAACGTTGAATATATCAGCGGCTTAAAATTTAAAGTAACTACAGGTTTGT  1077
1728mer_5         ACGATTTATATAACGTTGAATATATCAGCGGCTTAAAATTTAAAGTAACTACAGGTTTGT  1078
1728mer_6         ACGATTTATATAACGTTGAATATATCAGCGGCTTAAAATTTAAAGTAACTACAGGTTTGT  1077
1728mer_7         ACGATTTATATAACGTTGAATATATCAGCGGCTTAAAATTTAAAGTAACTACAGGTTTGT  1078
1728mer_9         ACGATTTATATAACGTTGAATATATCAGCGGCTTAAAATTTAAAGTAACTACAGGTTTGT  1078
1728mer_8         ACGATTTATATAACGTTGAATATATCAGCGGCTTAAAATTTAAAGTAACTACAGGTTTGT  1078
1728mer_10        ACGATTTATATAACGTTGAATATATCAGCGGCTTAAAATTTAAAGTAACTACAGGTTTGT  1078
1728mer_11        ACGATTTATATAACGTTGAATATATCAGCGGCTTAAAATTTAAAGTAACTACAGGTTTGT  1078
1728mer_12        ACGATTTATATAACGTTGAATATATCAGCGGCTTAAAATTTAAAGTAACTACAGGTTTGT  1078
1728mer_13        ACGATTTATATAACGTTGAATATATCAGCGGCTTAAAATTTAAAGTAACTACAGGTTTGT  1078
1728mer_14        ACGATTTATATAACGTTGAATATATCAGCGGCTTAAAATTTAAAGTAACTACAGGTTTGT  1078
1728mer_15        ACGATTTATATAACGTTGAATATATCAGCGGCTTAAAATTTAAAGTAACTACAGGTTTGT  1076
1728mer_16        ACGATTTATATAACGTTGAATATATCAGCGGCTTAAAATTTAAAGTAACTACAGGTTTGT  1079
                  ************************************************************

1728mer_Reference ttaaagattttatagataaatggacgtacatcaagacgacatcagaaggagcgatcaagc  1138
1728mer_1         TTAAAGATTTTATAGATAAATGGACGTACATCAAGACGACATCAGAAGGAGCGATCAAGC  1138
1728mer_2         TTAAAGATTTTATAGATAAATGGACGTACATCAAGACGACATCAGAAGGAGCGATCAAGC  1136
```

```
1728mer_3          TTAAAGATTTTATAGATAAATGGACGTACATCAAGACGACATCAGAAGGAGCGATCAAGC  1139
1728mer_4          TTAAAGATTTTATAGATAAATGGACGTACATCAAGACGACATCAGAAGGAGCGATCAAGC  1137
1728mer_5          TTAAAGATTTTATAGATAAATGGACGTACATCAAGACGACATCAGAAGGAGCGATCAAGC  1138
1728mer_6          TTAAAGATTTTATAGATAAATGGACGTACATCAAGACGACATCAGAAGGAGCGATCAAGC  1137
1728mer_7          TTAAAGATTTTATAGATAAATGGACGTACATCAAGACGACATCAGAAGGAGCGATCAAGC  1138
1728mer_9          TTAAAGATTTTATAGATAAATGGACGTACATCAAGACGACATCAGAAGGAGCGATCAAGC  1138
1728mer_8          TTAAAGATTTTATAGATAAATGGACGTACATCAAGACTACATCAGAAGGAGCGATCAAGC  1138
1728mer_10         TTAAAGATTTTATAGATAAATGGACGTACATCAAGACGACATCAGAAGGAGCGATCAAGC  1138
1728mer_11         TTAAAGATTTTATAGATAAATGGACGTACATCAAGACGACATCAGAAGGAGCGATCAAGC  1138
1728mer_12         TTAAAGATTTTATAGATAAATGGACGTACATCAAGACGACATCAGAAGGAGCGATCAAGC  1138
1728mer_13         TTAAAGATTTTATAGATAAATGGACGTACATCAAGACGACATCAGAAGGAGCGATCAAGC  1138
1728mer_14         TTAAAGATTTTATAGATAAATGGACGTACATCAAGACGACATCAGAAGGAGCGATCAAGC  1138
1728mer_15         TTAAAGATTTTATAGATAAATGGACGTACATCAAGACGACATCAGAAGGAGCGATCAAGC  1136
1728mer_16         TTAAAGATTTTATAGATAAATGGACGTACATCAAGACGACATCAGAAGGAGCGATCAAGC  1139
                   ************************************* **********************

1728mer_Reference  aactagcaaaactgatgttaaacagtctatacggtaaattcgctagtaaccctgatgtta  1198
1728mer_1          AACTAGCAAAACTGATGTTAAACAGTCTATACGGTAAATTCGCTAGTAACCCTGATGTTA  1198
1728mer_2          AACTAGCAAAACTGATGTTAAACAGTCTATACGGTAAATTCGCTAGTAACCCTGATGTTA  1196
1728mer_3          AACTAGCAAAACTGATGTTAAACAGTCTATACGGTAAATTCGCTAGTAACCCTGATGTTA  1199
1728mer_4          AACTAGCAAAACTGATGTTAAACAGTCTATACGGTAAATTCGCTAGTAACCCTGATGTTA  1197
1728mer_5          AACTAGCAAAACTGATGTTAAACAGTCTATACGGTAAATTCGCTAGTAACCCTGATGTTA  1198
1728mer_6          AACTAGCAAAACTGATGTTAAACAGTCTATACGGTAAATTCGCTAGTAACCCTGATGTTA  1197
1728mer_7          AACTAGCAAAACTGATGTTAAACAGTCTATACGGTAAATTCGCTAGTAACCCTGATGTTA  1198
1728mer_9          AACTAGCAAAACTGATGTTAAACAGTCTATACGGTAAATTCGCTAGTAACCCTGATGTTA  1198
1728mer_8          AACTAGCAAAACTGATGTTAAACAGTCTATACGGTAAATTCGCTAGTAACCCTGATGTTA  1198
1728mer_10         AACTAGCAAAACTGATGTTAAACAGTCTATACGGTAAATTCGCTAGTAACCCTGATGTTA  1198
1728mer_11         AACTAGCAAAACTGATGTTAAACAGTCTATACGGTAAATTCGCTAGTAACCCTGATGTTA  1198
1728mer_12         AACTAGCAAAACTGATGTTAAACAGTCTATACGGTAAATTCGCTAGTAACCCTGATGTTA  1198
1728mer_13         AACTAGCAAAACTGATGTTAAACAGTCTATACGGTAAATTCGCTAGTAACCCTGATGTTA  1198
1728mer_14         AACTAGCAAAACTGATGTTAAACAGTCTATACGGTAAATTCGCTAGTAACCCTGATGTTA  1198
1728mer_15         AACTAGCAAAACTGATGTTAAACAGTCTATACGGTAAATTCGCTAGTAACCCTGATGTTA  1196
1728mer_16         AACTAGCAAAACTGATGTTAAACAGTCTATACGGTAAATTCGCTAGTAACCCTGATGTTA  1199
                   ************************************************************

1728mer_Reference  cagggaaagtcccttatttaaaagagaatggggcgctaggtttcagacttggagaagagg  1258
1728mer_1          CAGGGAAAGTCCCTTATTTAAAAGAGAATGGGGCGCTAGGTTTCAGACTTGGAGAAGAGG  1258
1728mer_2          CAGGGAAAGTCCCTTATTTAAAAGAGAATGGGGCGCTAGGTTTCAGACTTGGAGAAGAGG  1256
1728mer_3          CAGGGAAAGTCCCTTATTTAAAAGAGAATGGGGCGCTAGGTTTCAGACTTGGAGAAGAGG  1259
1728mer_4          CAGGGAAAGTCCCTTATTTAAAAGAGAATGGGGCGCTAGGTTTCAGACTTGGAGAAGAGG  1257
1728mer_5          CAGGGAAAGTCCCTTATTTAAAAGAGAATGGGGCGCTAGGTTTCAGACTTGGAGAAGAGG  1258
1728mer_6          CAGGGAAAGTCCCTTATTTAAAAGAGAATGGGGCGCTAGGTTTCAGACTTGGAGAAGAGG  1257
1728mer_7          CAGGGAAAGTCCCTTATTTAAAAGAGAATGGGGCGCTAGGTTTCAGACTTGGAGAAGAGG  1258
1728mer_9          CAGGGAAAGTCCCTTATTTAAAAGAGAATGGGGCGCTAGGTTTCAGACTTGGAGAAGAGG  1258
1728mer_8          CAGGGAAAGTCCCTTATTTAAAAGAGAATGGGGCGCTAGGTTTCAGACTTGGAGAAGAGG  1258
1728mer_10         CAGGGAAAGTCCCTTATTTAAAAGAGAATGGGGCGCTAGGTTTCAGACTTGGAGAAGAGG  1258
1728mer_11         CAGGGAAAGTCCCTTATTTAAAAGAGAATGGGGCGCTAGGTTTCAGACTTGGAGAAGAGG  1258
1728mer_12         CAGGGAAAGTCCCTTATTTAAAAGAGAATGGGGCGCTAGGTTTCAGACTTGGAGAAGAGG  1258
1728mer_13         CAGGGAAAGTCCCTTATTTAAAAGAGAATGGGGCGCTAGGTTTCAGACTTGGAGAAGAGG  1258
1728mer_14         CAGGGAAAGTCCCTTATTTAAAAGAGAATGGGGCGCTAGGTTTCAGACTTGGAGAAGAGG  1258
```

```
1728mer_15         CAGGGAAAGTCCCTTATTTAAAAGAGAATGGGGCGCTAGGTTTCAGACTTGGAGAAGAGG  1256
1728mer_16         CAGGGAAAGTCCCTTATTTAAAAGAGAATGGGGCGCTAGGTTTCAGACTTGGAGAAGAGG  1259
                   ************************************************************

1728mer_Reference  aaacaaaagaccctgtttatacacctatgggcgttttcatcactgcatgggctagataca  1318
1728mer_1          AAACAAAAGACCCTGTTTATACACCTATGGGCGTTTTCATCACTGCATGGGCTAGATACA  1318
1728mer_2          AAACAAAAGACCCTGTTTATACACCTATGGGCGTTTTCATCACTGCATGGGCTAG-TACA  1315
1728mer_3          AAACAAAAGACCCTGTTTATACACCTATGGGCGTTTTCATCACTGCATGGGCTAGATACA  1319
1728mer_4          AAACAAAAGACCCTGTTTATACACCTATGGGCGTTTTCATCACTGCATGGGCTAGATACA  1317
1728mer_5          AAACAAAAGACCCTGTTTATACACCTATGGGCGTTTTCATCACTGCATGGGCTAGATACA  1318
1728mer_6          AAACAAAAGACCCTGTTTATACACCTATGGGCGTTTTCATCACTGCATGGGCTAGATACA  1317
1728mer_7          AAACAAAAGACCCTGTTTATACACCTATGGGCGTTTTCATCACTGCATGGGCTAGATACA  1318
1728mer_9          AAACAAAAGACCCTGTTTATACACCTATGGGCGTTTTCATCACTGCATGGGCTAGATACA  1318
1728mer_8          AAACAAAAGACCCTGTTTATACACCTATGGGCGTTTTCATCACTGCATGGGCTAGATACA  1318
1728mer_10         AAACAAAAGACCCTGTTTATACACCTATGGGCGTTTTCATCACTGCATGGGCTAGATACA  1318
1728mer_11         AAACAAAAGACCCTGTTTATACACCTATGGGCGTTTTCATCACTGCATGGGCTAGATACA  1318
1728mer_12         AAACAAAAGACCCTGTTTATACACCTATGGGCGTTTTCATCACTGCATGGGCTAGATACA  1318
1728mer_13         AAACAAAAGACCCTGTTTATACACCTATGGGCGTTTTCATCACTGCATGGGCTAGATACA  1318
1728mer_14         AAACAAAAGACCCTGTTTATACACCTATGGGCGTTTTCATCACTGCATGGGCTAGATACA  1318
1728mer_15         AAACAAAAGACCCTGTTTATACACCTATGGGCGTTTTCATCACTGCATGGGCTAGATACA  1316
1728mer_16         AAACAAAAGACCCTGTTTATACACCTATGGGCGTTTTCATCACTGCATGGGCTAGATACA  1319
                   ******************************************************* ****

1728mer_Reference  cgacaattacagcggcacaggcttgttatgatcggataatatactgtgatactgacagca  1378
1728mer_1          CGACAATTACAGCGGCACAGGCTTGTTATGATCGGATAATATACTGTGATACTGACAGCA  1378
1728mer_2          CGACAATTACAGCGGCACAGGCTTGTTATGATCGGATAATATACTGTGATACTGACAGCA  1375
1728mer_3          CGACAATTACAGCGGCACAGGCTTGTTATGATCGGATAATATACTGTGATACTGACAGCA  1379
1728mer_4          CGACAATTACAGCGGCACAGGCTTGTTATGATCGGATAATATACTGTGATACTGACAGCA  1377
1728mer_5          CGACAATTACAGCGGCACAGGCTTGTTATGATCGGATAATATACTGTGATACTGACAGCA  1378
1728mer_6          CGACAATTACAGCGGCACAGGCTTGTTATGATCGGATAATATACTGTGATACTGACAGCA  1377
1728mer_7          CGACAATTACAGCGGCACAGGCTTGTTATGATCGGATAATATACTGTGATACTGACAGCA  1378
1728mer_9          CGACAATTACAGCGGCACAGGCTTGTTATGATCGGATAATATACTGTGATACTGACAGCA  1378
1728mer_8          CGACAATTACAGCGGCACAGGCTTGTTATGATCGGATAATATACTGTGATACTGACAGCA  1378
1728mer_10         CGACAATTACAGCGGCACAGGCTTGTTATGATCGGATAATATACTGTGATACTGACAGCA  1378
1728mer_11         CGACAATTACAGCGGCACAGGCTTGTTATGATCGGATAATATACTGTGATACTGACAGCA  1378
1728mer_12         CGACAATTACAGCGGCACAGGCTTGTTATGATCGGATAATATACTGTGATACTGACAGCA  1378
1728mer_13         CGACAATTACAGCGGCACAGGCTTGTTATGATCGGATAATATACTGTGATACTGACAGCA  1378
1728mer_14         CGACAATTACAGCGGCACAGGCTTGTTATGATCGGATAATATACTGTGATACTGACAGCA  1378
1728mer_15         CGACAATTACAGCGGCACAGGCTTGTTATGATCGGATAATATACTGTGATACTGACAGCA  1376
1728mer_16         CGACAATTACAGCGGCACAGGCTTGTTATGATCGGATAATATACTGTGATACTGACAGCA  1379
                   ************************************************************

1728mer_Reference  tacatttaacgggtacagagatacctgatgtaataaaagatatagttgaccctaagaaat  1438
1728mer_1          TACATTTAACGGGTACAGAGATACCTGATGTAATAAAAGATATAGTTGACCCTAAGAAAT  1438
1728mer_2          TACATTTAACGGGTACAGAGATACCTGATGTAATAAAAGATATAGTTGACCCTAAGAAAT  1435
1728mer_3          TACATTTAACGGGTACAGAGATACCTGATGTAATAAAAGATATAGTTGACCCTAAGAAAT  1439
1728mer_4          TACATTTAACGGGTACAGAGATACCTGATGTAATAAAAGATATAGTTGACCCTAAGAAAT  1437
1728mer_5          TACATTTAACGGGTACAGAGATACCTGATGTAATAAAAGATATAGTTGACCCTAAGAAAT  1438
1728mer_6          TACATTTAACGGGTACAGAGATACCTGATGTAATAAAAGATATAGTTGACCCTAAGAAAT  1437
1728mer_7          TACATTTAACGGGTACAGAGATACCTGATGTAATAAAAGATATAGTTGACCCTAAGAAAT  1438
```

```
1728mer_9          TACATTTAACGGGTACAGAGATACCTGATGTAATAAAAGATATAGTTGACCCTAAGAAAT  1438
1728mer_8          TACATTTAACGGGTACAGAGATACCTGATGTAATAAAAGATATAGTTGACCCTAAGAAAT  1438
1728mer_10         TACATTTAACGGGTACAGAGATACCTGATGTAATAAAAGATATAGTTGACCCTAAGAAAT  1438
1728mer_11         TACATTTAACGGGTACAGAGATACCTGATGTAATAAAAGATATAGTTGACCCTAAGAAAT  1438
1728mer_12         TACATTTAACGGGTACAGAGATACCTGATGTAATAAAAGATATAGTTGACCCTAAGAAAT  1438
1728mer_13         TACATTTAACGGGTACAGAGATACCTGATGTAATAAAAGATATAGTTGACCCTAAGAAAT  1438
1728mer_14         TACATTTAACGGGTACAGAGATACCTGATGTAATAAAAGATATAGTTGACCCTAAGAAAT  1438
1728mer_15         TACATTTAACGGGTACAGAGATACCTGATGTAATAAAAGATATAGTTGACCCTAAGAAAT  1436
1728mer_16         TACATTTAACGGGTACAGAGATACCTGATGTAATAAAAGATATAGTTGACCCTAAGAAAT  1439
                   ************************************************************

1728mer_Reference  tgggatactgggcacatgaaagtacattcaaaagagctaaatatctgagacagaagacct  1498
1728mer_1          TGGGATACTGGGCACATGAAAGTACATTCAAAAGAGCTAAATATCTGAGACAGAAGACCT  1498
1728mer_2          TGGGATACTGGGCACATGAAAGTACATTCAAAAGAGCTAAATATCTGAGACAGAAGACCT  1495
1728mer_3          TGGGATACTGGGCACATGAAAGTACATTCAAAAGAGCTAAATATCTGAGACAGAAGACCT  1499
1728mer_4          TGGGATACTGGGCACATGAAAGTACATTCAAAAGAGCTAAATATCTGAGACAGAAGACCT  1497
1728mer_5          TGGGATACTGGGCACATGAAAGTACATTCAAAAGAGCTAAATATCTGAGACAGAAGACCT  1498
1728mer_6          TGGGATACTGGGCACATGAAAGTACATTCAAAAGAGCTAAATATCTGAGACAGAAGACCT  1497
1728mer_7          TGGGATACTGGGCACATGAAAGTACATTCAAAAGAGCTAAATATCTGAGACAGAAGACCT  1498
1728mer_9          TGGGATACTGGGCACATGAAAGTACATTCAAAAGAGCTAAATATCTGAGACAGAAGACCT  1498
1728mer_8          TGGGATACTGGGCACATGAAAGTACATTCAAAAGAGCTAAATATCTGAGACAGAAGACCT  1498
1728mer_10         TGGGATACTGGGCACATGAAAGTACATTCAAAAGAGCTAAATATCTGAGACAGAAGACCT  1498
1728mer_11         TGGGATACTGGGCACATGAAAGTACATTCAAAAGAGCTAAATATCTGAGACAGAAGACCT  1498
1728mer_12         TGGGATACTGGGCACATGAAAGTACATTCAAAAGAGCTAAATATCTGAGACAGAAGACCT  1498
1728mer_13         TGGGATACTGGGCACATGAAAGTACATTCAAAAGAGCTAAATATCTGAGACAGAAGACCT  1498
1728mer_14         TGGGATACTGGGCACATGAAAGTACATTCAAAAGAGCTAAATATCTGAGACAGAAGACCT  1498
1728mer_15         TGGGATACTGGGCACATGAAAGTACATTCAAAAGAGCTAAATATCTGAGACAGAAGACCT  1496
1728mer_16         TGGGATACTGGGCACATGAAAGTACATTCAAAAGAGCTAAATATCTGAGACAGAAGACCT  1499
                   ************************************************************

1728mer_Reference  atatacaagacatctatatgaaagaagtagatggtaagttagtagaaggtagtccagatg  1558
1728mer_1          ATATACAAGACATCTATATGAAAGAAGTAGATGGTAAGTTAGTAGAAGGTAGTCCAGATG  1558
1728mer_2          ATATACAAGACATCTATATGAAAGAAGTAGATGGTAAGTTAGTAGAAGGTAGTCCAGATG  1555
1728mer_3          ATATACAAGACATCTATATGAAAGAAGTAGATGGTAAGTTAGTAGAAGGTAGTCCAGATG  1559
1728mer_4          ATATACAAGACATCTATATGAAAGAAGTAGATGGTAAGTTAGTAGAAGGTAGTCCAGATG  1557
1728mer_5          ATATACAAGACATCTATATGAAAGAAGTAGATGGTAAGTTAGTAGAAGGTAGTCCAGATG  1558
1728mer_6          ATATACAAGACATCTATATGAAAGAAGTAGATGGTAAGTTAGTAGAAGGTAGTCCAGATG  1557
1728mer_7          ATATACAAGACATCTATATGAAAGAAGTAGATGGTAAGTTAGTAGAAGGTAGTCCAGATG  1558
1728mer_9          ATATACAAGACATCTATATGAAAGAAGTAGATGGTAAGTTAGTAGAAGGTAGTCCAGATG  1558
1728mer_8          ATATACAAGACATCTATATGAAAGAAGTAGATGGTAAGTTAGTAGAAGGTAGTCCAGATG  1558
1728mer_10         ATATACAAGACATCTATATGAAAGAAGTAGATGGTAAGTTAGTAGAAGGTAGTCCAGATG  1558
1728mer_11         ATATACAAGACATCTATATGAAAGAAGTAGATGGTAAGTTAGTAGAAGGTAGTCCAGATG  1558
1728mer_12         ATATACAAGACATCTATATGAAAGAAGTAGATGGTAAGTTAGTAGAAGGTAGTCCAGATG  1558
1728mer_13         ATATACAAGACATCTATATGAAAGAAGTAGATGGTAAGTTAGTAGAAGGTAGTCCAGATG  1558
1728mer_14         ATATACAAGACATCTATATGAAAGAAGTAGATGGTAAGTTAGTAGAAGGTAGTCCAGATG  1558
1728mer_15         ATATACAAGACATCTATATGAAAGAAGTAGATGGTAAGTTAGTAGAAGGTAGTCCAGATG  1556
1728mer_16         ATATACAAGACATCTATATGAAAGAAGTAGATGGTAAGTTAGTAGAAGGTAGTCCAGATG  1559
                   ************************************************************

1728mer_Reference  attacactgatataaaatttagtgttaaatgtgcgggaatgactgacaagattaagaaag  1618
```

```
1728mer_1          ATTACACTGATATAAAATTTAGTGTTAAATGTGCGGGAATGACTGACAAGATTAAGAAAG  1618
1728mer_2          ATTACACTGATATAAAATTTAGTGTTAAATGTGCGGGAATGACTGACAAGATTAAGAAAG  1615
1728mer_3          ATTACACTGATATAAAATTTAGTGTTAAATGTGCGGGAATGACTGACAAGATTAAGAAAG  1619
1728mer_4          ATTACACTGATATAAAATTTAGTGTTAAATGTGCGGGAATGACTGACAAGATTAAGAAAG  1617
1728mer_5          ATTACACTGATATAAAATTTAGTGTTAAATGTGCGGGAATGACTGACAAGATTAAGAAAG  1618
1728mer_6          ATTACACTGATATAAAATTTAGTGTTAAATGTGCGGGAATGACTGACAAGATTAAGAAAG  1617
1728mer_7          ATTACACTGATATAAAATTTAGTGTTAAATGTGCGGGAATGACTGACAAGATTAAGAAAG  1618
1728mer_9          ATTACACTGATATAAAATTTAGTGTTAAATGTGCGGGAATGACTGACAAGATTAAGAAAG  1618
1728mer_8          ATTACACTGATATAAAATTTAGTGTTAAATGTGCGGGAATGACTGACAAGATTAAGAAAG  1618
1728mer_10         ATTACACTGATATAAAATTTAGTGTTAAATGTGCGGGAATGACTGACAAGATTAAGAAAG  1618
1728mer_11         ATTACACTGATATAAAATTTAGTGTTAAATGTGCGGGAATGACTGACAAGATTAAGAAAG  1618
1728mer_12         ATTACACTGATATAAAATTTAGTGTTAAATGTGCGGGAATGACTGACAAGATTAAGAAAG  1618
1728mer_13         ATTACACTGATATAAAATTTAGTGTTAAATGTGCGGGAATGACTGACAAGATTAAGAAAG  1618
1728mer_14         ATTACACTGATATAAAATTTAGTGTTAAATGTGCGGGAATGACTGACAAGATTAAGAAAG  1618
1728mer_15         ATTACACTGATATAAAATTTAGTGTTAAATGTGCGGGAATGACTGACAAGATTAAGAAAG  1616
1728mer_16         ATTACACTGATATAAAATTTAGTGTTAAATGTGCGGGAATGACTGACAAGATTAAGAAAG  1619
                   ************************************************************

1728mer_Reference  aggttacgtttgagaatttcaaagtcggattcagtcggaaaatgaagcctaagcctgtgc  1678
1728mer_1          AGGTTACGTTTGAGAATTTCAAAGTCGGATTCAGTCGGAAAATGAAGCCTAAGCCTGTGC  1678
1728mer_2          AGGTTACGTTTGAGAATTTCAAAGTCGGATTCAGTCGGAAAATGAAGCCTAAGCCTGTGC  1675
1728mer_3          AGGTTACGTTTGAGAATTTCAAAGTCGGATTCAGTCGGAAAATGAAGCCTAAGCCTGTGC  1679
1728mer_4          AGGTTACGTTTGAGAATTTCAAAGTCGGATTCAGTCGGAAAATGAAGCCTAAGCCTGTGC  1677
1728mer_5          AGGTTACGTTTGAGAATTTCAAAGTCGGATTCAGTCGGAAAATGAAGCCTAAGCCTGTGC  1678
1728mer_6          AGGTTACGTTTGAGAATTTCAAAGTCGGATTCAGTCGGAAAATGAAGCCTAAGCCTGTGC  1677
1728mer_7          AGGTTACGTTTGAGAATTTCAAAGTCGGATTCAGTCGGAAAATGAAGCCTAAGCCTGTGC  1678
1728mer_9          AGGTTACGTTTGAGAATTTCAAAGTCGGATTCAGTCGGAAAATGAAGCCTAAGCCTGTGC  1678
1728mer_8          AGGTTACGTTTGAGAATTTCAAAGTCGGATTCAGTCGGAAAATGAAGCCTAAGCCTGTGC  1678
1728mer_10         AGGTTACGTTTGAGAATTTCAAAGTCGGATTCAGTCGGAAAATGAAGCCTAAGCCTGTGC  1678
1728mer_11         AGGTTACGTTTGAGAATTTCAAAGTCGGATTCAGTCGGAAAATGAAGCCTAAGCCTGTGC  1678
1728mer_12         AGGTTACGTTTGAGAATTTCAAAGTCGGATTCAGTCGGAAAATGAAGCCTAAGCCTGTGC  1678
1728mer_13         AGGTTACGTTTGAGAATTTCAAAGTCGGATTCAGTCGGAAAATGAAGCCTAAGCCTGTGC  1678
1728mer_14         AGGTTACGTTTGAGAATTTCAAAGTCGGATTCAGTCGGAAAATGAAGCCTAAGCCTGTGC  1678
1728mer_15         AGGTTACGTTTGAGAATTTCAAAGTCGGATTCAGTCGGAAAATGAAGCCTAAGCCTGTGC  1676
1728mer_16         AGGTTACGTTTGAGAATTTCAAAGTCGGATTCAGTCGGAAAATGAAGCCTAAGCCTGTGC  1679
                   ************************************************************

1728mer_Reference  aagtgccgggcggggtggttctggttgatgacacattcacaatcaaataa    1728
1728mer_1          AAGTGCCGGGCGGGGTGGTTCTGGTTGATGACACATTCACAATCAAATAA    1728
1728mer_2          AAGTGCCGGGCGGGGTGGTTCTGGTTGATGACACATTCACAATCAAATAA    1725
1728mer_3          AAGTGCCGGGCGGGGTGGTTCTGGTTGATGACACATTCACAATCAAATAA    1729
1728mer_4          AAGTGCCGGGCGGGGTGGTTCTGGTTGATGACACATTCACAATCAAATAA    1727
1728mer_5          AAGTGCCGGGCGGGGTGGTTCTGGTTGATGACACATTCACAATCAAATAA    1728
1728mer_6          AAGTGCCGGGCGGGGTGGTTCTGGTTGATGACACATTCACAATCAAATAA    1727
1728mer_7          AAGTGCCGGGCGGGGTGGTTCTGGTTGATGACACATTCACAATCAAATAA    1728
1728mer_9          AAGTGCCGGGCGGGGTGGTTCTGGTTGATGACACATTCACAATCAAATAA    1728
1728mer_8          AAGTGCCGGGCGGGGTGGTTCTGGTTGATGACACATTCACAATCAAATAA    1728
1728mer_10         AAGTGCCGGGCGGGGTGGTTCTGGTTGATGACACATTCACAATCAAATAA    1728
1728mer_11         AAGTGCCGGGCGGGGTGGTTCTGGTTGATGACACATTCACAATCAAATAA    1728
1728mer_12         AAGTGCCGGGCGGGGTGGTTCTGGTTGATGACACATTCACAATCAAATAA    1728
```

```
1728mer_13          AAGTGCCGGGCGGGGTGGTTCTGGTTGATGACACATTCACAATCAAATAA    1728
1728mer_14          AAGTGCCGGGCGGGGTGGTTCTGGTTGATGACACATTCACAATCAAATAA    1728
1728mer_15          AAGTGCCGGGCGGGGTGGTTCTGGTTGATGACACATTCACAATCAAATAA    1726
1728mer_16          AAGTGCCGGGCGGGGTGGTTCTGGTTGATGACACATTCACAATCAAATAA    1729
                    **************************************************
```

Sequencing results of 399-mer and 401-mer synthesized on CPG with reduced loading

The work has been reported in the paper *Beilstein Journal of Organic Chemistry*, **2023**, *19*, 1957-1965. https://doi.org/10.3762/bjoc.19.146. However, only 6 out of 20 sequencing results were reported in that paper. All the 20 sequencing results are now presented below for comparison with those of the present work. The number in the name of the sequences corresponds to the number of lanes in Figure 3 of the above referenced paper. For the 399-mer, sequence "contig_399-26" is correct. For the 401-mer, sequence "contig_401-10" is correct.

399-mer

```
399-reference   TAAACCCCTCCGTTTTAGAGAGGGGTTATGCTAGTTATTTGTAGAGCTCATCCATGCCAT
contig_399-1    ------------------------------------------------------------
contig_399-5    TAAACCCCTCCGTTTTAGAGAGGGGTTATGCTAGTTATTTGTAGAGCTCATCCATGCCAT
contig_399-7    ------------------------------------------------------------
contig_399-12   ------------------------------------------------------------
contig_399-13   TAAACCCCTCCGTTTTAGAGAGGGGTTATGCTAGTTATTTGTAGAGCTCATCCATGCCAT
contig_399-15   TAAACCCCTCCGTTTTAGAGAGGGGTTATGCTAGTTATTTGTAGAGCTCATCCATGCCAT
contig_399-19   ------------------------------------------------------------
contig_399-22   TAAACCCCTCCGTTTTAGAGAGGGGTTATGCTAGTTATTTGTA-AGCTCATCCATGCCAT
contig_399-26   TAAACCCCTCCGTTTTAGAGAGGGGTTATGCTAGTTATTTGTAGAGCTCATCCATGCCAT
contig_399-30   ------------------------------------------------------------

399-reference   GTGTAATCCCAGCAGCAGTTACAAACTCAAGAAGGACCATGTGGTCACGCTTTTCGTTGG
contig_399-1    ---------------------------------------------------TTTCGTTGG
contig_399-5    GTGTAATCCCAGCAGCAGTTACAAACTCAAGAAGGACCATGT-GTCACGCTTTTCGTTGG
contig_399-7    ------------------TTACAAACTCAAGAAGGACCATGTGGTCACGCTTTTCGTTGG
contig_399-12   -----------------------------------------------CGCTTTTCGTTGG
contig_399-13   GTGTAATCCCAGCAGCAGTTACAAACTCAAGAAGGACCATGTGGTCACGCTTTTCGTTGG
contig_399-15   GTGTAATCCCAGCAGCAGTTACAAACTCAAGAAGGACCATGTGGTCACGCTTTTCGTTGG
contig_399-19   -------------------------------------CATGTGGTCACGCTTTTCGTTGG
contig_399-22   GTGTAATCCCAGCAGCAGTTACAAACTCAAGAAGGACCATGTGGTCACGC----------
contig_399-26   GTGTAATCCCAGCAGCAGTTACAAACTCAAGAAGGACCATGTGGTCACGCTTTTCGTTGG
contig_399-30   ------------------------------------------------------------

399-reference   GATCTTTCGAAAGGGCAGATTGTGTCGACAGGTAATGGTTGTCTGGTAAAAGGACAGGGC
contig_399-1    GATCTTTCGAAAGGGCAGATTGTGTCGACAGGTAATGGTTGTCTGGTAAAAGGACAGGGC
contig_399-5    GATCTTTCGAAAGGGCAGA-TGTGTCGACAGGTAATGGTTGTCTGGTAAAAGGACAGGGC
contig_399-7    GATCTTTCGAAAGGGCAGATTGTGTCGACAGGTAATGGTTGTCTGGTAAAAGGACAGGGC
contig_399-12   GATCTTTCGAAAGGGCAGATTGTGTCGACAGGTAATGGTTGTCTGGTAAAAGGACAGGGC
contig_399-13   GATCTTTCGAAAGGGCAGATTGTGTCGACAGGTAATGGTTGTCTGGTAAAAGGACAGGGC
contig_399-15   GATCTTTCGAAAGGGCAGATTGTGTCGAC-GGTAATGGTTGTCTGGTAAAAGGACAGGGC
contig_399-19   GATCTTTCGAAAGGGCAGATTGTGTCGACAGGTAATGGTTGTCTGGTAAAAGGACAGGGC
contig_399-22   ------------------------------------------------------------
contig_399-26   GATCTTTCGAAAGGGCAGATTGTGTCGACAGGTAATGGTTGTCTGGTAAAAGGACAGGGC
contig_399-30   ------------------------------------------------------------

399-reference   CATCGCCAATTGGAGTATTTTGTTGATAATGGTCTGCTAGTTGAACGGATCCATCTTCAA
contig_399-1    CATCG-CAATTGGAGTATTTTGTTGATAATGGTCTGCTAGTTGAACGGATCCATCTTCAA
contig_399-5    CATCGCCAATTGGAGTATTTTGTTGATAATGGTCTGCTAGTTGAACGGATCCATCTTCAA
contig_399-7    CATCGCCAATTGGAGTATTTTGTTGATAATGGTCTGCTAGTTGAACGGATCCATCTTCAA
contig_399-12   CATCGCCAATTGGAGTATTTTGTTGATAATGGTCTGCTAGTTGAACGGATCCATCTTCAA
contig_399-13   CATCGCCAATTGGAGTATTTTGTTGATAATGGTCTGCTAGTTGAACGGATCCATCTTCAA
contig_399-15   CATCGCCAATTGGAGTATTTTGTTGATAATGGTCTGCTAGTTGAACGGATCCATCTTCAA
contig_399-19   CATCGCCAATTGGAGTATTTTGTTGATAATGGTCTGCTAGTTGAACGGATCCATCTTCAA
contig_399-22   ------------------------------------------------------------
contig_399-26   CATCGCCAATTGGAGTATTTTGTTGATAATGGTCTGCTAGTTGAACGGATCCATCTTCAA
contig_399-30   ------------------------------------------------ATCCATCTTCAA
```

```
399-reference      TGTTGTGGCGAATTTTGAAGTTAGCTTTGATTCCATTCTTTTGTTTGTCTGCCGTGATGT
contig_399-1       TGTTGTGGCGAATTTTGAAGTTAGCTTTGATTCCATTCTTTTGTTTGTCTGCCGTGATGT
contig_399-5       T------------------GTTAGCTTTGATTCCATTCTTTTGTTTGTCTGCCGTGATGT
contig_399-7       TGTTGTGGCGAATTTTGAAGTTAGCTTTGATTCCATTCTTTTGTTTGTCTGCCGTGATGT
contig_399-12      TGTTGTGGCGAATTTTGAAGTTAGCTTTGATTCCATTCTTTTGTTTGTCTGCCGTGATGT
contig_399-13      TGTTGTGGCGAATTTTGAAGTTAGCTTTGATTCCATTCTTTTGTTTGTCTGCCGTGATGT
contig_399-15      TGTTGTGGCGAATTTTGAAGTTAGCTTTGATTCCATTCTTTTGTTTGTCTGCCGTGATGT
contig_399-19      TGTTGTGGCGAATTTTGAAGTTAGCTTTGATTCCATTCTTTTGTTTGTCTGCCGTGATGT
contig_399-22      --------------------------------------------------------ATGT
contig_399-26      TGTTGTGGCGAATTTTGAAGTTAGCTTTGATTCCATTCTTTTGTTTGTCTGCCGTGATGT
contig_399-30      TGTTGTGGCGAATTTTGAAGTTAGCTTTGATTCCATTCTTTTGTTTGTCTGCCGTGATGT

399-reference      ATACATTGTGTGAGTTATAGTTGTACTCGAGTTTGTGTCCGAGAATGTTTCCATCTTCTT
contig_399-1       ATACATTGTGTGAGTTATAGTTGTACTCGAGTTTGTGTCCGAGAATGTTTCCATCTTCTT
contig_399-5       ATACATTGTGTGAGTTATAGTTGTACTCGAGTTTGTGTCCGAGAATGTTTCCATCTTCTT
contig_399-7       ATACATTGTGTGAGTTATAGTTGTACTCGAGTTTGTGTCCGAGAATGTTTCCATCTTCTT
contig_399-12      ATACATTGTGTGAGTTATAGTTGTACTCGAGTTTGTGTCCGAGAATGTTTCCATCTTCTT
contig_399-13      ATACATTGTGTaAGTTATAGTTGTACTCGAGTTTGTGTCCGAGAATGTTTCCATCTTCTT
contig_399-15      ATACATTGTGTGAGTTATAGTTGTACTCGAGTTTGTGTCCGAGAATGTTTCCATCTTCTT
contig_399-19      ATACATTGTGTGAGTTATAGTTGTACTCGAGTTTGTGTCCGAGAATGTTTCCATCTTCTT
contig_399-22      ATACATTGTGTGAGTTATAGTTGTACTCGAGTTTGTGTCCGAGAATGTTTCCATCTTCTT
contig_399-26      ATACATTGTGTGAGTTATAGTTGTACTCGAGTTTGTGTCCGAGAATGTTTCCATCTTCTT
contig_399-30      ATACATTGTGTGAGTTATAGTTGTACTCGAGTTTGTGTCCGAGAATGTTTCCATCTTCTT

399-reference      TAAAATCAATACCTTTTAACTCGATACGATTAACAAGGG
contig_399-1       TAAAATCAATACCTTTTAACTCGATACGATTAACAAGGG
contig_399-5       TAAAATCAATACCTTTTAACTCGATACGATTAACAAGGG
contig_399-7       TAAAATCAATACCTTTTAACTCGATACGATTAACAAGGG
contig_399-12      TAAAATCAATACCTTTTAACTCGATACGATTAACAAGGG
contig_399-13      TAAAATCAATACCTTTTAACTCGATACGATTAACAAGGG
contig_399-15      TAAAATCAATACCTTTTAACTCGATACGATTAACAAGGG
contig_399-19      TAAAATCAATACCTTTTAACTCGATACGATTAACAAGGG
contig_399-22      TAAAATCAATACCTTTTAACTCGATACGATTAACAAGGG
contig_399-26      TAAAATCAATACCTTTTAACTCGATACGATTAACAAGGG
contig_399-30      TAAAATCAATACCTTTTAACTCGATACGATTAACAAGGG
```

401-mer

```
401-reference      TATCACCTTCAAACTTGACTTCAGCACGCGTCTTGTAGTTCCCGTCATCTTTGAAAGATA
contig_401-1       ------------------------------------------------------------
contig_401-3       TATCACCTTCAAACTTGACTTCAGCACGCGTCTTGTAGTTCCCGTCATCTTTGAAAGATA
contig_401-6       TATCACCTTCAAACTTGACTTCAGCACGCGTCTTGTAGTTCCCGTCATCTTTGAAAGATA
contig_401-8       TATCACCTTCAAACTTGACTTCAGCACGCGTCTTG-AGTTCCCGTC-TCTTTGAAAGATA
contig_401-10      TATCACCTTCAAACTTGACTTCAGCACGCGTCTTGTAGTTCCCGTCATCTTTGAAAGATA
contig_401-14      TATCACCTTCAAACTTGACTTCAGCACGCGTCTTGTAGTTCCCGTCATCTTTGAAAGATA
contig_401-16      TATCACCTTCAAACTTGACTTCAGCACGCGTCTTGTAGTTCCCGTCATCTTTGAAAGATA
contig_401-20      TATCACCTTCAAACTTGACTTCAGCACGCGTCTTGTAGTTCCCGTCATCTTTGAAAGATA
contig_401-25      TATCACCTTCAAACTTGACTTCAGCACGCGTCTTGTAGTTCCCGTCATCTTTGAAAGATA
contig_401-31      TATCACCTTCAAACTTGACTTCAGCACGCGTCTTGTAGTTCCCGTCATCTT---------

401-reference      TAGTGCGTTCCTGTACATAACCTTCGGGCATGGCACTCTTGAAAAAGTCATGCCGTTTCA
contig_401-1       --------------------------------------TTGAAAAAGTCATGCCGTTTCA
contig_401-3       TAGTGCGTTCCTGTACATAACCTT-GGGCATGGCACTCTTGAAAAAGTCATGCCGTTTCA
contig_401-6       TAGTaCGTTCCTGTACATAACCTTCGGGCATGGCACTCTTGAAAAAGTCATGCCGTTTCA
contig_401-8       TAGTGCGTTCCTGTACATAACCTTCGGGCATGGCACTCTTGAAAAAGTCATGCCGTTTCA
contig_401-10      TAGTGCGTTCCTGTACATAACCTTCGGGCATGGCACTCTTGAAAAAGTCATGCCGTTTCA
contig_401-14      TAGTGCGTTCCTGTACATAACCTTCGGGCATGGCACTCTTGAAAAAGTCATGCCGTTTCA
contig_401-16      TAGTGCG-TCCTGTACATAACCTTCGGGCATGGCACTCTTGAAAAAGTCATGCCGTTTCA
contig_401-20      TAGTGCGTTCCTGTACATAACCTTCGGGCATGGCACTCTTGAAAAAGTCATGCCGTTTCA
contig_401-25      TAGTGCGTTCCTGTACATAACCTTCGGGCATGGCACTCTTG-AAAAGTCATGCCGTTTCA
contig_401-31      ------------------------------------------------------------
```

```
401-reference     TATGATCCGGATAACGGGAAAAGCATTGAACACCATAAGAGAAAGTAGTGACAAGTGTTG
contig_401-1      TATGATCCGGATAACGGGAAAAGCATTGAACACCATAAGAGAAAGTAGTGACAAGTGTTG
contig_401-3      TATGATCCGGATAACGGGAAAAGCATTGAACACCATAAGAGAAAGTAG------------
contig_401-6      TATGATCCGGATAACGGGAAAAGCATTGAACACCATAAGAGAAAGTAGTGACAAGTGTTG
contig_401-8      TATGATCCGG--------------------------------------------------
contig_401-10     TATGATCCGGATAACGGGAAAAGCATTGAACACCATAAGAGAAAGTAGTGACAAGTGTTG
contig_401-14     TATGATCCGGATAACGGGAAAAGCATTGAACACCATAAGAGAAAGTAGTGACAAGTGTTG
contig_401-16     TATGATCCGGATAACGGGAAAAGCATTGAACACCATAAGAGAAAGTAGTGAC-AGTGTTG
contig_401-20     TATGATCCGGATAACGGGAAAAGCATTGAACACCATAAaAGAAAGTAGTGACAAGTGTTG
contig_401-25     TAT---------------------------------------------------------
contig_401-31     ------------------------------------------------------------

401-reference     GCCATGGAACAGGTAGTTTTCCAGTAGTGCAAATAAATTTAAGGGTAAGTTTTCCGTATG
contig_401-1      GCCATGGAACAGGTAGTTTTCCAGTAGTGCAAATAAATTTAAGGGTAAGTTTTCCGTATG
contig_401-3      ------------------------------------------------------------
contig_401-6      GCCATGGAACAGGTAGTTTcCCAGTAGTGCAAATAAATTTAAGGGTAAGTTTTCCGTATG
contig_401-8      ------------------------------------------------------------
contig_401-10     GCCATGGAACAGGTAGTTTTCCAGTAGTGCAAATAAATTTAAGGGTAAGTTTTCCGTATG
contig_401-14     GCCATGGAACAGGTAGTTTTCCAGTAGTGCAAATAAA-----------------------
contig_401-16     GCCATGGAACAGGTAGTTTTCCAGTAGTGCAAATAAATTTAAGGGTAAGTTTTCCGTATG
contig_401-20     GCCATGGAACAGGTAGTTTTCCAGTAGTGCAAATAAATTTAAGGGTAAGTTTTCCGTATG
contig_401-25     ------------------------------------------------------------
contig_401-31     ------------------------------------------------------------

401-reference     TTGCATCACCTTCACCCTCTCCACTGACAGAAAATTTGTGCCCATTAACATCACCATCTA
contig_401-1      TTGCATCACCTTCACCCTCTCCACTGACAGAAAATTTGTGCCCATTAACATCACCATCTA
contig_401-3      ------------------------------------------------------------
contig_401-6      TTGCATCACCTTCACCCTCTCCACTGACAGAAAATTTGTGCCCATTAACATCA-CATCTA
contig_401-8      ------------------------------------------------------------
contig_401-10     TTGCATCACCTTCACCCTCTCCACTGACAGAAAATTTGTGCCCATTAACATCACCATCTA
contig_401-14     ------------------------------------------------------------
contig_401-16     TTGCATCACCTTCACCCTCTCCACTGACAGAAAATTTGTGCCCATTAACATCACCATCTA
contig_401-20     TTGCATCACCTTCACCCTCTCCACTGACAGAAAATTTGTGCCCATTAACATCACCATCTA
contig_401-25     ------------------------------------------------------------
contig_401-31     ------------------------------------------------------------

401-reference     ATTCAACAAGAATTGGGACAACTCCAGTGAAAAGTTCTTCTCCTTTACTCATATTTTTTC
contig_401-1      ATTCAACAAGAATTGG--------CAGTGAAAAGTTCTTCTCCTTTACTCATATTTTTTC
contig_401-3      ---------------------------------------CTCCTTTACTCATATTTTTTC
contig_401-6      ATTCAACAAGAATTGGGACAACTCCAGTGAAAAGTTCTTCTCCTTTACTCATATTTTTTC
contig_401-8      --------------------------GTGAAAAGTTCTTCTCCTTTACTCATATTTTTTC
contig_401-10     ATTCAACAAGAATTGGGACAACTCCAGTGAAAAGTTCTTCTCCTTTACTCATATTTTTTC
contig_401-14     -------------------------------------------------------TTTTC
contig_401-16     ATTCAACAAGAATTGGGACAACTCCAGTGAAAAGTTCTTCTCCTTTACTCATATTTTTTC
contig_401-20     ATTCAACAAGAATTGGGACAACTCCAGTGAAAAGTTCTTCTCCTTTACTCATATTTTTTC
contig_401-25     --------------------------------------------------ATATTTTTTC
contig_401-31     ------------------------------------CTTCTCCTTTACTCATATTTTTTC

401-reference     CTCCTTATACTTAAGCCCTATAGTGAGTCGTATTAATTCGC
contig_401-1      CTCCTTATACTTAAGCCCTATAGTGAGTCGTATTAATTCGC
contig_401-3      CTCCTTATACTTAAGCCCTATAGTGAGTCGTATTAATTCGC
contig_401-6      CTCCTTATACTTAAGCCCTATAGTGAGTCGTATTAATTCGC
contig_401-8      CTCCTTATACTTAAGCCCTATAGTGAGTCGTATTAATTCGC
contig_401-10     CTCCTTATACTTAAGCCCTATAGTGAGTCGTATTAATTCGC
contig_401-14     CTCCTTATACTTAAGCCCTATAGTGAGTCGTATTAATTCGC
contig_401-16     CTCCTTATACTTAAGCCCTATAGTGAGTCGTATTAATTCGC
contig_401-20     CTCCTTATACTTAAGCCCTATAGTGAGTCGTATTAATTCGC
contig_401-25     CTCCTTATACTTAAGCCCTATAGTGAGTCGTATTAATTCGC
contig_401-31     CTCCTTATACTTAAGCCCTATAGTGAGTCGTATTAATTCGC
```
